# Supplementary material for: Bridging the antiviral gap: A BAC-based non-infectious SARS-CoV-2 replicon reveals potent antisense gapmers
Source: Mol Ther Nucleic Acids. 2026 Sep 15;37(4):103098. doi: 10.1016/j.omtn.2026.103098 (PMC13656313; doi:10.1016/j.omtn.2026.103098)
Supplement: Supplementary file 1 [file mmc1.pdf]

OMTN, Volume *37*

Supplemental information

Bridging the antiviral gap: A BAC-based non-infectious SARS-CoV-2 replicon reveals potent antisense gapmers

Agnieszka Ruszkowska, Agnieszka Baliga-Gil, Julita Piasecka, Maria Ciechanowska, Ryszard Kierzek, and Elzbieta Kierzek

SUPPLEMENTAL FIGURES

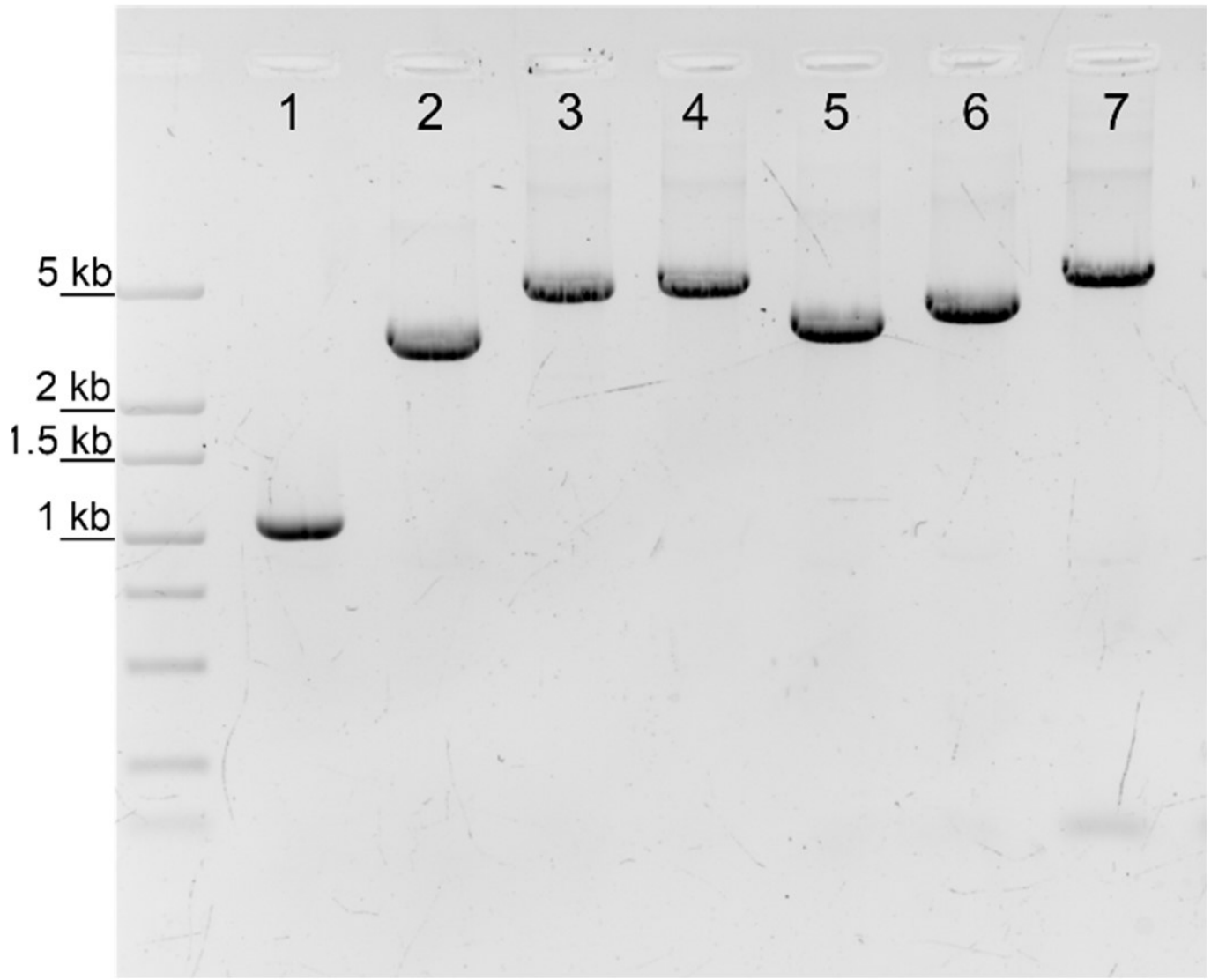

Figure S1. DNA fragments F1-F7 used to assembly pBAC-SARS-CoV-2_*S/eGFP_ΔE* non-infectious replicon. Length of the PCR amplified fragments: F1 – 1064 bp; F2 – 3150 bp; F3 – 5122 bp; F4 – 5405 bp; F5 – 3539 bp; F6 – 4131 bp; F7 – 5741 bp.

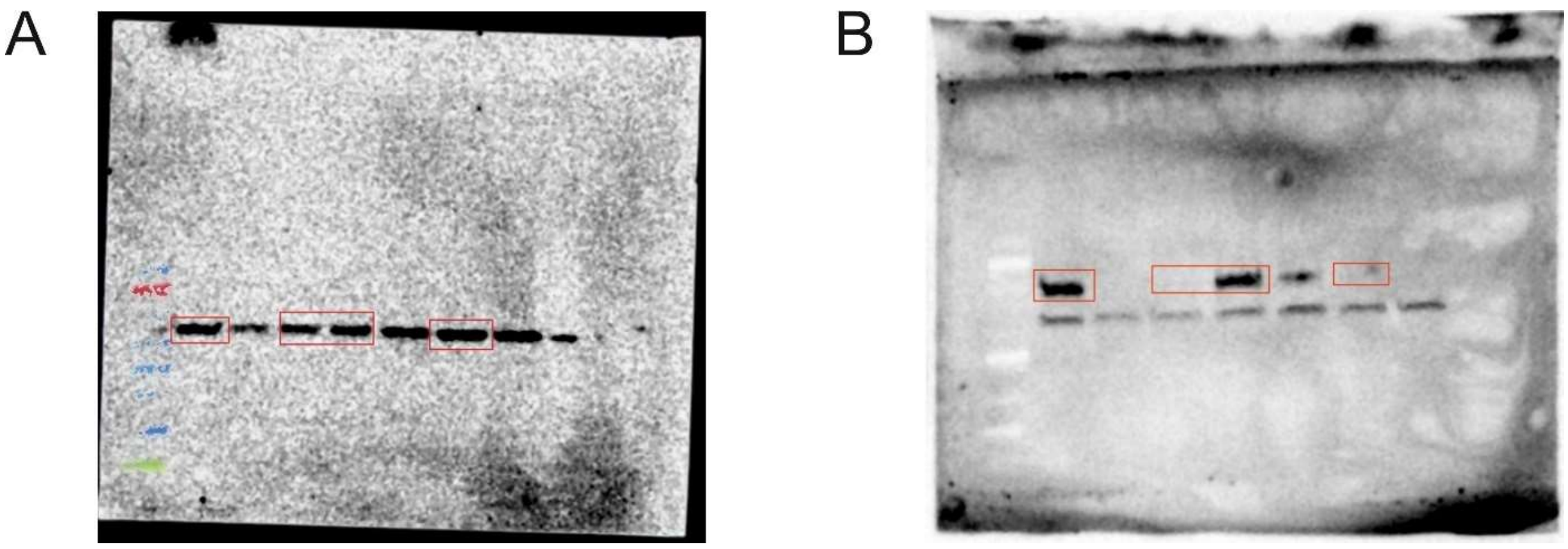

Figure S2. Uncropped Western blot images. Panel A shows the membrane probed for actin, and Panel B shows the membrane probed for the nucleocapsid protein. Red rectangles indicate the bands presented in Figure 2.

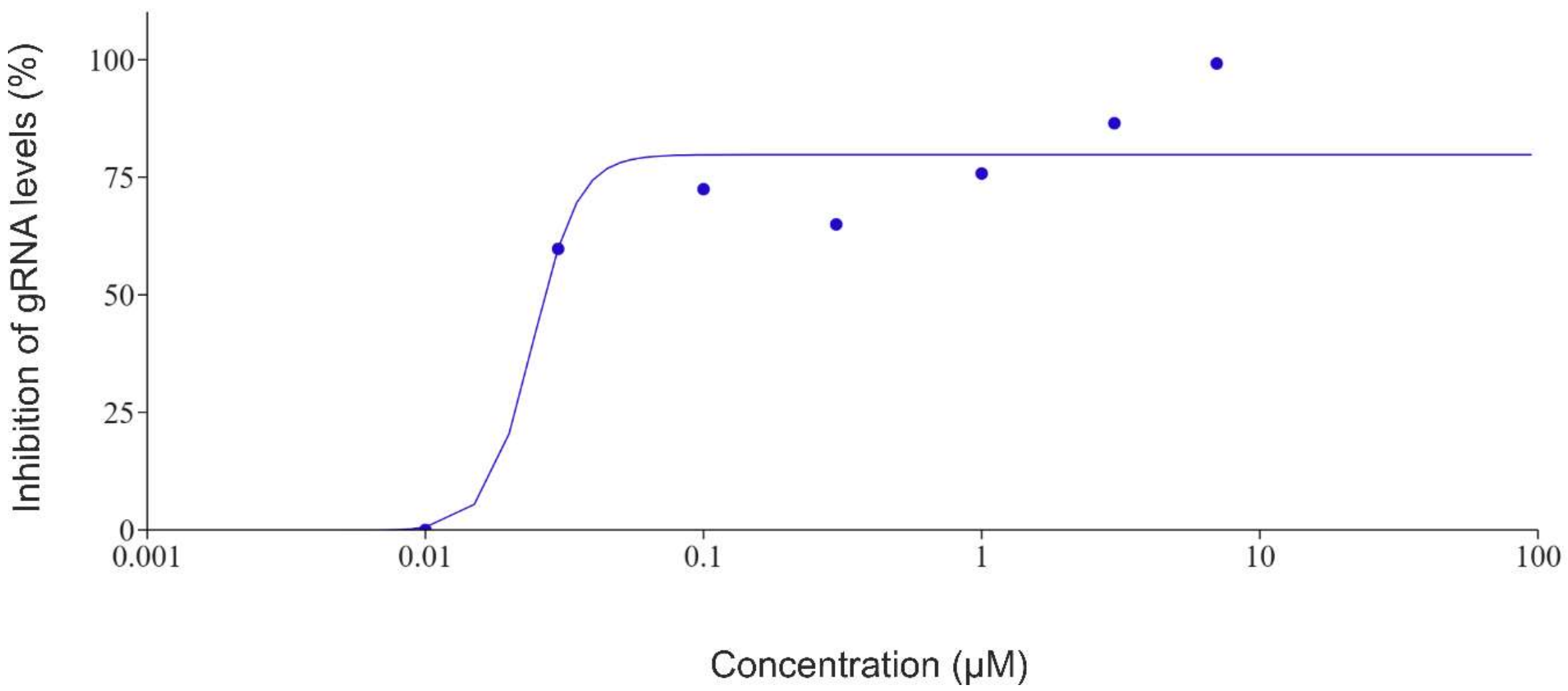

Figure S3. Dose-dependent inhibition of the pBAC-SARS-CoV-2_*S/eGFP*_*ΔE* replicon activity by remdesivir. Effect of remdesivir on replicon activity was evaluated at concentrations: 0.01, 0.03, 0.1, 0.3, 1, 3, and 2 μM. Replicon activity was quantified by RT-qPCR. The EC_{50} value was calculated using the AAT Bioquest online calculator.

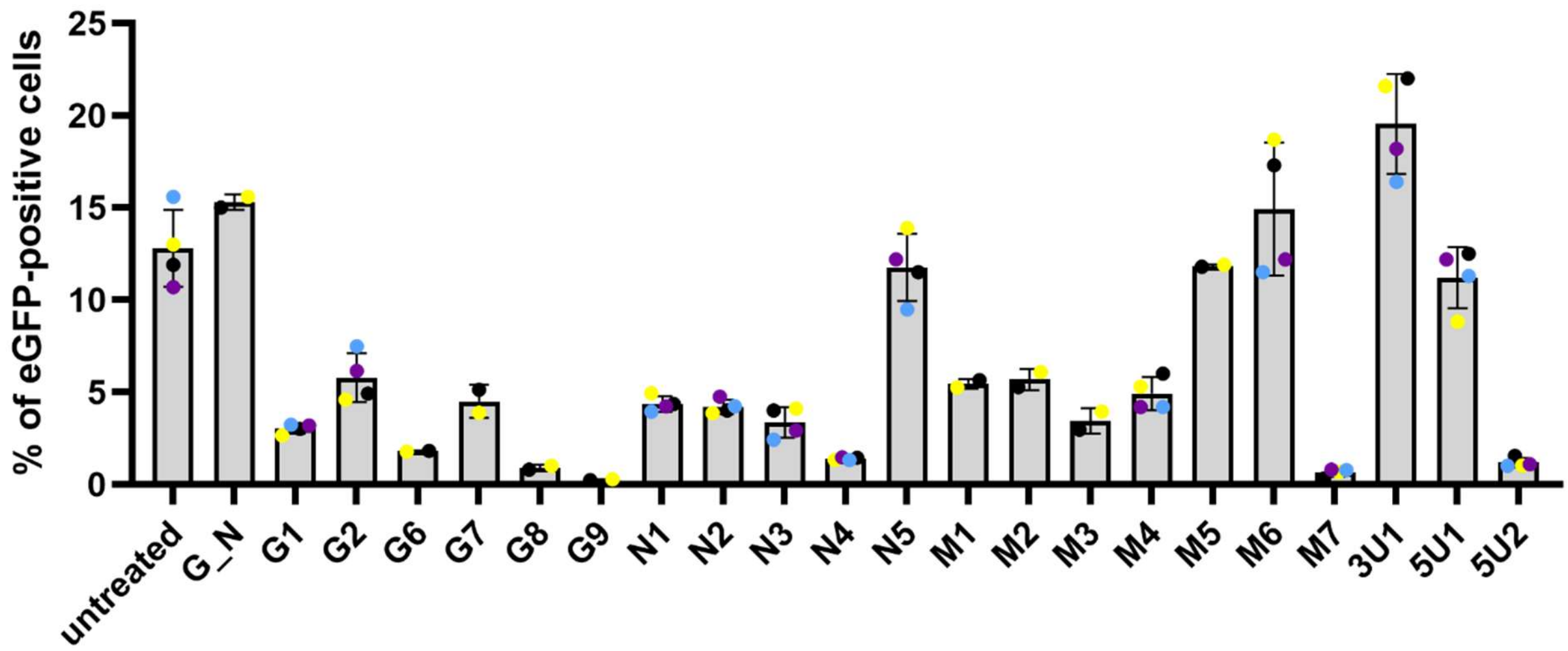

Figure S4. Effect of simultaneous co-transfection with gapmers and the pBAC-SARS-CoV-2_*S/eGFP*_*ΔE* replicon on replicon activity, assessed by the eGFP signal. Replicon activity is shown as the percentage of eGFP-positive cells among viable cells. Colored dots indicate individual experimental replicates, whereas gray bars represent the mean of all replicates.

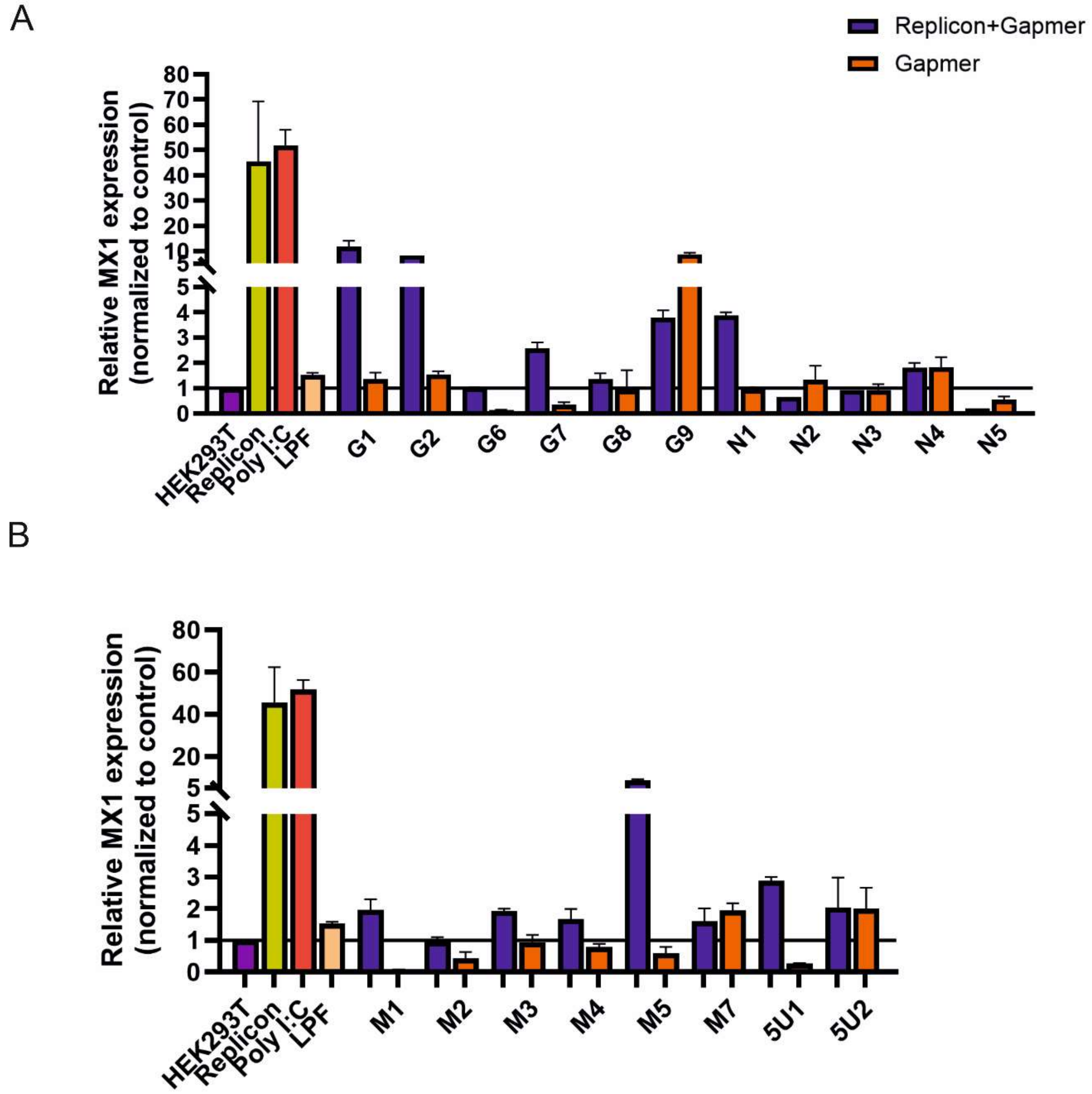

Figure S5. Expression of the interferon-stimulated gene *MX1* in HEK293T cells co-transfected with the replicon and gapmers. Bars represent the mean ± SD from two biological replicates, each with two technical replicates. Blue bars represent HEK293T cells treated with both the replicon and gapmer, whereas orange bars represent cells treated with gapmer only. Gene expression was quantified by qPCR analysis. Panel A shows results for gapmers targeting the *N* gene, whereas panel B shows results for gapmers targeting the *M* gene and UTRs. Abbreviations: Poly I:C, polyinosinic:polycytidylic acid; LPF, Lipofectamine.

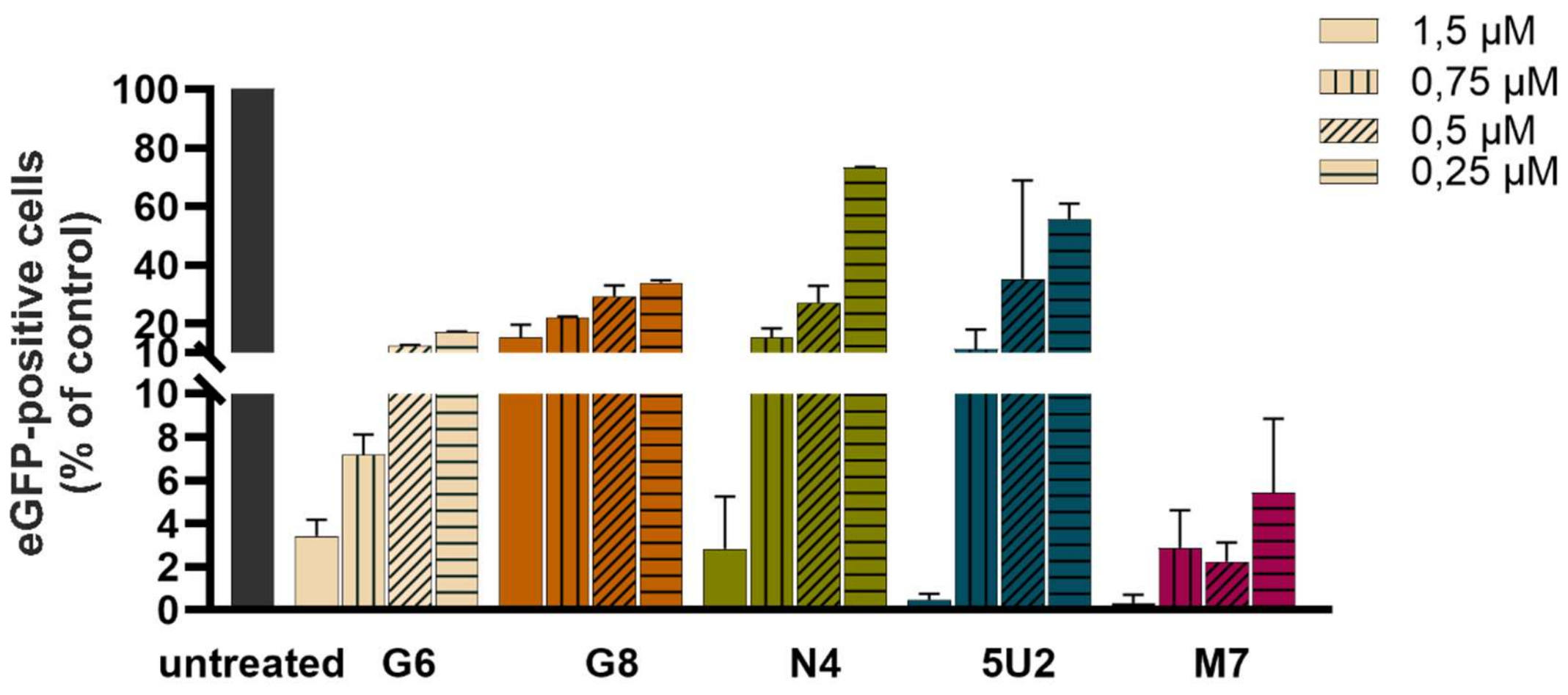

Figure S6. Dose-dependent inhibition of the pBAC-SARS-CoV-2_*S/eGFP_ΔE* replicon activity by selected gapmers. Replicon activity was quantified by flow cytometry as the percentage of eGFP-positive cells. HEK293T cells transfected with the replicon alone served as the reference control and were assigned a value of 100%. Bars represent the mean ± SD from two biological replicates, each with two technical replicates.

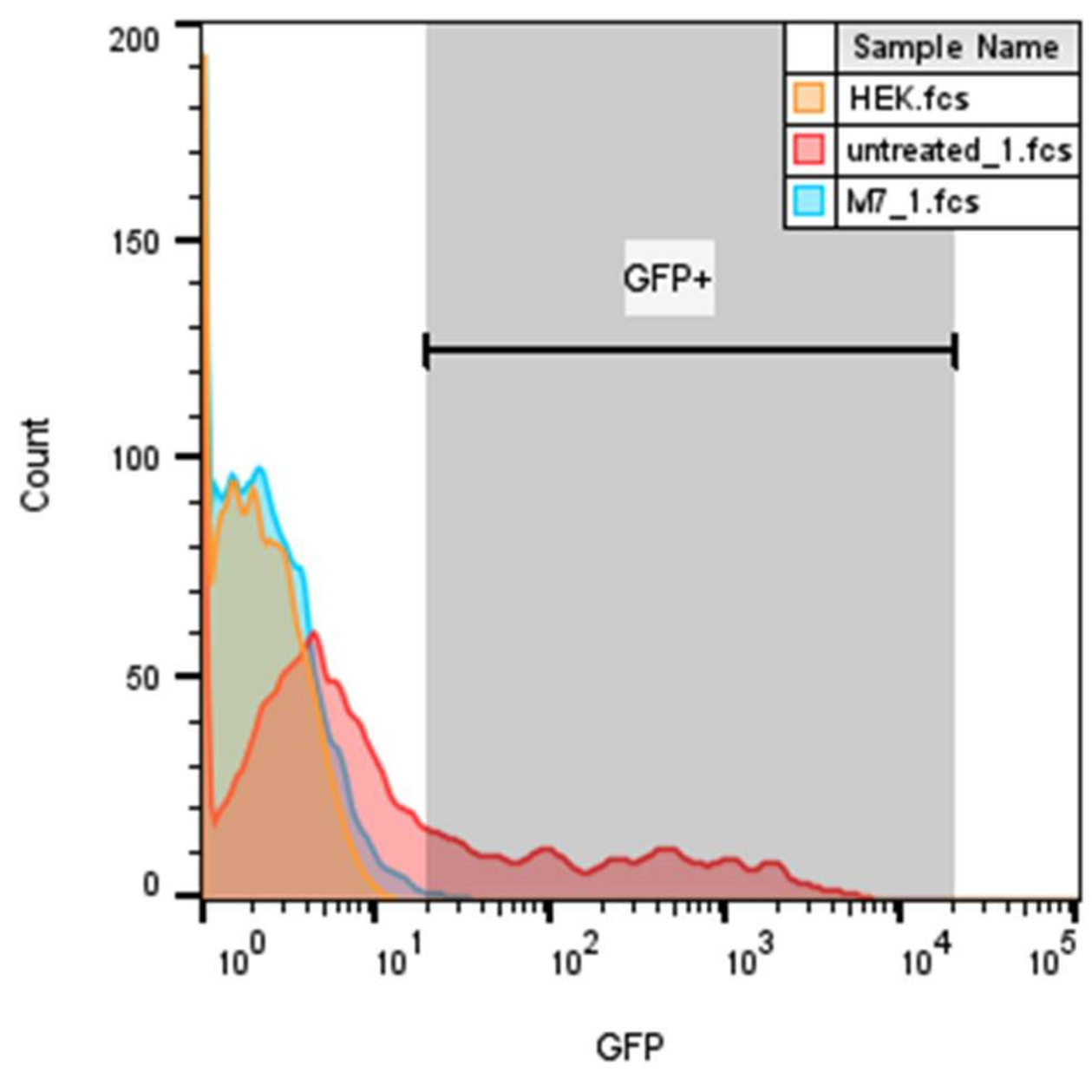

Sample Name	GFP+ Median
HEK.fcs	n/a
M7_1.fcs	31,4
untreated_1.fcs	210

Figure S7. Flow cytometry histogram showing eGFP intensity in HEK293T cells transfected with the pBAC-SARS-CoV-2_*S/eGFP_ΔE* replicon. eGFP intensity is shown for cells transfected with the replicon alone (untreated) and cells co-transfected with the replicon and gapmer M7.

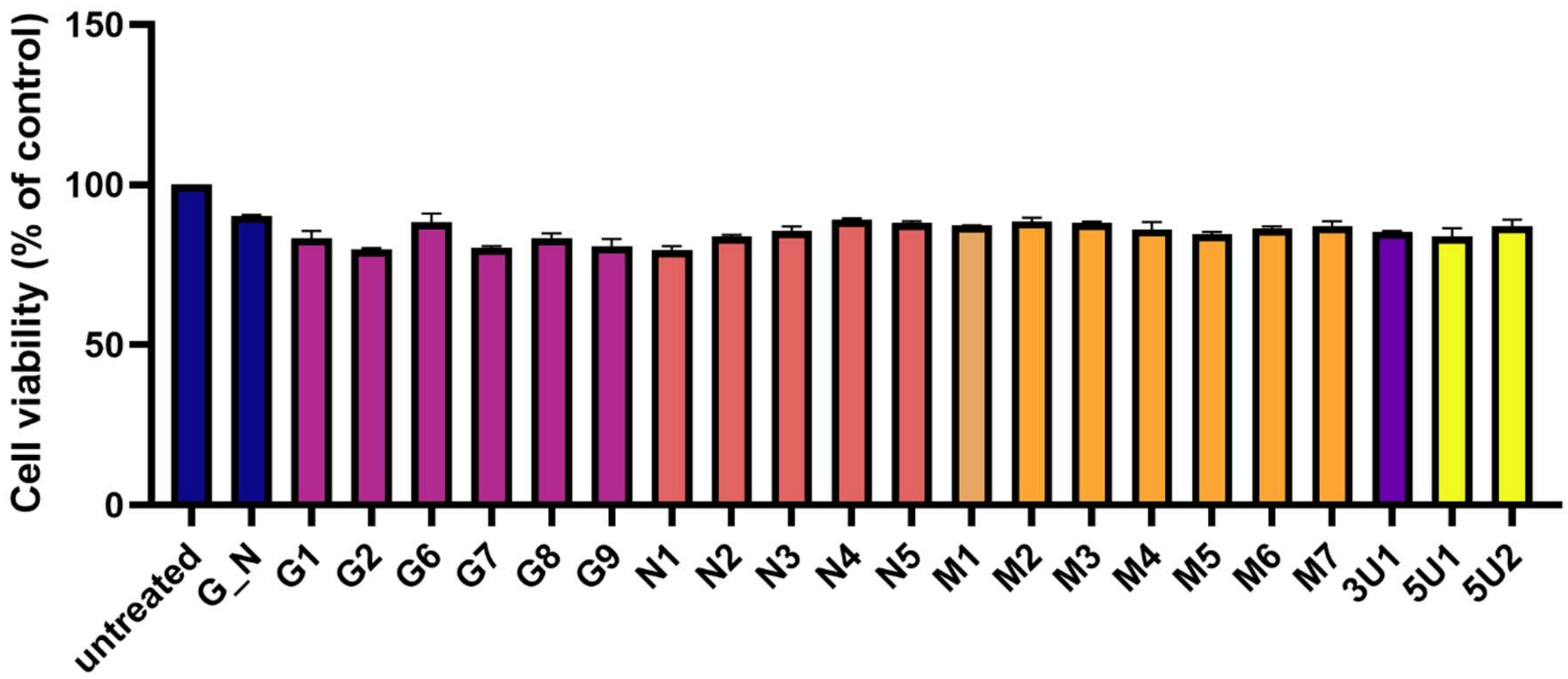

Figure S8. Viability of HEK293T cells co-transfected with the replicon and gapmers at a final gapmer concentration of 0.75 μM. The quantities of dead cells stained with propidium iodide (PI) were measured by flow cytometry, then the cell viability was analyzed based on these measurements. Experiment was performed in two biological replicates.

SUPPLEMENTAL TABLES

Table S1. Sequence of F7.1 and F7.2 used for the assembly of F7.

F7.1	AGCAACCAATGGAGATTGATTAAACGAACATGAAAATTATTCTTTTCTTGGCACTGATAACACTCGCTACTTGTGAGCTTTATCACTACCAAGAGTGTGTTAGAGGT<br>ACAACAGTACTTTTAAAAGAACCTTGCTCTTCTGGAACATACGAGGGCAATTCACCATTTCATCCTCTAGCTGATAACAAATTTGCACTGACTTGCTTTAGCACTCAA<br>TTTGCTTTTGCTTGTCCTGACGGCGTAAAACACGTCTATCAGTTACGTGCCAGATCAGTTTCACCTAAACTGTTCATCAGACAAGAGGAAGTTCAAGAACTTTACTCT<br>CCAATTTTTCTTATTGTTGCGGCAATAGTGTTTATAACACTTTGCTTCACACTCAAAAGAAAGACAGAATGATTGAACTTTCATTAATTGACTTCTATTTGTGCTTTTT<br>AGCCTTTCTGCTATTCCTTGTTTTAATTATGCTTATTATCTTTTGGTTCTCACTTGAACTGCAAGATCATAATGAAACTTGTCACGCCTAAACGAACATGAAATTTCTT<br>GTTTTCTTAGGAATCATCACAACTGTAGCTGCATTTCACCAAGAATGTAGTTTACAGTCATGTACTCAACATCAACCATATGTAGTTGATGACCCGTGTCCTATTCAC<br>TTCTATTCTAAATGGTATATTAGAGTAGGAGCTAGAAAATCAGCACCTTTAATTGAATTGTGCGTGGATGAGGCTGGTTCTAAATCACCCATTCAGTACATCGATATC<br>GGTAATTATACAGTTTCCTGTTTACCTTTTACAATTAATTGCCAGGAACCTAAATTGGGTAGTCTTGTAGTGCGTTGTTCGTTCTATGAAGACTTTTTAGAGTATCATG<br>ACGTTCGTGTTGTTTTAGATTTCATCTAAACGAACAAACTAAAATGTCTGATAATGGACCCCAAAATCAGCGAAATGCACCCCGCATTACGTTTGGTGGACCCTCAG<br>ATTCAACTGGCAGTAACCAGAATGGAGAACGCAGTGGGGCGCGATCAAAACAACGTCGGCCCCAAGGTTTACCCAATAATACTGCGTCTTGGTTCACCGCTCTCACT<br>CAACATGGCAAGGAAGACCTTAAATTCCCTCGAGGACAAGGCGTTCCAATTAACACCAATAGCAGTCCAGATGACCAAATTGGCTACTACCGAAGAGCTACCAGAC<br>GAATTCGTGGTGGTGACGGTAAAATGAAAGATCTCAGTCCAAGATGGTATTTCTACTACCTAGGAACTGGGCCAGAAGCTGGACTTCCCTATGGTGCTAACAAAGAC<br>GGCATCATATGGGTTGCAACTGAGGGAGCCTTGAATACACCAAAAGATCACATTGGCACCCGCAATCCTGCTAACAATGCTGCAATCGTGCTACAACTTCCTCAAGG<br>AACAACATTGCCAAAAGGCTTCTACGCAGAAGGGAGCAGAGGCGGCAGTCAAGCCTCTTCTCGTTCCTCATCACGTAGTCGCAACAGTTCAAGAAATTCAACTCCA<br>GGCAGCAGTAGGGGAACTTCTCCTGCTAGAATGGCTGGCAATGGCGGTGATGCTGCTCTTGCTTTGCTGCTGCTTGACAGATTGAACCAGCTTGAGAGCAAAATGTC<br>TGGTAAAGGCCAACAACAACAAGGCCAAACTGTCACTAAGAAATCTGCTGCTGAGGCTTCTAAGAAGCCTCGGCAAAAACGTACTGCCACTAAAGCATACAATGTA<br>ACACAAGCTTTCGGCAGACGTGGTCCAGAACAAACCCAAGGAAATTTTGGGGACCAGGAACTAATCAGACAAGGAACTGATTACAAACATTGGCCGCAAATTGCAC<br>AATTTGCCCCCAGCGCTTCAGCGTTCTTCGGAATGTCGCGCATTGGCATGGAAGTCACACCTTCGGGAACGTGGTTGACCTACACAGGTGCCATCAAATTGGATGAC<br>AAAGATCCAAATTTCAAAGATCAAGTCATTTTGCTGAATAAGCATATTGACGCATACAAAACATTCCCACCAACAGAGCCTAAAAAGGACAAAAAGAAGAAGGCTG<br>ATGAAACTCAAGCCTTACCGCAGAGACAGAAGAAACAGCAAACTGTGACTCTTCTTCCTGCTGCAGATTTGGATGATTTCTCCAAACAATTGCAACAATCCATGAGC<br>AGTGCTGACTCAACTCAGGCCTAAACTCATGCAGACCACACAAGGCAGATGGGCTATATAAACGTTTTCGCTTTTCCGTTTACGATATATAGTCTACTCTTGTGCAGA<br>ATGAATTCTCGTAACTACATAGCACAAGTAGATGTAGTTAACTTTAATCTCACATAGCAATCTTTAATCAGTGTGTAACATTAGGGAGGACTTGAAAGAGCCACCAC<br>ATTTTCACCGAGGCCACGCGGAGTACGATCGAGTGTACAGTGAACAATGCTAGGGAGAGCTGCCTATATGGAAGAGCCCTAATGTGTAAAATTAATTTTAGTAGTGC<br>TATCCCCATGTGATTTTAATAGCTTCTTAGGAGAATGACAAAAAAAAAGGGTCGGCATGGCATCTCCACCTCCTCGCGGTCCGACCTGGGCTACTTCGGTAGGCTAA<br>GGGAGAAGCTGTGCCTTCTAGTTGCCAGCCATCTGTTGTTTGCCCCTCCCCCGTGCCTTCCTTGACCCTGGAAGGTGCCACTCCCACTGTCCTTTCCTAATAAAATGA<br>GGAAATTGCATCGCATTGTCTGAGTAGGTGTCATTCTATTCTGGGGGGTGGGGTGGGGCAGGACAGCAAGGGGGAGGATTGGGAAGACAATAGCAGGCATGCTGGG<br>GATGCGGTGGGCTCTATGGGGCGCGCCAAGCTTGAGTATTCTATAGTGTCACCTAAATAGCTTGGCGTAATCATGGTCATAGCTGTTTCCTGTGTGAAATTGTTATCC<br>GCTCACAATTC

F7.2	AAGGTCAAATCAATGATATGATTTTATCTCTTCTTAGTAAAGGTAGACTTATAATTAGAGAAAACAACAGAGTTGTTATTTCTAGTGATGTTCTTGTTAACAACTAAA<br>CGAACAATGTTTGTTTTTCTTGTTTTATTGCCACTAgcgatcgcaATGGTGAGCAAGGGCGAGGAGCTGTTCACCGGGGTGGTGCCCATCCTGGTCGAGCTGGACGGCGAC<br>GTAAACGGCCACAAGTTCAGCGTGTCCGGCGAGGGCGAGGGCGATGCCACCTACGGCAAGCTGACCCTGAAGTTCATCTGCACCACCGGCAAGCTGCCCGTGCCCT<br>GGCCCACCCTCGTGACCACCCTGACCTACGGCGTGCAGTGCTTCAGCCGCTACCCCGACCACATGAAGCAGCACGACTTCTTCAAGTCCGCCATGCCCGAAGGCTAC<br>GTCCAGGAGCGCACCATCTTCTTCAAGGACGACGGCAACTACAAGACCCGCGCCGAGGTGAAGTTCGAGGGCGACACCCTGGTGAACCGCATCGAGCTGAAGGGC<br>ATCGACTTCAAGGAGGACGGCAACATCCTGGGGCACAAGCTGGAGTACAACTACAACAGCCACAACGTCTATATCATGGCCGACAAGCAGAAGAACGGCATCAAG<br>GTGAACTTCAAGATCCGCCACAACATCGAGGACGGCAGCGTGCAGCTCGCCGACCACTACCAGCAGAACACCCCCATCGGCGACGGCCCCGTGCTGCTGCCCGACA<br>ACCACTACCTGAGCACCCAGTCCGCCCTGAGCAAAGACCCCAACGAGAAGCGCGATCACATGGTCCTGCTGGAGTTCGTGACCGCCGCCGGGATCACTCTCGGCAT<br>GGACGAGCTGTACAAGTAATGTAGTTGTCTCAAGGGCTGTTGTTCTTGTGGATCCTGCTGCAAATTTGATGAAGACGACTCTGAGCCAGTGCTCAAAGGAGTCAAAT<br>TACATTACACATAAACGAACTTATGGATTTGTTTATGAGAATCTTCACAATTGGAACTGTAACTTTGAAGCAAGGTGAAATCAAGGATGCTACTCCTTCAGATTTTGT<br>TCGCGCTACTGCAACGATACCGATACAAGCCTCACTCCCTTTCGGATGGCTTATTGTTGGCGTTGCACTTCTTGCTGTTTTTCATAGCGCTTCCAAAATCATAACCCTC<br>AAAAAGAGATGGCAACTAGCACTCTCCAAGGGTGTTCACTTTGTTTGCAACTTGCTGTTGTTGTTTGTAACAGTTTACTCACACCTTTTGCTCGTTGCTGCTGGCCTTG<br>AAGCCCCTTTTCTCTATCTTTATGCTTTAGTCTACTTCTTGCAGAGTATAAACTTTGTAAGAATAATAATGAGGCTTTGGCTTTGCTGGAAATGCCGTTCCAAAAACCC<br>ATTACTTTATGATGCCAACTATTTCTTTGCTGGCATACTAATTGTTACGACTATTGTATACCTTACAATAGTGTAACTTCTTCAATTGTCATTACTTCAGGTGATGGC<br>ACAACAAGTCCTATTTCTGAACATGACTACCAGATTGGTGGTTATACTGAAAAATGGGAATCTGGAGTAAAAGACTGTGTTGTATTACACAGTTACTTCACTTCAGA<br>CTATTACCAGCTGTACTCAACTCAATTGAGTACAGACACTGGTGTTGAACATGTTACCTTCTTCATCTACAATAAAATTGTTGATGAGCCTGAAGAACATGTCCAAAT<br>TCACACAATCGACGGTTCATCCGGAGTTGTTAATCCAGTAATGGAACCAATTTATGATGAACCGACGACGACTACTAGCGTGCCTTTGTAAGCACAAGCTGATGAGT<br>ACCAATTTGTGTACTCATTCGTTTCGGAAGAGACAGGTACGTTAAACTCTCGTGTTAAAAATCTGAATTCTTCTAGAGTTCCTGATCTTCTGGTCTAAACGAACTAAA<br>TATTATATTAGTTTTTCTGTTTGGAACTTTAATTTTAGCCATGGCAGATTCCAACGGTACTATTACCGTTGAAGAGCTTAAAAAGCTCCTTGAACAATGGAACCTAGT<br>AATAGGTTTCCTATTCCTTACATGGATTTGTCTTCTACAATTTGCCTATGCCAACAGGAATAGGTTTTTGTATATAATTAAGTTAATTTTCCTCTGGCTGTTATGGCCA<br>GTAACTTTAGCTTGTTTTGTGCTTGCTGCTGTTTACAGAATAAATTGGATCACCGGTGGAATTGCTATCGCAATGGCTTGTCTTGTAGGCTTGATGTGGCTCAGCTACT<br>TCATTGCTTCTTTCAGACTGTTTGCGCGTACCCGTTCCATGTGGTCATTCAATCCAGAAACTAACATTCTTCTCAACGTGCCACTCCATGGCACTATTCTGACCAGACC<br>GCTTCTAGAAAGTGAACTCGTAATCGGAGCTGTGATCCTTCGTGGACATCTTCGTATTGCTGGACACCATCTAGGACGCTGTGACATCAAGGACCTGCCTAAAGAAA<br>TCACTGTTGCTACATCACGAACGCTTTCTTATTACAAATTGGGAGCTTCGCAGCGTGTAGCAGGTGACTCAGGTTTTGCTGCATACAGTCGCTACAGGATTGGCAACT<br>ATAAATTAAACACAGACCATTCCAGTAGCAGTGACAATATTGCTTTGCTTGTACAGTAAGTGACAACAGATGTTTCATCTCGTTGACTTTCAGGTTACTATAGCAGAG<br>ATATTACTAATTATTATGAGGACTTTTAAAGTTTCCATTTGGAATCTTGATTACATCATAAACCTCATAATTAAAAATTTATCTAAGTCACTAACTGAGAATAAATAT<br>TCTCAATTAGATGAAGAGCAACCAATGGAGATTGATTAAACGAACATGAAAATTATTCTTTTCTTGGCACTGATAACACTCGCTACTTGTGAGCTTTATCACTACCAA<br>GAGTGTGTTAGAGGTACAACA

Table S2. Characteristics of the gapmers.

Name	Sequence	Target	gRNA (nt numbers)	sgRNA (nt numbers)	Decrease of gRNA level (%)	Decrease of sgRNA level (%)	Decrease of the number of eGFP+ cells (%)	Decrease of eGFP intensity
G_N	$\mathrm{U}^{PS,L}\mathrm{U}^{PS,M}\mathrm{C}^{PS,L}A^{PS}A^{PS}C^{PS}G^{PS}A^{PS}G^{PS}A^{PS}C^{PS}\mathrm{U}^{PS,L}\mathrm{U}^{PS,M}\mathrm{C}^{PS,L}$	non-complimentary	-	-	-	-	-	
G1	$\mathrm{G}^{PS,L}\mathrm{A}^{PS,M}\mathrm{C}^{PS,L}G^{PS}T^{PS}T^{PS}G^{PS}T^{PS}T^{PS}T^{PS}G^{PS}\mathrm{A}^{PS,L}\mathrm{U}^{PS,M}\mathrm{C}^{PS,L}$	N	28380-28394	190-204	16	96	76	71
G2	$\mathrm{U}^{PS,L}\mathrm{G}^{PS,M}\mathrm{G}^{PS,L}A^{PS}G^{PS}A^{PS}A^{PS}A^{PS}T^{PS}C^{PS}A^{PS}\mathrm{U}^{PS,L}\mathrm{C}^{PS,M}\mathrm{C}^{PS,L}$	N	29473-29486	1283-1296	38	95	55	30
G6	$\mathrm{U}^{PS,L}\mathrm{U}^{PS,M}\mathrm{G}^{L,PS}C^{PS}C^{PS}G^{PS}A^{PS}G^{PS}G^{PS}C^{PS}T^{PS}\mathrm{U}^{PS,L}\mathrm{C}^{PS,M}\mathrm{U}^{PS,L}$	N	29040-29053	850-863	96	91	86	41
G7	$\mathrm{C}^{PS,L}\mathrm{U}^{PS,M}\mathrm{A}^{PS,L}C^{PS}T^{PS}G^{PS}C^{PS}T^{PS}G^{PS}C^{PS}C^{PS}\mathrm{U}^{PS,L}\mathrm{G}^{PS,M}\mathrm{G}^{PS,L}$	N	28868-28881	678-691	75	80	70	↑
G8	$\mathrm{U}^{PS,L}\mathrm{C}^{PS,M}\mathrm{A}^{PS,L}A^{PS}G^{PS}C^{PS}A^{PS}G^{PS}C^{PS}A^{PS}G^{PS}\mathrm{C}^{PS,L}\mathrm{A}^{PS,M}\mathrm{A}^{PS,L}$	N	28934-28947	744-757	91	93	93	51
G9	$\mathrm{A}^{PS,L}\mathrm{U}^{PS,M}\mathrm{C}^{PS,L}G^{PS}C^{PS}G^{PS}C^{PS}C^{PS}C^{PS}C^{PS}A^{PS}\mathrm{C}^{PS,L}\mathrm{U}^{PS,M}\mathrm{G}^{PS,L}$	N	28369-28382	179-192	52	99	98	60
N1	$\mathrm{C}^{PS,L}\mathrm{A}^{PS,M}\mathrm{U}^{PS,L}T^{PS}A^{PS}T^{PS}C^{PS}A^{PS}G^{PS}A^{PS}C^{PS}A^{PS}T^{PS}T^{PS}T^{PS}T^{PS}A^{PS}G^{PS}T^{PS}\mathrm{U}^{PS,L}\mathrm{U}^{PS,M}\mathrm{G}^{PS,L}$	N	28265-28286	75-96	52	98	63	70
N2	$\mathrm{C}^{PS,L}\mathrm{U}^{PS,M}\mathrm{U}^{PS,L}T^{PS}T^{PS}G^{PS}G^{PS}T^{PS}G^{PS}T^{PS}A^{PS}\mathrm{U}^{PS,L}\mathrm{U}^{PS,M}\mathrm{C}^{PS,L}$	N	28691-28703	501-513	58	98	64	61
N3	$\mathrm{U}^{PS,L}\mathrm{U}^{PS,M}\mathrm{C}^{PS,L}T^{PS}T^{PS}G^{PS}A^{PS}A^{PS}C^{PS}T^{PS}G^{PS}\mathrm{U}^{PS,L}\mathrm{U}^{PS,M}\mathrm{G}^{PS,L}$	N	28846-28859	656-669	41	98	71	61
N4	$\mathrm{G}^{PS,L}\mathrm{A}^{PS,M}\mathrm{U}^{PS,L}C^{PS}T^{PS}T^{PS}T^{PS}G^{PS}T^{PS}C^{PS}A^{PS}\mathrm{U}^{PS,L}\mathrm{C}^{PS,M}\mathrm{C}^{PS,L}$	N	29290-29303	1100-1113	97	98	87	69
N5	$\mathrm{U}^{PS,L}\mathrm{U}^{PS,M}\mathrm{G}^{PS,L}A^{PS}G^{PS}T^{PS}T^{PS}T^{PS}C^{PS}A^{PS}T^{PS}\mathrm{C}^{PS,L}\mathrm{A}^{PS,M}\mathrm{G}^{PS,L}$	N	29400-29413	1210-1223	38	92	-	33
3U1	$\mathrm{U}^{PS,L}\mathrm{A}^{PS,M}\mathrm{A}^{PS,L}C^{PS}T^{PS}A^{PS}C^{PS}A^{PS}T^{PS}C^{PS}T^{PS}A^{PS}C^{PS}T^{PS}T^{PS}G^{PS}\mathrm{U}^{PS,L}\mathrm{G}^{PS,M}\mathrm{C}^{PS,L}$	3' UTR	29639-29657	1482-1500	↑	(N;M)↑	↑	1
5U1	$\mathrm{G}^{PS,L}\mathrm{A}^{PS,M}\mathrm{A}^{PS,L}A^{PS}G^{PS}T^{PS}T^{PS}G^{PS}G^{PS}T^{PS}T^{PS}\mathrm{G}^{PS,L}\mathrm{G}^{PS,M}\mathrm{U}^{PS,L}$	5'UTR	31-44	31-44	56	95(N);94(M)	10	33
5U2	$\mathrm{C}^{PS,L}\mathrm{A}^{PS,M}\mathrm{C}^{PS,L}G^{PS}G^{PS}A^{PS}C^{PS}G^{PS}A^{PS}A^{PS}A^{PS}\mathrm{C}^{PS,L}\mathrm{C}^{PS,M}\mathrm{G}^{PS,L}$	5'UTR	197-210	-	95	92(N);94(M)	90	74
M1	$\mathrm{U}^{PS,L}\mathrm{U}^{PS,M}\mathrm{U}^{PS,L}A^{PS}G^{PS}T^{PS}T^{PS}C^{PS}G^{PS}T^{PS}T^{PS}T^{PS}A^{PS}\mathrm{G}^{PS,L}\mathrm{A}^{PS,M}\mathrm{G}^{PS,L}$	M	-	64-79	75	85	76	83

M2	$\mathrm{U^{PS,L}U^{PS,M}A^{PS,L}}C^{PS}T^{PS}A^{PS}G^{PS}G^{PS}T^{PS}T^{PS}C^{PS}C^{PS}A^{PS}\mathrm{U^{PS,L}U^{PS,M}G^{L,PS}}$	M	26577-26592	174-189	89	60	77	74
M3	$\mathrm{{}^{PP}A^{PS,L}}C^{PS}T^{PS}A^{PS}G^{PS}G^{PS}T^{PS}T^{PS}C^{PS}C^{PS}A^{\mathrm{PP}}\mathrm{G^{PS,L}}$	M	26577-26592	174-189	87	59	86	79
M4	$\mathrm{C^{PS,L}C^{PS,M}U^{PS,L}}G^{PS}T^{PS}T^{PS}G^{PS}G^{PS}C^{PS}A^{PS}T^{PS}A^{PS}\mathrm{G^{PS,}LG^{PS,M}C^{PS,L}}$	M	26634-26648	231-245	69	81	75	75
M5	$\mathrm{G^{PS,L}U^{PS,M}C^{PS,L}}T^{PS}G^{PS}A^{PS}A^{PS}A^{PS}G^{PS}A^{PS}A^{PS}G^{PS}C^{PS}\mathrm{A^{PS,L}A^{PS,M}U^{PS,L}}$	M	26811-26826	408-423	20	67	46	79
M6	$\mathrm{U^{PS,L}G^{PS,M}U^{PS,L}}G^{PS}T^{PS}T^{PS}T^{PS}A^{PS}A^{PS}T^{PS}T^{PS}T^{PS}\mathrm{A^{PS,L}U^{PS,M}A^{PS,L}}$	M	27132-27146	729-743	↑	25	15	61
M7	$\mathrm{G^{PS,L}G^{PS,M}U^{PS,L}}A^{PS}A^{PS}T^{PS}A^{PS}G^{PS}T^{PS}A^{PS}C^{PS}C^{PS}G^{PS}\mathrm{U^{PS,L}U^{PS,M}G^{PS,L}}$	M	26534-26549	131-146	92	97	90	82
Modifications of nucleotides (N): N^{PS}-phosphorothioate, N^{M}-2′O-methylated RNA, N^{L}-locked nucleic acid, N^{PP}-pseudouridine.								

Table S3. Sequence conservation of the gapmers G6, G8, N4, M7, and 5U2 target sites across major SARS-CoV-2 variants. SARS-CoV-2 sequences were obtained from the Nextstrain database.

**Name**					**G6**	**G8**	**N4**	**M7**	**5U2**
**Sequence**					TTGCCGAGGCTTCT	TCAAGCAGCAGCAA	GATCTTTGTCATCC	GGTAATAGTACCGTTG	CACGGACGAAACCG
**Genome**	**Sample year**	**Pango lineage (Nextclade)**	**Clade**	**WHO variant**	Mismatches<br>Total alignment	Mismatches<br>Total alignment	Mismatches<br>Total alignment	Mismatches<br>Total alignment	Mismatches<br>Total alignment
MN908947	2019	B	19A	basal early SARS-CoV-2 lineage	0/14<br>100%	0/14<br>100%	0/14<br>100%	0/16<br>100%	0/14<br>100%
MZ544367	2020	B.1.1	20B	early lineage	0/14<br>100%	0/14<br>100%	0/14<br>100%	0/16<br>100%	0/14<br>100%
OV264082	2021	B.1.1.7	20I	Alpha	0/14<br>100%	0/14<br>100%	0/14<br>100%	0/16<br>100%	0/14<br>100%
OU205208	2021	B.1.617.2	21A	Delta	0/14<br>100%	0/14<br>100%	0/14<br>100%	0/16<br>100%	1/13<br>92%
OQ521295	2021	AY.4	21J	Delta	0/14<br>100%	0/14<br>100%	0/14<br>100%	0/16<br>100%	1/13<br>92%
OQ586645	2022	BA.2.2.1	21L	Omicron	0/14<br>100%	0/14<br>100%	0/14<br>100%	0/16<br>100%	0/14<br>100%
OY265039	2022	BA.5.2	22B	Omicron	0/14<br>100%	0/14<br>100%	0/14<br>100%	0/16<br>100%	0/14<br>100%
PP330140	2023	JN.1.44	24A	Omicron	0/14<br>100%	0/14<br>100%	0/14<br>100%	0/16<br>100%	0/14<br>100%
OZ087455	2024	KP.2	24B	Omicron	0/14<br>100%	0/14<br>100%	0/14<br>100%	0/16<br>100%	0/14<br>100%
PX399068	2025	NB.1.8.1	25B	Omicron	0/14<br>100%	0/14<br>100%	0/14<br>100%	0/16<br>100%	0/14<br>100%
PZ623340	2026	XGF.1.1	25C	Omicron	0/14<br>100%	0/14<br>100%	0/14<br>100%	0/16<br>100%	0/14<br>100%

Table S4. Oligonucleotides and templates used for assembly of pBAC-SARS-CoV-2_ *S/eGFP_ΔE* replicon

Primer name	Primer sequence	Amplified sequence	Template
F.1.1.F	ACGCGTGGGACCCGCGATCGCGGCGCGCCAAGCTTGGCGTAATCATG GTCATAGCTG	pUC19-MST	pUC19
F.1.1.R	ACGCGTTAATTAACGCCGGCGCGTACGAGCTCGTCGACGGCGCCTGA TGCGGTATTTTCTC	pUC19-MST	pUC19
F.1.2.VF	GTTTAGTGAACCGTCGTACGCGCCGGCG	pUC19-MST	pUC19-(F1:MCS)
F.1.2.VR	TGTCAACGTCGACGGCGCCTGATG	pUC19-MST	pUC19-(F1:MCS)
F.1.2.IF	CCGTCGACGTTGACATTGATTATTGACTAGTTATTAATAGTAATCAAT TACGG	CMV	pcDNA3.1 CMV mCherry-eGFP BGH
F.1.2.IR	GACGGTTCACTAAACGAGCTCTGCTTATATAGACCTCCC	CMV	pcDNA3.1 CMV mCherry-eGFP BGH
F.1.3.VF	ACGTGCTCGTACGCGCCGGC	pUC19-CMV-MST	pUC19-(F1:CMV-MCS)
F.1.3.VR	CCTTTAATACGGTTCACTAAACGAGCTCTGCT	pUC19-CMV-MST	pUC19-(F1:CMV-MCS)
F.1.3.IF	GAACCGTATTAAAGGTTTATACCTTCCCAGGTAACAAACC	5'UTR-*ORF1*[86]	cDNA SARS-CoV-2 strain Slovakia/SK-BMC5/2020
F.1.3.IR	CGCGTACGAGCACGTCGC	5'UTR-*ORF1*[86]	cDNA SARS-CoV-2 strain Slovakia/SK-BMC5/2020
F.2F	CGTGCTCGTACGTGGCTTT	F2	cDNA SARS-CoV-2 strain Slovakia/SK-BMC5/2020
F.2R	AAGGCGCCGGCGACACCTCCTCCATGTTTAAGGTAAACA	F2	cDNA SARS-CoV-2 strain Slovakia/SK-BMC5/2020
F.3F	GTGTCGCCGGCGCCTTAAATAAGGCTACTAACAATGCCA	F3	cDNA SARS-CoV-2 strain Slovakia/SK-BMC5/2020
F.3R	GTAACTTTAATTAACTGCTTCAACCAATTATTAACAATTTTACCAC	F3	cDNA SARS-CoV-2 strain Slovakia/SK-BMC5/2020
F.4F	GAAGCAGTTAATTAAAGTTACACTTGTGTTCCTT	F4	cDNA SARS-CoV-2 strain Slovakia/SK-BMC5/2020
F.4R	GCGTATACGCGTAATATATCTGGGTTTTCT	F4	cDNA SARS-CoV-2 strain Slovakia/SK-BMC5/2020
F.5F	ATATTACGCGTATACGCCAACTTAGGTG	F5	cDNA SARS-CoV-2 strain Slovakia/SK-BMC5/2020

F.5R	TCTAGGGTCCCCTTAGTTAGCAATGTGCGTGG	F5	cDNA SARS-CoV-2 strain Slovakia/SK-BMC5/2020
F.6F	TAAGGGGACCCTAGAACCAGAATATTTCAATTCAGTGTGTAGACT	F6	cDNA SARS-CoV-2 strain Slovakia/SK-BMC5/2020
F.6R	CCATTGCGATCGCTAGTGGCAATAAAACAAGAAAAACAAACATTGTTC	F6	cDNA SARS-CoV-2 strain Slovakia/SK-BMC5/2020
F.7.2.1F	GGAGAATGACAAAAAAAAAAAAAAAAAAAAAAAAAAAAAAAAAAGGGTCGGCATGGC	F7.2poly(A)-pET-21(+)	F7.2-pET-21(+)
F.7.2.1R	TTTTTGTCATTCTCCTAAGAAGCTATTAAAATCACATGGG	F7.2poly(A)-pET-21(+)	F7.2-pET-21(+)
F.7.I1F	TCTAGGATCCAGAAGGCTATGCCTTCGAACAT	*ORF1*-1148-3'	cDNA SARS-CoV-2 strain Slovakia/SK-BMC5/2020
F.7.I1R	TTGATTTGACCTTCTTTTAAAGACATAACAGCAGTAC	*ORF1*-1148-3'	cDNA SARS-CoV-2 strain Slovakia/SK-BMC5/2020
F.7.I2F	ATGTCTTTAAAAGAAGGTCAAATCAATGATATGAT	F7.1, F7	pTwist-Amp-MC-F7.2
F.7.I2R	GTTTAATCAATCTCCATTGGTTGCT	F7.1	pTwist-Amp-MC-F7.2
F.7.VF	AGCAACCAATGGAGATTGATTAAACGAACA	F7.2poly(A)-pET-21(+)	F7.2poly(A)-pET-21(+)
F.7.VR	GCATAGCCTTCTGGATCCTAGAGGGGAATTGTTATC	F7.2poly(A)-pET-21(+)	F7.2poly(A)-pET-21(+)
F.7R	GAAACAGCTATGACCATGATTACGCC	F7	F7-pET-21(+)

Table S5. Primers used for reverse transcription and qPCR assay

Primers name	Primer sequence
**Reverse Transcrition**	
Anti-Leader-RT	TTTATACCTTCCCAGGTAAC
GAPDEH-RT	CTGTTGCTGTAGCCAAATTCG
3UTR-RT	CTCCTAAGAAGCTATTAAAATCAC
**qPCR**	
Leader_For	CCAACCAACTTTCGATCTCTTGTAGATCTG
qRNA_Rev	CAGGCAAACTGAGTTGGACGTG
N_Rev	TTGGGTAAACCTTGGGGCCG
M_Rev	CCTATTACTAGGTTCCATTGTTCAAGGAGC
GAPDEH_For	GAAGGTGAAGGTCGGAGTC
GAPDEH_Rev	GAAGATGGTGATGGGGCTTC
MX1_For	AGGACCATCGGAATCTTGAC
MX1_Rev	TCAGGTGGAACACGAGGTTC
