# Supplementary material for: Bridging the antiviral gap: A BAC-based non-infectious SARS-CoV-2 replicon reveals potent antisense gapmers
Source: Mol Ther Nucleic Acids. 2026 Sep 15;37(4):103098. doi: 10.1016/j.omtn.2026.103098 (PMC13656313; doi:10.1016/j.omtn.2026.103098)
Supplement: Supplementary file 2 [file mmc2.pdf]

Molecular Therapy
Nucleic Acids
Original Article

Bridging the antiviral gap: A BAC-based non-infectious SARS-CoV-2 replicon reveals potent antisense gapmers

Agnieszka Ruszkowska,[1,3] Agnieszka Baliga-Gil,[1,3] Julita Piasecka,[1,2] Maria Ciechanowska,[1] Ryszard Kierzek,[1] and Elzbieta Kierzek[1]

[1]Institute of Bioorganic Chemistry, Polish Academy of Sciences, Noskowskiego 12/14, 61-704 Poznan, Poland

To facilitate studies of SARS-CoV-2 replication and antiviral drug discovery, we developed a non-infectious SARS-CoV-2 replicon, pBAC-SARS-CoV-2_*S/eGFP*_ΔE, based on a bacterial artificial chromosome vector. The viral genome was modified to ensure non-infectivity while allowing replication monitoring: the spike (S) protein and envelope (E) protein open reading frames (*ORF*) were deleted, and *ORF S* was replaced with an enhanced *GFP* (*eGFP*) reporter. Upon transfection, the pBAC-SARS-CoV-2_*S/eGFP*_Δ*E* was replication-competent in HEK293T cells and was inhibited by remdesivir, validating the replicon utility for studying SARS-CoV-2 replication and antiviral screening. RNA structures regulate the SARS-CoV-2 replication cycle by controlling genome stability, expression, and interactions with viral and host proteins. Using the pBAC-SARS-CoV-2_*S/eGFP*_Δ*E*, we evaluated antisense gapmers targeting RNA structural motifs within the untranslated regions (UTRs) and nucleocapsid (*N*) and membrane (*M*) genes. Screening identified five potent gapmers: one targeting the 5′ UTR, three targeting the *N* gene, and one targeting the *M* gene. The *M*-directed gapmer was the most active, with an EC_{50} of 6.3 nM, highlighting the *M* coding region as a promising antisense target. Four of the identified gapmers, including the lead, recognize accessible motifs present in both genomic and subgenomic RNAs, suggesting that dual targeting may be an effective antiviral strategy.

INTRODUCTION

In recent years, coronaviruses have emerged as a significant epidemiological concern to human population and environment from the so-called "one health" perspective.[1] The SARS-CoV-2 pandemics clearly highlighted the need to deepen our understanding of coronavirus infection mechanisms to improve the development of effective vaccines and therapeutics. During the early phases of the pandemic, work with SARS-CoV-2 commonly required BSL-3 containment because of its pandemic potential, transmissibility, and the limited availability of vaccines and antiviral therapies at that time.[2] More recent CDC guidance supports a risk-based approach to SARS-CoV-2 research, with BSL-2 as the minimum containment level and measures adjusted according to the specific activity and local assessment.[3,4] Nevertheless, non-infectious replicons provide a safer and more accessible alternative to study viral biology and inhibition. These versatile systems support precise genetic manipulations under controlled conditions, facilitate investigation of the viral replication cycle, and advance vaccine and antiviral drugs by enabling high-throughput screening.

A viral replicon is a genetic construct containing sequences necessary for viral genome replication, including protein-coding genes and regulatory elements. Several approaches have already been used to generate replicons of emerging coronaviruses such as SARS-CoV, MERS-CoV, and SARS-CoV-2.[2] Replicons are generally categorized by their genetic composition, method of delivery, and ability to produce viral particles. Structurally, they can be either RNA-based or DNA-based constructs. A range of strategies can be employed to generate them, e.g., bacterial artificial chromosome (BAC) vectors,[5–10] vaccinia virus vectors,[11,12] *in vitro* ligation of cDNA fragments,[13–16] yeast-based transformation-associated recombination (TAR) cloning,[17–19] PCR-based circular polymerase extension reaction (CPER)[20–22] or a combination of the above-mentioned techniques.[23] Among these, the BAC system is the most widely used due to its stability, large cloning capacity, ease of manipulation, possibility of production in large quantities, and compatibility with multiple transfection approaches.[6] The application of BACs has enabled the successful rescue of SARS-CoV, MERS-CoV, and SARS-CoV-2 replicons, yielding recombinant viruses with growth characteristics and plaque morphologies comparable to those of natural isolates.[2]

Received 16 December 2025; accepted 14 September 2026;
https://doi.org/10.1016/j.omtn.2026.103098.

[2]Present address: Department of Bioprocess Engineering, Max Planck Institute for Dynamics of Complex Technical Systems, Sandtorstrasse 1, 39106 Magdeburg, Germany

[3]These authors contributed equally

Correspondence: Agnieszka Ruszkowska, Institute of Bioorganic Chemistry, Polish Academy of Sciences, Noskowskiego 12/14, 61-704 Poznan, Poland.
E-mail: aruszkowska@ibch.poznan.pl
Correspondence: Elzbieta Kierzek, Institute of Bioorganic Chemistry, Polish Academy of Sciences, Noskowskiego 12/14, 61-704 Poznan, Poland.
E-mail: elzbieta.kierzek@ibch.poznan.pl

© 2026 The Authors.
Published by Elsevier Inc. on behalf of The American Society of Gene and Cell Therapy.
This is an open access article under the CC BY license (http://creativecommons.org/licenses/by/4.0/).

Non-infectious replicons lack the genes required for virion assembly, release, and/or cell entry. In the case of SARS-CoV-2, these replicons often feature deletions of genes encoding structural proteins and insertion of reporter genes.[6,8,19] In consequence, upon transfection, fully infectious viral particles cannot be produced. Therefore, the design requires a detailed understanding of the viral genome and the genes involved in pathogenesis. Non-infectious replicons may carry a deletion of a single gene that is crucial for virulence, such as the spike (*S*) gene. Alternatively, they may carry the loss of multiple genes, such as the *S*, membrane (*M*), and envelope (*E*) genes.[6,9,17,18,23–25] Further deletion of the genes is often used to study viral protein functions and host-pathogen interactions. The aforementioned reverse genetics approaches also enable the addition of reporter genes such as luciferase or green fluorescence protein (GFP), which expression is induced in a replication-dependent manner.[12,17,19,24,25] These reporter genes are further utilized for both rapid antiviral drug screening and viral replication cycle studies.

RNA structural motifs play a critical role in the replication cycle of SARS-CoV-2.[1] Coronavirus *cis*-acting secondary RNA structures, primarily found in the untranslated regions (UTRs), serve as essential regulatory elements for viral RNA synthesis, replication, and translation.[26–28] The 5′ and 3’ UTRs harbor conserved stem-loop structures and pseudoknots that mediate the recruitment and positioning of the viral replicase complex, thus orchestrating discontinuous transcription of subgenomic RNAs (sgRNAs) critical for viral protein production.[29,30] Therefore, conserved RNA motifs represent highly promising targets for antiviral strategies.[31] Given their functional importance, these regions commonly exhibit reduced mutation rates or accumulate mutations that preserve their secondary structure, consistent with selective pressure to maintain RNA structure-function relationships.[32–34] For the development of RNA-targeting therapeutics, it is also essential to select gene targets that are crucial to the viral replication cycle. The nucleocapsid (N) and membrane (M) proteins possess the necessary characteristics to be considered as potent candidates for further investigation. Among structural proteins, the M and N proteins are highly conserved and abundantly expressed.[35,36] They perform diverse functions, including viral genomic RNA (gRNA) packaging, virion assembly, and modulation of the host immune response. Moreover, the N protein acts as an RNA chaperone that facilitates discontinuous transcription of sgRNAs via the TRS-mediated template-switching mechanism.[37–41] Consequently, the N protein plays a pivotal role in the coronavirus replication cycle.

Here, we report the construction of a SARS-CoV-2 replicon, pBAC-SARS-CoV-2_*S/eGFP*_Δ*E*, using a BAC vector (Figure 1). This replicon features simultaneous deletion of the *S* and *E* genes, abolishing infectious-particle formation and generating a safe, assembly-deficient system suitable for BSL-2 work. Replacement of *S* with *eGFP* enables real-time fluorescence-based monitoring of replicon activity. In contrast to replicons carrying broader multi-gene deletions, retention of the *M* gene preserves expression of the most abundant structural protein and maintains *M*-associated RNA, the latter being an important element of investigation in this study. Using this system, we performed antiviral screening of gapmers designed to target conserved RNA structural motifs of SARS-CoV-2. This approach yielded a panel of gapmers directed against the UTRs, *N* gene, and *M* gene, which demonstrated a pronounced antiviral effect.

RESULTS

Construction of pBAC-SARS-CoV-2_*S/eGFP*_Δ*E* replicon

In this study, we used seven PCR-amplified DNA fragments (F1-F7) and the pBeloBAC11 vector[42] (F8) to create a non-infectious clone of SARS-CoV-2 (Figures S1 and 1B–1D). The pBAC-SARS-CoV-2_ *S/eGFP_ΔE* replicon was assembled stepwise by restriction enzyme cloning, using the following restriction sites: *Kas*I, *Bsi*WI, *Mre*I, *Pac*I, *Mlu*I, *Kfl*I, *Asi*SI, *Asc*I, and *Hind*III. Among these, three unique restriction sites, *Bsi*WI, *Mlu*I, and *Pac*I, were naturally present in the SARS-CoV-2 genome, while two additional sites, *Mre*I and *Kfl*I, were introduced within *ORF1ab* by synonymous mutations ([3478T/C, 3481A/C, 3484A/C]; [17481C/G, 17484A/C]). Furthermore, a single synonymous substitution at 26840G/C in *ORF M* eliminated secondary recognition sites for *Bsi*WI and *Mlu*I. At the early stage of assembly, *Kas*I and *Hind*III were used to insert F1 into pBeloBAC11 (Figure 1D). In the final construct, *Kas*I and *Hind*III recognition sites occur multiple times (three and 18, respectively) and are therefore not suitable for future modification. The unique restriction sites present in the final construct, *Sgr*DI, *Bsi*WI, *Mre*I, *Pac*I, *Mlu*I, *Kfl*I, *Asi*SI, and *Asc*I, are intended for potential future modifications of pBAC-SARS-CoV-2_ *S/eGFP_ΔE* (Figures 1C and 1D).

Two of the eight DNA fragments, F1 and F7 (Table S1), were first assembled in intermediate plasmids—pUC19 (yielding pUC19-F1) and pET-21(+) (yielding pET-21(+)-F7), using PIPE, Gibson Assembly, and restriction enzyme cloning. F1 consisted of CMVp, a short SARS-CoV-2 sequence containing the 5' UTR and 86 nt of *ORF1*, together with MCS incorporating all restriction sites described earlier (Figure 1C). F7 comprised *Asi*SI site, the genomic region spanning from the *ORF S* to the 3' UTR, followed by a polyadenylation tract (33 nt), HDVr, and BGHpA and *Asc*I site (Table S1). To ensure non-infectivity of the replicon, the *S* and *E* genes, within F7, were deleted as previously described for SARS-CoV replicons construction.[25,43] The spike transcription-regulating sequence (TRS) was retained to drive expression of the *eGFP* reporter gene (*S/eGFP*) (Figure 1C).

Individual DNA fragments (F1-F7) were subsequently inserted into the BAC backbone (F8) in a stepwise manner. F1 was PCR-amplified from the pUC19-F1 and cloned into pBeloBAC11, providing the MCS for all subsequent steps. Fragments F2-F6, covering *ORF1ab* (sequence of replicase complex) were amplified from the cDNA of the SARS-CoV-2 strain Slovakia/SK-BMC5/2020 (Figure S1) and inserted sequentially into the BAC plasmid. Finally, F7 was amplified from the pET-21(+)-F7 vector and integrated into the intermediate BAC vector to obtain the pBAC-SARS-CoV-2_ *S/eGFP_ΔE*. In the

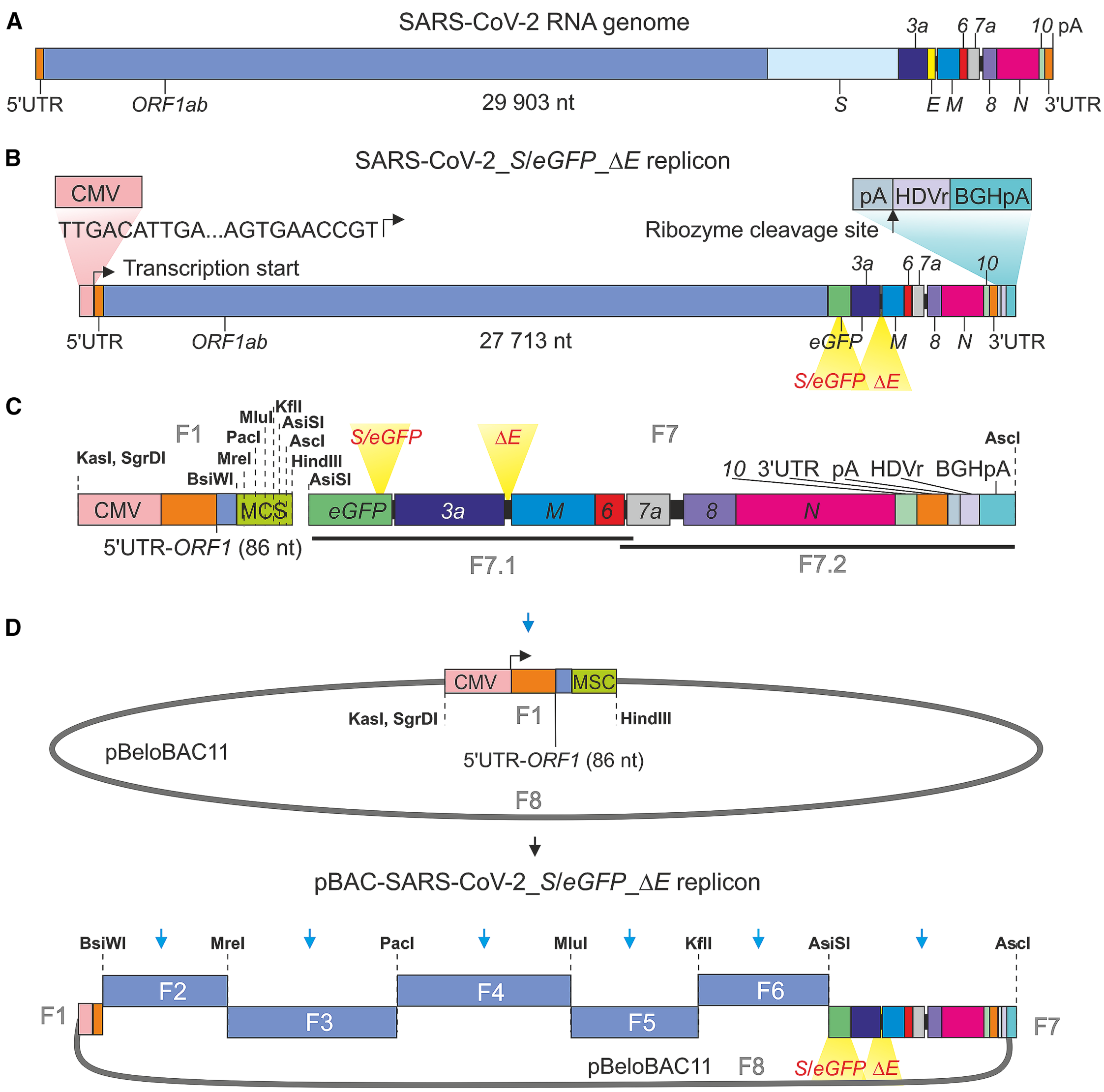

Figure 1. Construction of pBAC-SARS-CoV-2_*S/eGFP*_Δ*E* replicon

(A) Schematic organization of the SARS-CoV-2 genome. Open reading frames for viral proteins are indicated as: *ORF1ab, S, 3a, E, M, 6, 7a, 8, N, 10*. The 5′ and 3′ untranslated regions (UTRs) are shown in orange, and the 33-adenosine poly(A) tract is labeled "pA." (B) Transcription unit of the pBAC-SARS-CoV-2_*S/eGFP*_Δ*E* replicon. *S/eGFP* gene substitution (*S/eGFP*) and *E* deletion are highlighted in yellow. Other sequences are marked as in (A) and as follows: CMV, cytomegalovirus enhancer/promoter; HDVr, hepatitis delta virus ribozyme; BGHpA, bovine growth hormone termination and polyadenylation signal. (C) Characteristics of DNA fragments, F1 and F7, assembled in pUC19 and pET-21(+) vectors, respectively. DNA sequences are color marked as in (A) and (B). The multiple cloning site (MCS) is indicated. (D) Stepwise restriction-enzyme-based assembly of fragments F1-F7 into the pBeloBAC11 backbone (F8). Blue arrows denote sequential cloning steps. The *S/eGFP* insertion and *E*-gene deletion are shown as in (B). Sequence annotations follow the color scheme used in (A)–(C).

completed replicon, the modified SARS-CoV-2 genome is flanked by CMVp at the 5' end and by HDVr followed by BGHpA at the 3' end. CMVp and BGHpA function as transcriptional regulatory elements, enabling the nuclear transcription of the full-length viral genome.[11] The HDVr mediates precise cleavage downstream of the poly(A) tract in the 3' UTR, thereby ensuring the generation of homogeneous 3' termini.[44] Collectively, this design couples transcription with ribozyme-mediated self-processing to produce viral RNAs with authentic SARS-CoV-2 genomic 5' and 3' ends (Figure 1B).

Application of SARS-CoV-2 replicon in drug discovery

Expression of pBAC-SARS-CoV-2_S/eGFP_ΔE in cells and its sensitivity to known SARS-CoV-2 inhibitor

To confirm pBAC-SARS-CoV-2_*S/eGFP*_Δ*E* replication in mammalian cells, we transfected this construct into HEK293T and monitored reporter activity using fluorescence microscopy and reverse transcription followed by qPCR (RT-qPCR). 48 h after lipofectamine transfection, we detected eGFP fluorescence (Figure 2A). As the spike transcription-regulating sequence (TRS) was used to drive expression

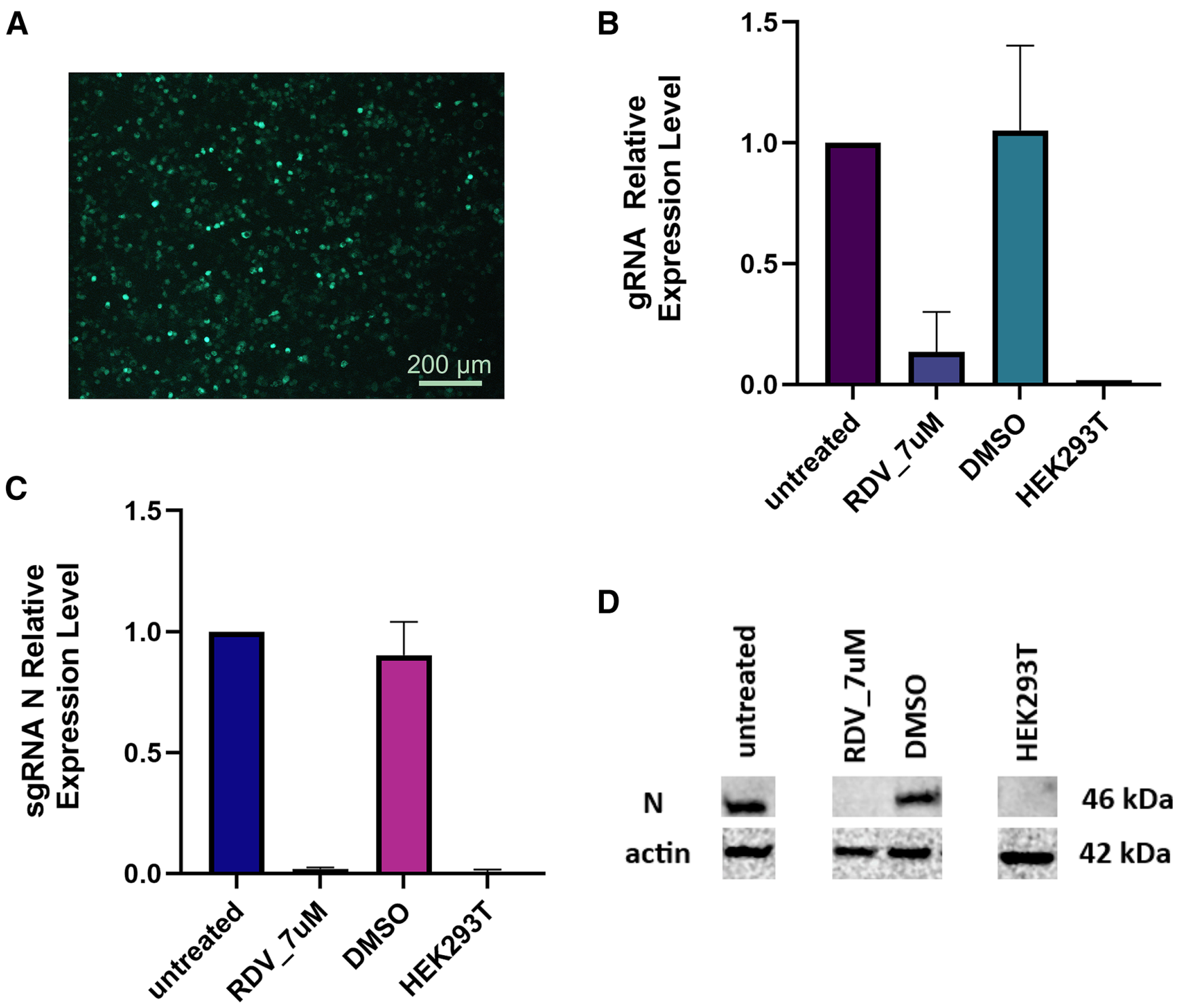

Figure 2. Expression of pBAC-SARS-CoV-2_*S/eGFP_ΔE* in cells and its sensitivity to known SARS-CoV-2 inhibitor

(A) Fluorescence microscopy image of eGFP expression in HEK293T cells 48 h after transfection with pBAC-SARS-CoV-2_*S/eGFP_ΔE*. (B and C) Effect of remdesivir (RDV) on the levels of replicon genomic RNA (gRNA; B) and subgenomic RNA encoding the N protein (sgRNA *N*; C), as determined by RT-qPCR using *GAPDH* as the internal reference. Bars represent the mean ± SD from two biological replicates, each with two technical replicates. Sample labels are defined as follows: untreated, HEK293T cells transfected with the replicon alone; RDV_7 μM, HEK293T cells transfected with the replicon and treated with 7 μM RDV; DMSO, HEK293T cells transfected with the replicon and treated with 0.1% DMSO; HEK293T, untransfected cells used as a negative control. (D) Western blot analysis of N protein expression in HEK293T cells transfected with pBAC-SARS-CoV-2_*S/eGFP_ΔE* and treated with RDV.

of the reporter gene (*S/eGFP*), the observed eGFP activity allowed to confirm viral replication, transcription, and translation. Intensity of eGFP reflected the overall extent of replicon activity. We therefore additionally quantified levels of gRNA and sgRNA encoding the N protein (sgRNA *N*) by RT-qPCR and assessed N protein expression by western blotting, and confirmed efficient production of viral components (Figures 2B–2D and S2). To assess whether the pBAC-SARS-CoV-2_ *S/eGFP_Δ E* system generated infectious viral particles, we performed plaque assay in Vero E6 cells using supernatants collected from replicon-transfected HEK293T cells. As expected, no plaques were detected in inoculated target cells, indicating that the system does not produce infectious viral progeny. If viral-like particles are formed, they remain non-infectious and are unable to establish productive infection in susceptible cells.

To verify the usability of pBAC-SARS-CoV-2_ *S/eGFP_Δ E* in antiviral screening, we evaluated the sensitivity of the BAC-driven system to remdesivir, the well-known inhibitor of the SARS-CoV-2 RdRp.[45] The EC_{50} of remdesivir against SARS-CoV-2 is highly dependent on the experimental system used, including the cell line, quantification method, and whether antiviral activity is measured using infectious virus or a non-infectious replicon system. As reported previously, remdesivir is an effective inhibitor of infectious SARS-CoV-2 replication in human lung cells and primary human airway epithelial cultures, with an EC_{50} of approximately 0.01 μM, whereas substantially weaker activity has been detected in Vero E6 cells (EC_{50} = 1.65 μM).[45] Similarly, the antiviral activity of remdesivir varies considerably among the SARS-CoV-2 replicon systems. For RNA replicons transcribed *in vitro* from BAC-based constructs, EC_{50} range from 0.016 to 2.24 μM, with the highest activity observed in HEK293T and weakest activity in Vero E6 cells.[8,15] Furthermore, the replication of the pYES1L-derived, CMV-driven SARS-CoV-2 replicon in HEK293T cells was substantially less sensitive to remdesivir, with EC_{50} of 6.8 μM.[19] However, Tanaka et al. demonstrated that 0.5 μM remdesivir significantly inhibited replication of a BAC-based, CMV-driven SARS-CoV-2 replicon in HEK293T cells.[23] Taking these data into account, we decided to apply 7 μM remdesivir in pilot experiments to achieve clear inhibition of the replicon replication. To measure inhibitory effects on the viral replication cycle, we applied RT-qPCR, assessing the levels of gRNA and sgRNA *N* and Western Blot, investigating the abundance of N protein (Figures 2B–2D). Treatment with 7 μM remdesivir reduced gRNA levels by up to 87% and sgRNA *N* levels by up to 98% (Figures 2B and 2C). Consistently, N protein levels were strongly reduced in the presence of remdesivir, approaching complete suppression (Figure 2D). Next, we assessed the dose-dependent activity of remdesivir against pBAC-SARS-CoV-2_*S/eGFP*_Δ*E* in HEK293T cells by RT-qPCR and determined an EC_{50} of 24.4 nM (Figure S3). This result indicates that pBAC-SARS-CoV-2_*S/eGFP*_Δ*E* provides an efficient and sensitive tool for identifying inhibitors active against SARS-CoV-2.

Selection of SARS-CoV-2 RNA structural targets

pBAC-SARS-CoV-2_ *S/eGFP*_ Δ*E* replicon was used to study antiviral activity of gapmers targeting RNA motifs within 3′ and 5′ UTRs and *N*, *M* coding sequence. Gapmer target regions were selected based on the well-characterized RNA structure of SARS-CoV-2 sgRNA[46,47] and gRNA.[29,48,49] We implemented multiple design strategies: gapmers were positioned either to disrupt defined

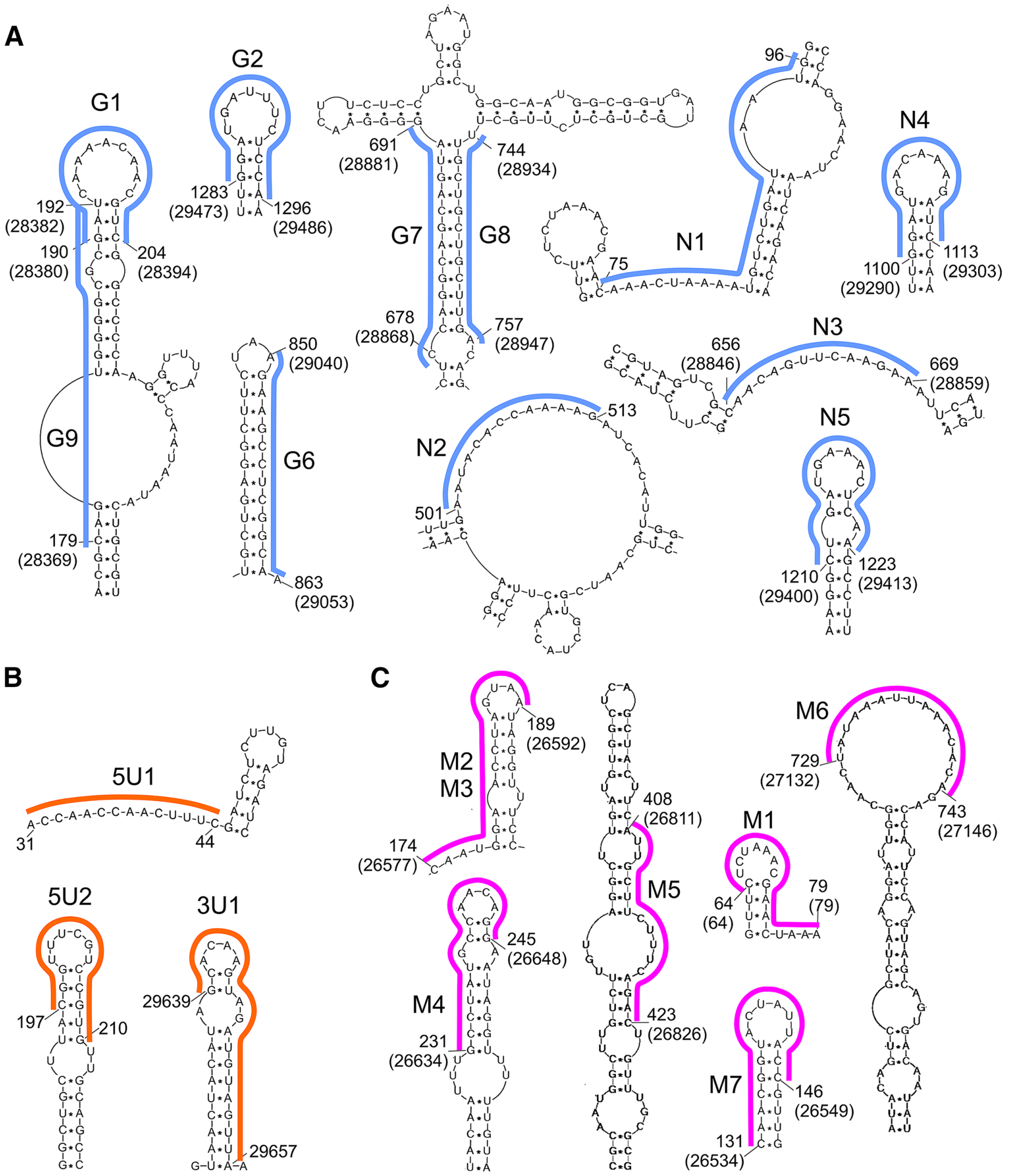

Figure 3. RNA structural motifs within SARS-CoV-2 sgRNA *N*, sgRNA *M*, and gRNA targeted by gapmers

(A) Structural motifs present in sgRNA *N*.[46] The regions targeted by gapmers are highlighted in blue. Numbering is shown as follows: region of sgRNA (sequence in gRNA). If the motif is not preserved in gRNA, the numbering in parentheses is omitted. (B) RNA structures located in the UTRs of gRNA.[48,49] Gapmer-targeted regions are indicated in orange. (C) Structural motifs identified in sgRNA *M*.[47] Gapmer target sites in sgRNA *M* are highlighted in magenta. Numbering is as described in (A).

hairpin motifs or structural domains, or to bind within predicted single-stranded, accessible regions. The selected sites reside in conserved structural elements[46,47] (Figure 3).

Screening for inhibitors of SARS-CoV-2 replication

In our previous work, we identified a set of oligonucleotides active against sgRNA *N* of SARS-CoV-2 using a eukaryotic expression system encoding this RNA (V-sgRNA_N-eGFP-1[46]). Although informative, that system was limited to assessing direct effects on the target RNA and did not capture consequences at the level of viral replication. To overcome this limitation, we employed a SARS-CoV-2 replicon system (pBAC-SARS-CoV-2_*S/eGFP*_Δ*E*), which provides a more physiologically relevant context. Using this approach, we evaluated the inhibitory activity of six selected gapmers targeting the *N* genomic region and sgRNA *N*. Gapmer antisense oligonucleotides which consist of a central DNA region flanked by chemically modified nucleotides (see materials and methods – oligonucleotides), were designed to induce target RNA degradation via RNase H1 following formation of a DNA/RNA heteroduplex. An additional gapmer, G_N, not complementary to SARS-CoV-2 RNA sequence, was used as a negative control. Each gapmer was tested individually in HEK293T cells transfected with pBAC-SARS-CoV-2_*S/eGFP*_Δ*E*. Inhibitory effects were quantified by flow cytometry by measuring the percentage of eGFP-positive cells among viable cells (Figure S4). The data were normalized to HEK293T cells transfected with the replicon alone, which served as the 100% positive control (Figure 4; Table S2). In our earlier study, gapmers G1, G2, G6, G7, G8, and G9 reduced the eGFP-positive cells by 58%–81%.[46] In the current experiments, all six gapmers retained detectable inhibitory activity, although with varying degrees of efficacy (Figures 4A; Table S2). Specifically, G2 and G7 maintained their inhibitory effect at levels similar to previously observed—55% vs. 58% and 70% vs. 74%. Gapmers G1 and G6 showed slightly increased inhibitory activity—76% vs. 67% and 86% vs. 74%, respectively. G8 and G9 reduced the number of eGFP-positive cells by 93% and 98% in the system involving pBAC-SARS-CoV-2_*S/eGFP_ΔE* construct, whereas in the V-sgRNA_N-eGFP-1 system, the reduction reached only 74% and 81%, respectively. Overall, gapmers G1-G9 targeting the *N* coding region exhibited inhibitory effects in the pBAC-SARS-CoV-2_*S/eGFP*_Δ*E* system comparable to those observed in our previous study[46] (Figures 4A; Table S2), thereby supporting their antiviral potential. The slight discrepancies in inhibitory activity may be explained by (1) differences in the experimental setup, as in our previous work the *N-eGFP* transcript itself was the direct target of the gapmers, whereas in the current replicon system eGFP is translated from the TRS of Δ*S*, and *eGFP* gene is not directly targeted by the oligonucleotides; and/or (2) the use of a biologically more relevant model, in which the effects of gapmers are reflected not solely at the level of sgRNA *N* abundance but also through their impact on viral molecules dynamics, including the role of *N* in genome replication and sgRNA transcription.

To evaluate the direct effects of G1, G2, G6, G7, G8, and G9 gapmers on viral RNA, we quantified gRNA and sgRNA *N* levels using

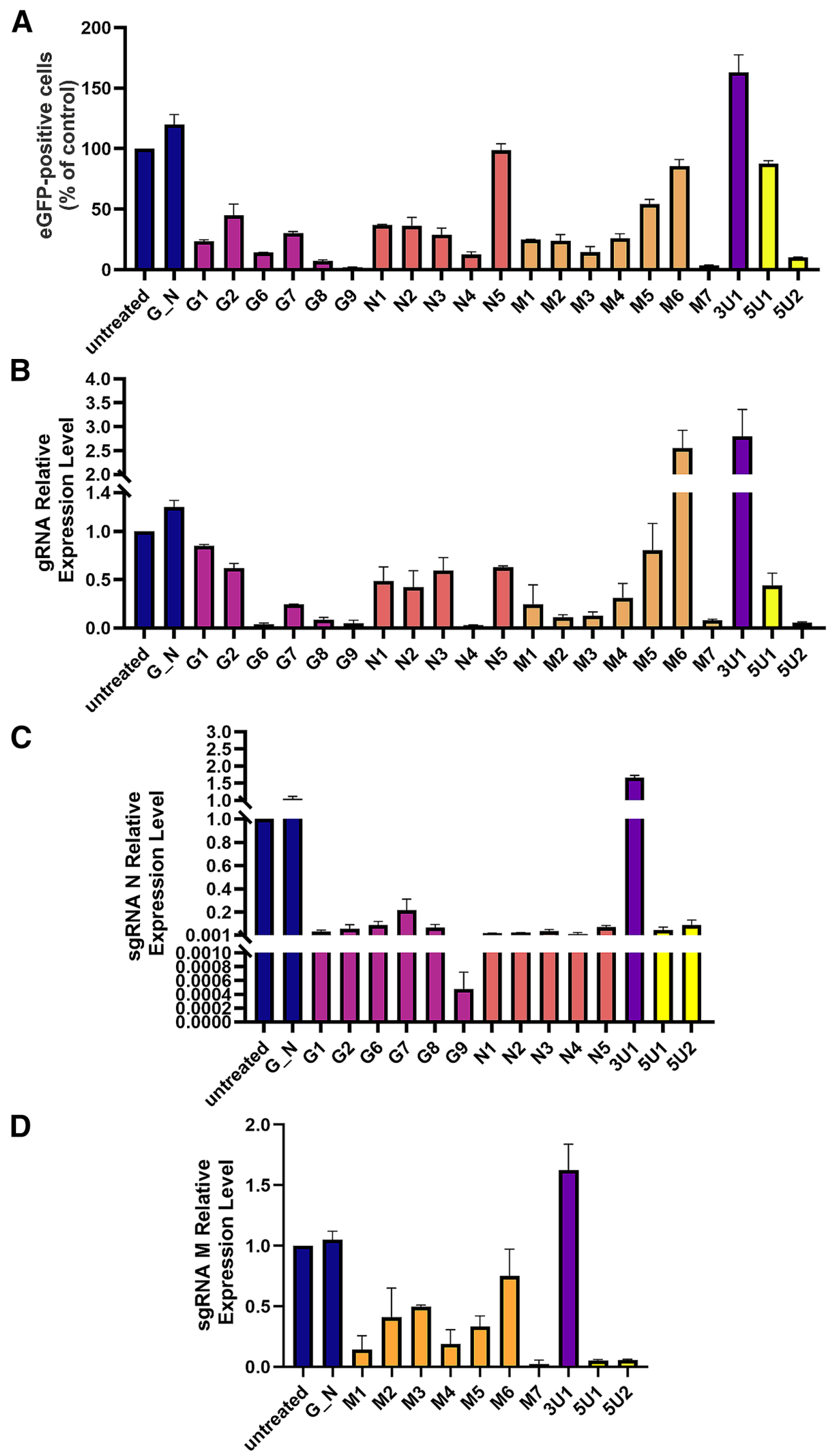

Figure 4. Effect of gapmers activity on pBAC-SARS-CoV-2_*S/eGFP_ΔE* in HEK293T cells

(A) Flow cytometry analysis of eGFP-positive HEK293T cells co-transfected with pBAC-SARS-CoV-2_*S/eGFP_ΔE* and gapmers (0.75 μM), showing the effect of gapmer-mediated inhibition on the replicon activity. (B–D) Relative levels of pBAC-SARS-CoV-2_*S/eGFP_ΔE* gRNA (B) and sgRNAs *N* and *M* (C and D, respectively) in HEK293T cells co-transfected with the replicon and gapmers (0.75 μM), quantified by RT-qPCR with *GAPDH* as the internal control. Bars in all panels represent the mean ± SD from two independent experiments.

RT-qPCR. Gapmers treatment reduced gRNA copy number by up to 96% and sgRNA *N* level by 80%–99%. Two gapmers, G6 and G8, consistently retained strong inhibitory activity (86%–96%) across all three assays (flow cytometry, RT-qPCR of gRNA, and sgRNA *N*; Figures 4A–4C; Table S2). Notably, G6 and G8 demonstrated inhibitory effects in both experimental models: pBAC-SARS-CoV-2_ *S*/*eGFP*_ *ΔE* system and in the eukaryotic expression system encoding sgRNA *N-eGFP* where G6 and G8 inhibitory activity was 74%.[46] These findings indicate that G6 and G8 have substantial antiviral potential. The observed discrepancies in gapmers activity, such as for G2 (95% inhibition of sgRNA *N* vs. 38% inhibition of gRNA, Table S2), may reflect differences in target site accessibility. For example, the G2 target region in gRNA may be buried within intra- and/or intermolecular RNA structures. Limited accessibility of this region could also impair recruitment of RNase H1; thus, even if the gapmer hybridizes to the target site, its effect may be mediated primarily through steric blocking rather than cleavage induction.

In parallel, we investigated a second group of newly designed gapmers that had not been tested in any prior system (Table S2; Figure 3). These 14 gapmers were designed to target RNA motifs within the SARS-CoV-2 genome, including regions located in the 3′ and 5′ UTRs (3U1, 5U1-5U3), as well as in the *N* (N1-N5) and *M* gene (M1-M7). Most of the oligonucleotides led to a substantial reduction in the number of eGFP-positive cells, with the most effective gapmers, such as N4, 5U2, and M7 reaching inhibitory activities of 87%–90% compared with the untreated control (Figure 4A). This effect was strongly corroborated by RT-qPCR analysis (Figures 4B–4D; Table S2). For N4, gRNA expression levels were reduced by 97%, while sgRNA *N* was decreased by 98%. 5U2 showed comparable activity, lowering gRNA levels by 95%, sgRNA *N* by 92% and sgRNA *M* by 94% (Figures 4B–4D). Gapmer M7 also showed consistent efficacy, with gRNA reduced by 92% and sgRNA *M* by 97% (Figures 4B and 4D). These values indicate that gapmers N4, 5U2, and M7 confirm their robust inhibitory activity at both the replication and transcriptional levels and have high antiviral potential. In contrast, gapmer 3U1, which targets the 29639–29657 region of the 3′UTR, did not reduce viral markers but instead increased the number of eGFP-positive cells in flow cytometry and was associated with higher levels of viral genomic and subgenomic RNAs, as demonstrated by RT-qPCR analysis (Figure 4). One of the gapmers (M2) was synthesized in a version containing pseudouridine (M3). Pseudouridine is a modification that enhances RNA stability, reduces off-target effects, and minimizes innate immune activation[50]; however, in this case, it did not affect antiviral potency, as M3 demonstrated comparable inhibitory activity to M2 in every experimental assay performed. Similarly, as for group of G gapmers (e.g., G2), discrepancies in N, U, and M gapmers activity against gRNA and sgRNA (N1, N2, N3, N5, 5U1, and M6; Table S2) may be attributed to variation in target region availability. N1 and N2, which reduced gRNA levels by 52% and 58%, respectively, were designed to target structural motifs present exclusively in sgRNA *N* and not in gRNA (Figure 3). Consistent with this, their inhibitory activity against sgRNA *N* reached 98%. Notably, the reduced activity against gRNA was in few cases associated with only a limited reduction in the number of the eGFP-positive cells (Table S2), suggesting that effective targeting of gRNA—and ideally both gRNA and sgRNA—yields the most pronounced antiviral effects.

Treatment with phosphorothioate, 2′-modified gapmer antisense oligonucleotides has been reported to modulate innate immune

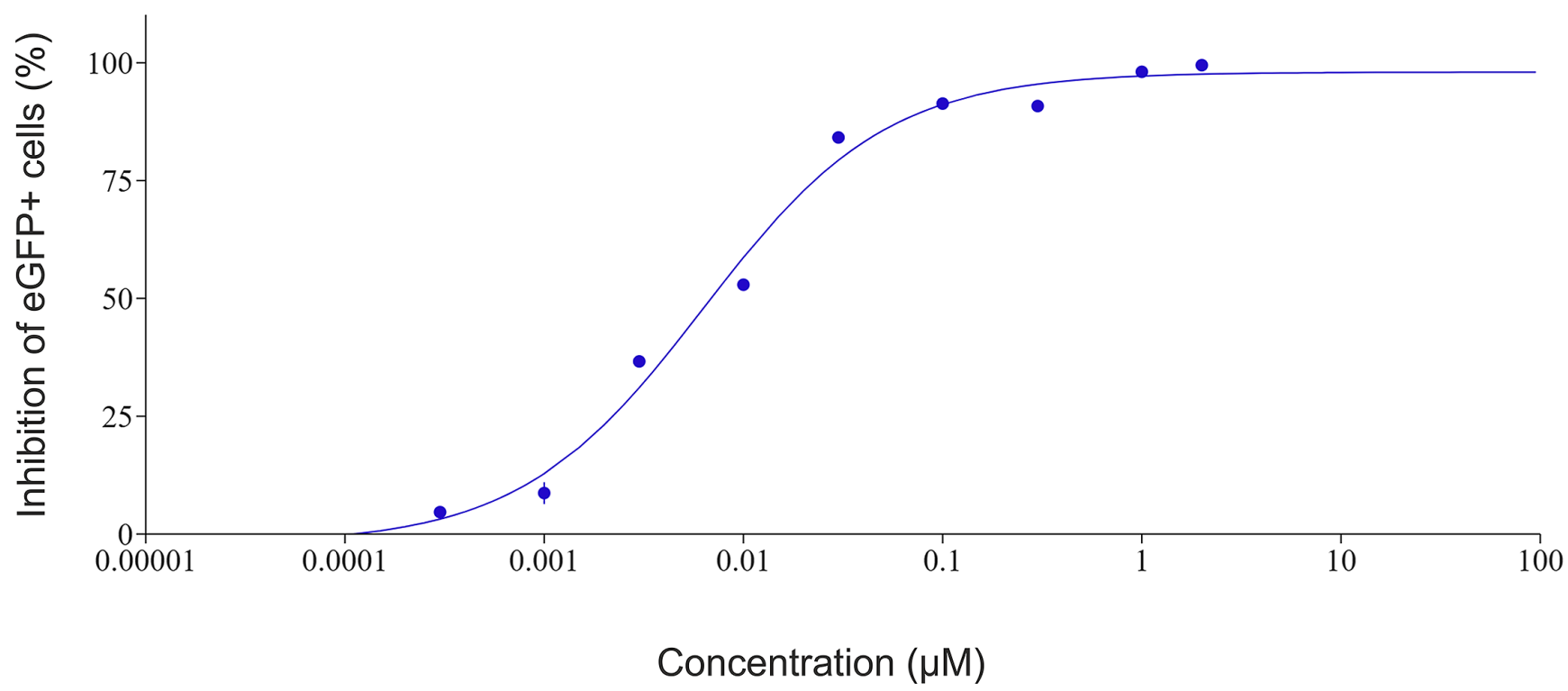

Figure 5. Dose-response analysis and EC_{50} determination for M7 against pBAC-SARS-CoV-2_*S/eGFP*_Δ*E* replicon system

Effect of M7 on replicon activity was evaluated at concentrations: 0.0001, 0.0003, 0.001, 0.003, 0.01, 0.03, 0.1, 0.3, 1, and 2 μM. Replicon activity was quantified by flow cytometry as the percentage of eGFP-positive cells. HEK293T cells transfected with the replicon alone were used as the 100% reference. The EC_{50} value was determined using the AAT Bioquest online calculator. Data points represent the mean ± SD from two independent experiments.

responses in a sequence- and chemistry-dependent manner.[51–54] To determine whether the antiviral effects observed here resulted from direct viral RNA targeting rather than nonspecific immune stimulation, we examined activation of the interferon pathway. Specifically, we measured the expression of the interferon-stimulated gene *MX1* in HEK293T cells transfected with each gapmer either alone or together with the replicon (Figure S5). Two gapmers, 3U1 and M6, were excluded from this analysis because they showed only weak inhibitory activity or a stimulatory effect on replicon activity. In most cases, gapmer delivery alone did not significantly increase *MX1* expression. Only one gapmer, G9, appeared to induce an innate immune response, although to a lower extent than polyI:C or the replicon. Moreover, co-transfection of HEK293T cells with G9 and the replicon did not result in a pronounced increase in *MX1* transcript levels, suggesting that the reduction in replicon-derived innate immune stimulation outweighed the intrinsic immunostimulatory activity of this gapmer. Importantly, in most cases, co-delivery of gapmers and the replicon substantially reduced activation of the interferon-responsive pathway compared with cells transfected with the replicon alone, in which interferon signaling was strongly induced. This reduction likely reflects inhibition of replicon activity, which in turn decreases induction of the interferon-stimulated gene *MX1*. Exceptions to this trend were observed for three gapmers, G1, G2, and M5, for which *MX1* expression in cells co-transfected with the replicon and gapmer remained at an intermediate level. This likely reflects residual innate immune activation resulting from incomplete suppression of replicon RNA, consistent with the limited reduction in gRNA levels observed by RT-qPCR (16%–38%; Table S2). Together with the low *MX1* expression in cells transfected with gapmers alone, the reduced *MX1* levels relative to the replicon-only control indicate that *MX1* induction primarily reflects replicon burden rather than nonspecific immune activation by the gapmers. In this work, particular attention was given to five gapmers, 5U2, G6, G8, N4, and M7, which showed strong antiviral activity and therefore represent promising lead candidates. For these gapmers, co-transfection with the replicon was associated with reduced *MX1* expression compared with replicon-only transfection, consistent with decreased replicon burden and consequently reduced innate immune activation. These results indicate that their antiviral effects are unlikely to be mediated by induction of the innate immune response and are instead consistent with a direct mechanism involving viral RNA targeting.

M7 emerges as the lead antiviral gapmer targeting sgRNA M

Based on the experiments described earlier, we identified a subset of gapmers with substantial activity against SARS-CoV-2: one targeting the 5′ UTR region (5U2), three targeting the *N* gene (G6, G8, and N4), and one targeting the *M* gene (M7; Table S2). We first examined whether the gapmer target sequences were conserved across major SARS-CoV-2 variants circulating between 2019 and 2026 (Table S3). The analysis was performed as described previously,[55] with the Nextstrain database used as the source of representative viral sequences. The target sites of G6, G8, N4, and M7 were fully conserved across all analyzed variants. The 5U2 target site was also fully conserved in most variants, with a single-nucleotide mismatch identified in the Delta variant (Table S3). We next performed a pilot dose-response experiment and evaluated the activity of these gapmers at four concentrations: 0.25, 0.5, 0.75, and 1.5 μM (Figure S6). Antiviral activity was assessed by flow cytometric quantification of the percentage of eGFP-positive cells following co-transfection of the replicon with gapmers. Among the tested candidates, M7 clearly showed the strongest antiviral effect. Even at the lowest tested concentration, 0.25 μM, M7 reduced replicon activity by 95%. Using the same assay, we determined the EC_{50} of M7 to be 6.3 ± 0.14 nM (Figure 5). In addition, at a concentration of 0.75 μM, M7 reduced eGFP fluorescence intensity by 82% in eGFP-positive cells, as determined by flow cytometry (Table S2; Figure S7). Importantly, cell viability assays performed at 0.75 μM gapmer concentration showed no detectable cytotoxic effect of M7 (Figure S8). Taken together, these findings identify M7 as the lead candidate emerging from our antiviral screening.

DISCUSSION

In this work, we present the non-infectious pBAC-SARS-CoV-2_ *S*/*eGFP*_ Δ*E* replicon as a versatile tool for investigating the SARS-CoV-2 replication cycle and for evaluating antiviral strategies under BSL-2 conditions. The BAC-based reverse genetics system enables the stable maintenance of large DNA fragments in bacteria. Similar

BAC plasmid-based strategies have previously been employed to generate full-length cDNA clones and non-infectious replicons of numerous coronaviruses, including HCoV-OC43,[56] TGEV,[57] FCoV,[58] SARS-CoV,[5,25,43] MERS-CoV,[59,60] SADS-CoV,[61] and SARS-CoV-2.[7,10,62] The advantage of this system is the reduced toxicity and instability of nsp8-nsp12 replicase gene sequences,[2,24] achieved through the *parB* and *parC* genes derived from *Escherichia coli* F-factor,[63,64] which limit plasmid replication to one or two copies per cell. This low-copy maintenance minimizes DNA rearrangements compared to YAC system.[65] The BAC approach also facilitates targeted genome modification (e.g., by restriction enzyme cloning).[2,5,11] For efficient assembly, cDNA clones are typically divided into 4–5 kb fragments.[2] Unique restriction sites are essential for cloning and reconstitution, and, when absent, can be introduced by silent mutations, as implemented in this study. These engineered sites can also function as genetic markers for full-length cDNA clones.[2,24] Major advantage of the CMV-driven BAC strategy, compared with the T7-driven approaches applied in the *in vitro* ligation-based strategy[66] and in some BAC systems,[9] is that it supports direct rescue of virus or recovery of viral molecules (RNAs and proteins) from a cDNA clone without requiring *in vitro* transcription. In this design, the CMV promoter and BGHpA (or SV40 termination and polyadenylation sequences) downstream of the viral genome drive synthesis of capped viral RNAs in mammalian cells, while HDVr ensures precise formation of the authentic 3′ end of the gRNA.[11,67] In contrast, the T7-based methods approach requires *in vitro* RNA synthesis, capping, and subsequent RNA transfection. These additional steps complicate experiments with large genomes such as SARS-CoV-2, and often yield heterogeneous RNA products due to abortive transcription or misassembled templates. In conclusion, *in vivo* transcription of CMV-driven BAC plasmids in host cells offers apparent advantages over T7-based methods, being more stable, efficient, and experimentally convenient.

The pBAC-SARS-CoV-2_ *S/eGFP_ΔE* represents a distinct replicon architecture. Existing non-infectious SARS-CoV-2 BAC-based systems suitable for BSL-2 research have relied on deletion of the *S* gene,[24,68] multi-gene deletion strategies removing *S*, *E*, and *M*[8] or *S*, *ORF6*, and *ORF7a*,[69] or highly simplified minigenome systems containing only the UTRs, *ORF1ab*, and *N*.[5,6,23,62] A *trans*-complementation BAC-based system lacking the *E/M* region and generating single-round virions has been proposed as a vaccine platform.[70] A related system with an isolated *E* deletion has been used to probe the functional consequences of *E* loss, demonstrating that Δ*E* attenuates SARS-CoV-2 replication by an altered pattern of S protein localization, enhanced cell-to-cell transmission, and reorganization of the ER-Golgi intermediate compartment essential for virion assembly.[71] In our construct, the combined deletion of *S*—required for host-cell entry and *E*—important for virion assembly, budding, and maturation—abolishes infectious-particle formation. This yields a safe, assembly-deficient replicon compatible with BSL-2 handling. Notably, retention of the *M* gene enables studies of *M*-associated RNA structures central to the replication cycle, as well as M-dependent processes that cannot be interrogated in systems with broader gene deletions. The combination of *S* and *E* loss could also make this construct a useful component of a *trans*-complementation system for dissecting the functional consequences of these gene deletions on viral replication and assembly.

In this study, we used the pBAC-SARS-CoV-2_ *S/eGFP_ΔE* replicon for antiviral screening of antisense gapmers. This screening identified a focused group of potent inhibitors of SARS-CoV-2 replication—G6, G8, N4, M7, and 5U2—with M7 showing the strongest antiviral activity. Surprisingly, one of the gapmers, 3U1, resulted in an increased number of eGFP reporter-positive cells and elevated viral RNA levels across all assays (Figure 4; Table S2). The 3U1 targets the 3′PK hairpin (Figure 3), a structural element capable of adopting a stem-loop or pseudoknot conformation. This conformational switch has been linked to transcription initiation in coronaviruses.[30,72] The stimulatory effect of 3U1 was unexpected, as deletion and mutations disrupting the pseudoknot formation are typically lethal for viral replication.[73–75] One possible explanation is that 3U1 partially nicks or/and leads to RNA conformation that favors replication or transcription. In this context, targeting the PK hairpin may paradoxically relieve a structural bottleneck in replication control, thereby enhancing viral replication. In addition, binding of 3U1 to a structured double-stranded RNA region could alter local folding, inadvertently increasing transcript stability or translation efficiency. Formation of a gapmer-RNA hybrid in an overall rigid structural context may also impair RNase H1 recruitment, resulting in non-productive binding that stabilizes the RNA rather than inducing degradation.[76] An alternative explanation for the stimulatory activity of 3U1 involves indirect effects on host gene expression. Although 3U1 did not exhibit cytotoxicity in our assays (Figure S8), BLAST analysis identified potential off-targets in the human transcriptome with 68%–74% complementarity. These include serine protease 23 (PRSS23) mRNA (*PRSS23*), doublecortin domain-containing protein 1 mRNA (*DCDC1*), myocardial infarction-associated transcript (*MIAT*), and the uncharacterized transcripts *LOC105372310* and *LOC105377223* ncRNA. The interactions between 3U1 and these transcripts could influence virus-host dynamics. Notably, PRSS23 is moderately upregulated in endothelial cells upon spike protein stimulation and has been reported to interact with SARS-CoV-2 accessory proteins ORF8 and ORF9c,[77] both implicated in immune escape and antiviral responses. This suggests that PRSS23 may contribute to host response mechanisms during SARS-CoV-2 infection.[78] Thus, inadvertent targeting of *PRSS23* by 3U1 could alter virus-host interactions in a manner that ultimately promotes viral replication.

The G6, G8, N4, M7, and 5U2 gapmers exhibited antiviral activity by targeting structurally defined RNA motifs located within conserved regions of the SARS-CoV-2 genome[46,47] (Table S3), highlighting their potential as lead candidates for therapeutic development. Notably, gapmer 5U2 targets the 5′ UTR SL5a stem-loop within the highly stable and conserved SL5 domain, which adopts a T-shaped tertiary architecture.[79,80] Because the apical region of SL5a has been reported to interact with the

SARS-CoV-2 nucleocapsid protein,[81] targeting this element may perturb both local RNA structure contributing to the stability of the 5′-proximal region and functionally relevant RNA-protein interactions. Gapmers G6, G8, N4 recognize hairpin motifs and internal helix within the *N* gene, while M7 targets a stem-loop structure of the *M* gene (Figure 3). Although identical sequences may adopt somewhat different conformations depending on whether they occur in genomic or subgenomic RNA contexts,[29,46–48,82] the target motifs of all four gapmers are retained in both the full-length gRNA and the corresponding sgRNAs (Figure 3). The observed reductions in both genomic and subgenomic RNA levels are consistent with these target sites remaining sufficiently accessible to gapmer binding in both RNA contexts (Table S2). This may not be the case for all gapmers. For example, the target motifs of G1, G2, N3, N5, and 5U1, although present in the gRNA, may be structurally occluded or engaged in interactions that limit their accessibility. Consistent with this possibility, these gapmers exhibited weaker overall inhibition of replicon activity, as reflected by the percentage of eGFP-positive cells (Table S2). Simultaneous targeting of genomic and subgenomic RNAs may therefore contribute to the strong inhibitory effects of the G6, G8, N4, and M7 gapmers by promoting depletion of both RNA species, thereby limiting genome replication and reducing the expression of essential structural proteins. This interpretation is consistent with previous studies targeting SARS-CoV-2 RNA structures shared between the gRNA and all sgRNAs, including the 3′-terminal SPEAR motif[83] and the SL1 structure within the common 5′ leader sequence.[84] Antisense oligonucleotides directed against these elements reduced infectious viral titers, further highlighting the therapeutic potential of targeting structural features present across multiple viral RNA species.

Among the selected gapmers, M7 was clearly the most potent, with an EC_{50} of 6.3 ± 0.14 nM (Figure 5). Its strong antiviral activity was consistently observed across all assays, including RT-qPCR analysis of gRNA and sgRNA *M*, flow cytometric quantification of the percentage of eGFP-positive cells, and analysis of eGFP fluorescence intensity (Figure 4; Table S2). Moreover, M7 did not measurably activate the cellular innate immune response (Figure S5), indicating that its antiviral effect is likely mediated by direct targeting of viral RNA rather than nonspecific immune stimulation. Its favorable profile was further supported by the absence of detectable cytotoxicity (Figure S8) and by the complete conservation of its target site across major SARS-CoV-2 variants (Table S3). Together, these findings position M7 as the most promising candidate identified in our antiviral screening. This result is particularly noteworthy because the *M* gene has received relatively little attention as a therapeutic RNA target in SARS-CoV-2. Most structure-guided RNA-targeting strategies have focused on highly conserved *cis*-regulatory elements, particularly the 5′ leader and untranslated regions,[84–86] as well as the programmed-1 ribosomal frameshifting element,[87–89] all of which are directly involved in essential steps of viral replication and gene expression. Although siRNAs targeting structural genes, including *M*, have been proposed,[90,91] experimentally validated therapies directed specifically against the *M* coding region remain scarce.

In our previous work,[47] we reported the first experimentally informed secondary-structure model of SARS-CoV-2 sgRNA *M*, encompassing both the 5′ leader region and the *M* coding sequence, and identified structural features with potential therapeutic relevance. We subsequently applied a similar structure-guided approach to sgRNA *N*, experimentally determined its secondary structure, and used this information to design gapmers, siRNAs, and ASOs targeting structurally accessible regions within the *N* coding sequence.[46] Two of the gapmers from that study, G6 and G8, were further validated here and confirmed to possess strong antiviral activity.

Together, these findings indicate that SARS-CoV-2 protein-coding RNAs contain well-defined and therapeutically exploitable structural elements. In this context, the exceptional potency of M7 identifies the comparatively underexplored *M* coding region as a promising target for antisense-mediated inhibition of SARS-CoV-2. Future studies should validate M7 and the other lead gapmers in respiratory epithelial models and, subsequently, against infectious SARS-CoV-2, including clinically relevant variants, to determine whether their potent activity in the replicon system translates into effective inhibition of authentic viral infection.

MATERIALS AND METHODS

Construction of pBAC-SARS-CoV-2_*S/eGFP*_Δ*E* replicon

The pBAC-SARS-CoV-2 replicon was constructed from eight DNA fragments (F1-F8, Figure 1) based on the SARS-CoV-2 strain Slovakia/SK-BMC5/2020 (EVAg – European Virus Archive – Global) and pBeloBAC11 vector.[42] Assembly of the pBAC-SARS-CoV-2 replicon was performed stepwise using Polymerase Incomplete Primer Extension (PIPE),[92] Gibson Assembly (GeneArt Gibson Assembly HiFi, Thermo Fisher Scientific), and restriction enzyme cloning (FastDigest Restriction Enzymes, Thermo Fisher Scientific; T4 DNA Ligase - EURx).

F1 was assembled in the pUC19 vector. Multiple cloning site (MCS) containing *Kas*I, *Sgr*DI, *Bsi*WI, *Mre*I, *Pac*I, *Mlu*I, *Kfl*I, *Asi*SI, *Asc*I, and *Hind*III was introduced into the pUC19 vector using F1.1.F and F1.1.R primers (PCR product: pUC19-MST-1, Table S4). The pUC19-MCS-1 sequence was *Mlu*I-digested and ligated with T4 DNA ligase to obtain pUC19-(F1:MCS). The cytomegalovirus promoter/enhancer (CMVp) was cloned into pUC19-(F1:MCS) by PIPE using F.1.2.VF, F.1.2.VR and F.1.2.IF, F.1.2.IR primers (PCR products: pUC19-MST-2 and CMV, respectively, Table S4). The sequence encoding SARS-CoV-2 5′ UTR and first 86 bp of *ORF1* was amplified through F.1.3.IF, F.1.3.IR primers (PCR product: 5′ UTR-*ORF1*86, Table S4) and PIPE cloned into pUC19-(F1:CMV-MCS) amplified by F.1.3.VF, F.1.3.VR (PCR product: pUC19-CMV-MST, Table S4). Complete F1 (CMV-5′ UTR-*ORF1*86-MCS) was cloned into pBeloBAC11 using *Kas*I and *Hind*III (Figure 1D).

The F2-F6 DNA elements (covering sequence of SARS-CoV-2 replicase complex, *ORF1ab*) were PCR-amplified from cDNA SARS-CoV-2 Slovakia/SK-BMC5/2020 using the following sets of primers: F.2F and F.2R, F.3F and F.3R, F.4F and F.4R, F.5F and F.5R, F.6F, and

F.6R (Table S4; Figure S1). The DNA fragments (F2, F3, F4, F5, and F6) were then stepwise inserted into pBeloBAC11-F1 by restriction enzyme cloning (*Bsi*WI and *Mre*I; *Mre*I and *Pac*I; *Pac*I and *Mlu*; *Mlu*I and *Kfl*; *Kfl*I and *Asi*SI, respectively, Figure 1D).

F7, containing the following sequences: (1) the region of SARS-CoV-2 genome spanning between *ORF S* and 3′ UTR, (2) hepatitis delta virus ribozyme (HDVr), and (3) bovine growth hormone termination and polyadenylation signal (BGHpA); was assembled from the F7.1 and F7.2 synthetic genes ordered from Twist Bioscience (Figures 1C; Table S1). The F7.1 and F7.2 gene fragments were provided in the pTwist-Amp-MC and pET-21(+) vectors, respectively. The F.1 sequence contains *S/eGFP* and *ΔE* modifications[25,43] (Figures 1B and 1C). The tract of 33 adenosines (pA) was introduced downstream of the SARS-CoV-2 3′ UTR (F7.2) by PIPE cloning with use of F.7.2.1F and F.7.2.1R primers (PCR product: F7.2poly(A)-pET-21(+)-1; Table S4). Then, F7 was constructed by Gibson Assembly of *ORF1*-1148-3′, F.7.1, and F7.2 poly(A)-pET-21(+) fragments amplified by F.7.I1F, F.7.I1R, F.7.I2F, F.7.I2R, F.7.VF, and F.7.VR primers (Table S4). Finally, we introduced F7 (amplified by F.7.I2F and F7.R primers, Table S4; Figure S1) into pBeloBAC11-F1-F2-F3-F4-F5-F6 (F8) using *Asi*SI and *Asc*I restriction enzymes (Figure 1D). The sequences of the intermediate constructs and the final pBAC-SARS-CoV-2_*S/eGFP*_Δ*E* were confirmed by sequencing.

Bacterial transformation

Plasmids pUC19, twist-Amp-MC, and pET-21(+) were introduced into DH10B or DH5α competent cells (Thermo Fisher Scientific) by heat shock. Intermediate vectors derived from pBeloBAC11 and the replicon pBAC-SARS-CoV-2_*S/eGFP*_Δ*E* were electroporated into DH10B electrocompetent cells (Thermo Fisher Scientific) using 0.2 cm cuvettes and MicroPulser Electroporator (BioRad) – program Ec2 (voltage ∼2.5 kV, pulse length 4–5 ms).

Plasmid and BAC purification

Plasmids used in cloning reactions, pUC19, pTwist-Amp-MC, and pET-21(+), were purified on a small scale using the Plasmid Miniprep DNA Purification Kit (EURx). Intermediate vectors derived from pBeloBAC11 and the pBAC-SARS-CoV-2_*S/eGFP*_Δ*E* replicon were purified by Large-Construct Kit (QIAGEN) to obtain transfection-grade large-construct DNA cleared from genomic DNA contamination.

HEK293T cell culture

HEK293T (Human embryonic kidney, American Type Culture Collection, Manassas, VA, USA) cells were grown in Dulbecco's Modified Eagle's Medium (DMEM, Gibco) containing 10% fetal bovine serum (FBS, Gibco) and 1% penicillin/streptomycin solution (100 U/mL of penicillin and 100 μg/mL of streptomycin). The cells were grown at 37°C in a humidified incubator with a 5% CO_2 atmosphere.

Transfection

24 h before transfection, HEK293T cells were seeded into a 24-well plate at 200,000 cells per well to achieve 80% confluence. Transfection was performed using Lipofectamine LTX (Invitrogen) by diluting 3 μg of DNA in Opti-MEM and 5 μL PLUS Reagent, and mixing with 5 μL of Lipofectamine diluted in Opti-MEM to a final volume of 100 μL per well. The gapmers were mixed with Lipofectamine LTX/DNA mixture, and complexes were incubated for 5 min at room temperature and added to cells containing 400 μL of DMEM (Dulbecco's Modified Eagle's Medium without FBS, penicillin/streptomycin solution, and L-glutamine); to a final volume of 500 μL per well. Transfection solution and cells were incubated for 15 h, then the transfection solution was replaced with fresh media and incubated at 37°C in a 5% CO_2 humidified incubator for the following 48 h. The transfection effect was evaluated by eGFP fluorescence, which was observed and imaged by fluorescence microscopy and flow cytometry. Furthermore, cells were harvested for RT-qPCR and western blot analysis.

Plaque assay

Vero E6 cells (3×10^5 cells/mL) were seeded in 6-well plates and incubated for 24 h at 37°C in a humidified incubator with 5% CO_2 to reach 90% confluence. Serial 10-fold dilutions of the supernatant collected from HEK193T cells transfected with the replicon were prepared (10^0, 10^{-1}, 10^{-2},10^{-3}, and 10^{-4}). Subsequently, 200 μL of each dilution was used to infect target cells. The cells were incubated for 1 h at 37°C in a 5% CO_2 incubator, with rocking every 15 min. Following incubation, the inoculum was carefully aspirated, and the cells were overlaid with Avicel-Medium containing 1.25% Avicel (FMC Biopolymer Germany GmbH, Hamburg, Germany), 10% Minimum Essential Medium (MEM) (Thermo Fisher Scientific), 0.01% DEAE-Dextran (Sigma Aldrich), 2.8% $NaHCO_3$ (Merck, Darmstadt, Germany), 1% Penicillin-Streptomycin (Sigma Aldrich), 0.2% BSA (Sigma Aldrich), and 1% L-Glutamine (Sigma Aldrich). Cells were incubated for 72 h at 37°C with 5% CO_2. After incubation, the overlay medium was removed, and the cells were washed twice with phosphate-buffered saline (PBS) (Thermo Fisher Scientific). Subsequently, the cells were fixed with 4% paraformaldehyde prepared in PBS for 30 min at room temperature. Fixed cells were then stained using a crystal violet solution consisting of 1% crystal violet (Thermo Fisher Scientific) and 20% methanol in water.

Reverse transcription and quantitative real-time PCR

Total RNA was extracted from cells co-transfected with the replicon and gapmers or from replicon-transfected cells treated with remdesivir, and purified using a NucleoSpin RNA Plus (Macherey-Nagel, Analytik, Jena, Germany) according to the manufacturer's protocol. The integrity of the isolated RNA was assessed using the TapeStation 4150 (Agilent) system. Reverse transcription was carried out using the SuperScript III (Invitrogen) and gene-specific primers (Table S5) for cDNA synthesis at a concentration of 0.25 μM. qPCR was performed to detect sgRNA *N*, sgRNA *M*, gRNA, and *GAPDH* mRNA by using Luna Universal qPCR Master Mix (New England Biolabs) according to the manufacturer's protocol. The expression level of sgRNA and gRNA was calculated by the relative threshold cycle (ΔΔCT) method using *GAPDH* mRNA as an internal control. Experiments were conducted in two biological and three technical replicates.

Flow cytometry

In each experiment, HEK293T cells co-transfected with the replicon and gapmers were harvested, resuspended in PBS, and then stained for 3 min at room temperature with propidium iodide (PI) viability dye at a concentration of 0.25 μg/mL. Two biological and two technical replicates for each condition were conducted, using the Guava easyCyte 12HT flow cytometer (Cytek Biosciences). In total, 20,000 events were recorded for each replicate. The PI dye (Ex/Em 535/617 nm) was excited using a 488 nm laser, and its signal was captured in the Red-B channel. Similarly, eGFP (Ex/Em of 398 and 475/509 nm) was excited by a 488 nm laser, and its signal was detected in the Green-B channel. The cells were first sorted into single-cell groups and then categorized based on their viability into live (PI−) and dead (PI+) groups. Both subsets were further sorted to identify those with a positive eGFP signal (eGFP+). The percentage of eGFP-positive cells and the mean fluorescence intensity (MFI) were determined. Data analysis was performed using FlowJo software, version 10 (Becton Dickinson).

Western blot assay

HEK293T cells co-transfected with the replicon and gapmers or replicon-transfected cells treated with remdesivir were harvested 48 h post-transfection and lysed by Laemmli Sample Buffer (Bio-Rad). The samples were then incubated for 15 min at 95°C. Denatured proteins were run on SDS-PAGE gels (4%–15% Mini-PROTEAN TGX Precast Protein Gels, Bio-Rad). Two gels containing replicates of the same samples were run simultaneously: one for viral protein detection and the other for actin detection (control, Figure S2). Proteins were transferred from the gels to nitrocellulose membranes (Bio-Rad) using a Trans-Blot Turbo Transfer System (Bio-Rad). Membranes were blocked with 5% BSA in TBS-T for 1 h at room temperature and then incubated overnight at 4°C with rabbit anti-nucleoprotein antibody (Abcam) or anti-actin antibody (Invitrogen). Subsequently, the membranes were incubated for 1 h at room temperature with anti-rabbit antibodies conjugated to horseradish peroxidase (HRP) (Sigma-Aldrich). Next, viral proteins and actin were detected using WESTAR NOVA 2.0 (Cyanagen) according to the manufacturer's protocol. Chemiluminescent signals were visualized by AZURE 400 Visible Fluorescent Imager.

Interferon response analysis

The interferon response was assessed by quantifying the expression of interferon-stimulated gene using RT-qPCR. For this analysis, total RNA was collected from HEK293T cells co-transfected with the replicon and gapmers or from cells transfected with gapmers alone. As a positive control polyinosinic:polycytidylic acid (poly(I:C)) (InvivoGen) was used. Cell transfection and RNA isolation were performed as described in the previous sections in materials and methods. In this experiment, cDNA synthesis was carried out using random hexamer primers according to the manufacturer's instructions (Thermo Fisher Scientific). Gene expression levels of interferon-stimulated *MX1* gene were quantified using gene-specific primers (Table S5). The housekeeping *GAPDH* gene was used as an internal normalization control. Relative gene expression levels were calculated using the $2^{-\Delta\Delta Ct}$ method.

Oligonucleotides

DNA primers used in PCR were purchased from Sigma-Aldrich. Long DNA fragments (>100 nt) and gapmers were synthesized using the phosphoramidite approach on a MerMade synthesizer, and subsequently deprotected and purified according to published protocols.[93–95] Gapmers were modified with 2'*O*-methylated RNA (2′OMe), locked nucleic acid (LNA), and phosphorothioate (PTO) backbone to enhance their thermodynamic and cellular stability. Additionally, gapmer M3 included pseudouridine residues (Table S2). Natural and modified phosphoramidites used for oligonucleotide synthesis were purchased from ChemGenes and GenePharma. The concentration and purity of all oligonucleotides were evaluated using a spectrophotometer UV (NanoDrop2000 Thermo Fisher Scientific, Waltham, MA, USA).

DATA AND CODE AVAILABILITY

All data associated with this study are available upon request.

ACKNOWLEDGMENTS

This work was supported by the National Science Centre grants 2020/39/B/NZ1/03054 and 2021/41/B/NZ1/03819 (to E.K.) and 2022/45/B/ST4/03586 (to R.K.). For Open Access publishing, the authors have applied a CC-BY public copyright license to any author-accepted manuscript (AAM) version arising from this submission. The authors would like to acknowledge the Laboratory of Single Cell Analyses, Institute of Bioorganic Chemistry, Polish Academy of Sciences for use of the Guava easyCyte 12HT, Cytek Biosciences, and Magdalena Trybus, MSc, for data analysis.

AUTHOR CONTRIBUTIONS

E.K. and A.R. conceived the idea. A.B.-G., A.R., J.P., and M.C. carried out the experiments. R.K. and E.K. performed chemical synthesis of oligonucleotides. A.R, A.B.-G., and E.K. analyzed and interpreted results. A.R., A.B.-G., and J.P. prepared the original draft of the manuscript with contributions from all the authors and revised the manuscript. E.K. supervised the project. All authors read and approved the final manuscript.

DECLARATION OF INTERESTS

The authors declare no competing interests.

DECLARATION OF GENERATIVE AI AND AI-ASSISTED TECHNOLOGIES IN THE WRITING PROCESS

During the preparation of this work, the authors used ChatGPT to improve clarity and language in the manuscript. The authors reviewed and edited the output as needed and take full responsibility for the content of the published article.

SUPPLEMENTAL INFORMATION

Supplemental information can be found online at https://doi.org/10.1016/j.omtn.2026.103098.

REFERENCES

1. V'Kovski, P., Kratzel, A., Steiner, S., Stalder, H., and Thiel, V. (2021). Coronavirus biology and replication: implications for SARS-CoV-2. Nat. Rev. Microbiol. *19*, 155–170. https://doi.org/10.1038/s41579-020-00468-6.
2. Wang, W., Peng, X., Jin, Y., Pan, J.A., and Guo, D. (2022). Reverse genetics systems for SARS-CoV-2. J. Med. Virol. *94*, 3017–3031. https://doi.org/10.1002/jmv.27738.
3. Jin, Q., Liu, R., Wang, W., Xie, J., Yan, F., Wang, T., Xiang, H., Xia, X., Li, J., Wang, X., and Gao, Y. (2025). Increased pathogenicity and transmission of SARS-CoV-2

Omicron XBB.1.9 subvariants, including HK.3 and EG.5.1, relative to BA.2. J. Virol. *99*, e0134225. https://doi.org/10.1128/jvi.01342-25.

4. Centers for Disease Control and Prevention (2025). Laboratory Biosafety Guidelines for working with SARS-CoV-2. https://www.cdc.gov/covid/php/lab/index.html.

5. Almazán, F., Dediego, M.L., Galán, C., Escors, D., Alvarez, E., Ortego, J., Sola, I., Zuñiga, S., Alonso, S., Moreno, J.L., et al. (2006). Construction of a severe acute respiratory syndrome coronavirus infectious cDNA clone and a replicon to study coronavirus RNA synthesis. J. Virol. *80*, 10900–10906. https://doi.org/10.1128/JVI.00385-06.

6. Nguyen, H.T., Falzarano, D., Gerdts, V., and Liu, Q. (2021). Construction of a Noninfectious SARS-CoV-2 Replicon for Antiviral-Drug Testing and Gene Function Studies. J. Virol. *95*, e0068721. https://doi.org/10.1128/JVI.00687-21.

7. Ye, C., Chiem, K., Park, J.G., Oladunni, F., Platt, R.N., Anderson, T., Almazan, F., de la Torre, J.C., and Martinez-Sobrido, L. (2020). Rescue of SARS-CoV-2 from a Single Bacterial Artificial Chromosome. mBio *11*, e02168-20. https://doi.org/10.1128/mBio.02168-20.

8. He, X., Quan, S., Xu, M., Rodriguez, S., Goh, S.L., Wei, J., Fridman, A., Koeplinger, K.A., Carroll, S.S., Grobler, J.A., et al. (2021). Generation of SARS-CoV-2 reporter replicon for high-throughput antiviral screening and testing. Proc. Natl. Acad. Sci. USA *118*, e2025866118. https://doi.org/10.1073/pnas.2025866118.

9. Zhang, Y., Song, W., Chen, S., Yuan, Z., and Yi, Z. (2021). A bacterial artificial chromosome (BAC)-vectored noninfectious replicon of SARS-CoV-2. Antivir. Res. *185*, 104974. https://doi.org/10.1073/pnas.2025866118.

10. Rihn, S.J., Merits, A., Bakshi, S., Turnbull, M.L., Wickenhagen, A., Alexander, A.J.T., Baillie, C., Brennan, B., Brown, F., Brunker, K., et al. (2021). A plasmid DNA-launched SARS-CoV-2 reverse genetics system and coronavirus toolkit for COVID-19 research. PLoS Biol. *19*, e3001091. https://doi.org/10.1371/journal.pbio.3001091.

11. Almazán, F., Sola, I., Zuñiga, S., Marquez-Jurado, S., Morales, L., Becares, M., and Enjuanes, L. (2014). Coronavirus reverse genetic systems: infectious clones and replicons. Virus Res. *189*, 262–270. https://doi.org/10.1016/j.virusres.2014.05.026.

12. van den Worm, S.H.E., Eriksson, K.K., Zevenhoven, J.C., Weber, F., Züst, R., Kuri, T., Dijkman, R., Chang, G., Siddell, S.G., Snijder, E.J., et al. (2012). Reverse genetics of SARS-related coronavirus using vaccinia virus-based recombination. PLoS One *7*, e32857. https://doi.org/10.1371/journal.pone.0032857.

13. Yount, B., Curtis, K.M., Fritz, E.A., Hensley, L.E., Jahrling, P.B., Prentice, E., Denison, M.R., Geisbert, T.W., and Baric, R.S. (2003). Reverse genetics with a full-length infectious cDNA of severe acute respiratory syndrome coronavirus. Proc. Natl. Acad. Sci. USA *100*, 12995–13000. https://doi.org/10.1073/pnas.1735582100.

14. Xie, X., Muruato, A., Lokugamage, K.G., Narayanan, K., Zhang, X., Zou, J., Liu, J., Schindewolf, C., Bopp, N.E., Aguilar, P.V., et al. (2020). An Infectious cDNA Clone of SARS-CoV-2. Cell Host Microbe *27*, 841–848.e3. https://doi.org/10.1016/j.chom.2020.04.004.

15. Zhang, Q.Y., Deng, C.L., Liu, J., Li, J.Q., Zhang, H.Q., Li, N., Zhang, Y.N., Li, X.D., Zhang, B., Xu, Y., and Ye, H.Q. (2021). SARS-CoV-2 replicon for high-throughput antiviral screening. J. Gen. Virol. *102*, 001583. https://doi.org/10.1099/jgv.0.001583.

16. Ju, X., Zhu, Y., Wang, Y., Li, J., Zhang, J., Gong, M., Ren, W., Li, S., Zhong, J., Zhang, L., et al. (2021). A novel cell culture system modeling the SARS-CoV-2 life cycle. PLoS Pathog. *17*, e1009439. https://doi.org/10.1371/journal.ppat.1009439.

17. Thi Nhu Thao, T., Labroussaa, F., Ebert, N., V'Kovski, P., Stalder, H., Portmann, J., Kelly, J., Steiner, S., Holwerda, M., Kratzel, A., et al. (2020). Rapid reconstruction of SARS-CoV-2 using a synthetic genomics platform. Nature *582*, 561–565. https://doi.org/10.1038/s41586-020-2294-9.

18. Ricardo-Lax, I., Luna, J.M., Thao, T.T.N., Le Pen, J., Yu, Y., Hoffmann, H.H., Schneider, W.M., Razooky, B.S., Fernandez-Martinez, J., Schmidt, F., et al. (2021). Replication and single-cycle delivery of SARS-CoV-2 replicons. Science *374*, 1099–1106. https://doi.org/10.1126/science.abj8430.

19. Wang, B., Zhang, C., Lei, X., Ren, L., Zhao, Z., Wang, J., and Huang, H. (2021). Construction of Non-infectious SARS-CoV-2 Replicons and Their Application in Drug Evaluation. Virol. Sin. *36*, 890–900. https://doi.org/10.1007/s12250-021-00369-9.

20. Amarilla, A.A., Sng, J.D.J., Parry, R., Deerain, J.M., Potter, J.R., Setoh, Y.X., Rawle, D.J., Le, T.T., Modhiran, N., Wang, X., et al. (2021). A versatile reverse genetics platform for SARS-CoV-2 and other positive-strand RNA viruses. Nat. Commun. *12*, 3431. https://doi.org/10.1038/s41467-021-23779-5.

21. Torii, S., Ono, C., Suzuki, R., Morioka, Y., Anzai, I., Fauzyah, Y., Maeda, Y., Kamitani, W., Fukuhara, T., and Matsuura, Y. (2021). Establishment of a reverse genetics system for SARS-CoV-2 using circular polymerase extension reaction. Cell Rep. *35*, 109014. https://doi.org/10.1016/j.celrep.2021.109014.

22. Luo, Y., Yu, F., Zhou, M., Liu, Y., Xia, B., Zhang, X., Liu, J., Zhang, J., Du, Y., Li, R., et al. (2021). Engineering a Reliable and Convenient SARS-CoV-2 Replicon System for Analysis of Viral RNA Synthesis and Screening of Antiviral Inhibitors. mBio *12*, e02754-20. https://doi.org/10.1128/mBio.02754-20.

23. Tanaka, T., Saito, A., Suzuki, T., Miyamoto, Y., Takayama, K., Okamoto, T., and Moriishi, K. (2022). Establishment of a stable SARS-CoV-2 replicon system for application in high-throughput screening. Antiviral Res. *199*, 105268. https://doi.org/10.1016/j.antiviral.2022.105268.

24. Jin, Y.Y., Lin, H., Cao, L., Wu, W.C., Ji, Y., Du, L., Jiang, Y., Xie, Y., Tong, K., Xing, F., et al. (2021). A Convenient and Biosafe Replicon with Accessory Genes of SARS-CoV-2 and Its Potential Application in Antiviral Drug Discovery. Virol. Sin. *36*, 913–923. https://doi.org/10.1007/s12250-021-00385-9.

25. Wang, J.M., Wang, L.F., and Shi, Z.L. (2008). Construction of a non-infectious SARS coronavirus replicon for application in drug screening and analysis of viral protein function. Biochem. Biophys. Res. Commun. *374*, 138–142. https://doi.org/10.1016/j.bbrc.2008.06.129.

26. Madhugiri, R., Nguyen, H.V., Slanina, H., and Ziebuhr, J. (2024). Alpha- and beta-coronavirus cis-acting RNA elements. Curr. Opin. Microbiol. *79*, 102483. https://doi.org/10.1016/j.mib.2024.102483.

27. Pekarek, L., Zimmer, M.M., Gribling-Burrer, A.S., Buck, S., Smyth, R., and Caliskan, N. (2023). Cis-mediated interactions of the SARS-CoV-2 frameshift RNA alter its conformations and affect function. Nucleic Acids Res. *51*, 728–743. https://doi.org/10.1093/nar/gkac1184.

28. Yao, Y.C., Yang, C.C., Wang, M., Hsieh, F.C., Lin, C.H., Hsu, H.W., Lai, C.C., Wang, W.C., Kuo, C.Y., Yang, C.Y., and Wu, H.Y. (2025). Linking the function of cis-acting RNA elements to coronavirus replication using interactomes. J. Gen. Virol. *106*. https://doi.org/10.1099/jgv.0.002070.

29. Lan, T.C.T., Allan, M.F., Malsick, L.E., Woo, J.Z., Zhu, C., Zhang, F., Khandwala, S., Nyeo, S.S.Y., Sun, Y., Guo, J.U., et al. (2022). Secondary structural ensembles of the SARS-CoV-2 RNA genome in infected cells. Nat. Commun. *13*, 1128. https://doi.org/10.1038/s41467-022-28603-2.

30. Ohyama, T., Osawa, T., Sekine, S.I., and Ishii, Y. (2024). NMR Studies of Genomic RNA in 3' Untranslated Region Unveil Pseudoknot Structure that Initiates Viral RNA Replication in SARS-CoV-2. JACS Au *4*, 1323–1333. https://doi.org/10.1021/jacsau.3c00641.

31. Szczesniak, I., Baliga-Gil, A., Jarmolowicz, A., Soszynska-Jozwiak, M., and Kierzek, E. (2023). Structural and Functional RNA Motifs of SARS-CoV-2 and Influenza A Virus as a Target of Viral Inhibitors. Int. J. Mol. Sci. *24*, 1232. https://doi.org/10.3390/ijms24021232.

32. Haddox, H.K., Angehrn, G., Sesta, L., Jennings-Shaffer, C., Temple, S.D., Galloway, J.G., Hinrichs, A.S., DeWitt, W.S., Bloom, J.D., Iv, F.A.M., and Neher, R. (2025). The mutation rate of SARS-CoV-2 is highly variable between sites and is influenced by sequence context, genomic region, and RNA structure. Nucleic Acids Res. *53*, gkaf503. https://doi.org/10.1093/nar/gkaf503.

33. Hensel, Z. (2025). Secondary structure of the SARS-CoV-2 genome is predictive of nucleotide substitution frequency. eLife *13*, RP98102. https://doi.org/10.7554/eLife.98102.

34. Bassett, M., Salemi, M., and Rife Magalis, B. (2022). Lessons Learned and Yet-to-Be Learned on the Importance of RNA Structure in SARS-CoV-2 Replication. Microbiol. Mol. Biol. Rev. *86*, e0005721. https://doi.org/10.1128/mmbr.00057-21.

35. Bai, Z., Cao, Y., Liu, W., and Li, J. (2021). The SARS-CoV-2 Nucleocapsid Protein and Its Role in Viral Structure, Biological Functions, and a Potential Target for Drug or Vaccine Mitigation. Viruses *13*, 1115. https://doi.org/10.3390/v13061115.

36. Tang, Y., Tang, K., Hu, Y., Ye, Z.W., Luo, W., Luo, C., Cao, H., Wang, R., Yue, X., Liu, D., et al. (2024). M protein ectodomain-specific immunity restrains SARS-CoV-2 variants replication. Front. Immunol. *15*, 1450114. https://doi.org/10.3389/fimmu.2024.1450114.

37. Grossoehme, N.E., Li, L., Keane, S.C., Liu, P., Dann, C.E., 3rd, Leibowitz, J.L., and Giedroc, D.P. (2009). Coronavirus N protein N-terminal domain (NTD) specifically binds the transcriptional regulatory sequence (TRS) and melts TRS-cTRS RNA duplexes. J. Mol. Biol. *394*, 544–557. https://doi.org/10.1016/j.jmb.2009.09.040.

38. Zúñiga, S., Cruz, J.L.G., Sola, I., Mateos-Gómez, P.A., Palacio, L., and Enjuanes, L. (2010). Coronavirus nucleocapsid protein facilitates template switching and is required for efficient transcription. J. Virol. *84*, 2169–2175. https://doi.org/10.1128/JVI.02011-09.

39. Zúñiga, S., Sola, I., Moreno, J.L., Sabella, P., Plana-Durán, J., and Enjuanes, L. (2007). Coronavirus nucleocapsid protein is an RNA chaperone. Virology *357*, 215–227. https://doi.org/10.1016/j.virol.2006.07.046.

40. Zargarian, L., Ai, X., Song, Z.Y., Perez-Gorgol, L., Couprie-Diaz, E., Fossé, P., Xi, X.G., and Mauffret, O. (2025). Chaperone Activity of SARS-CoV-2 Nucleocapsid Protein: RNA Annealing and Destabilization Mechanisms. J. Mol. Biol. *437*, 169312. https://doi.org/10.1016/j.jmb.2025.169312.

41. Bezerra, P.R., and Almeida, F.C.L. (2024). Structural basis for the participation of the SARS-CoV-2 nucleocapsid protein in the template switch mechanism and genomic RNA reorganization. J. Biol. Chem. *300*, 107834. https://doi.org/10.1016/j.jbc.2024.107834.

42. Wang, K., Boysen, C., Shizuya, H., Simon, M.I., and Hood, L. (1997). Complete nucleotide sequence of two generations of a bacterial artificial chromosome cloning vector. Biotechniques *23*, 992–994. https://doi.org/10.2144/97236bm04.

43. DeDiego, M.L., Alvarez, E., Almazán, F., Rejas, M.T., Lamirande, E., Roberts, A., Shieh, W.J., Zaki, S.R., Subbarao, K., and Enjuanes, L. (2007). A severe acute respiratory syndrome coronavirus that lacks the E gene is attenuated in vitro and in vivo. J. Virol. *81*, 1701–1713. https://doi.org/10.1128/JVI.01467-06.

44. Chowrira, B.M., Pavco, P.A., and McSwiggen, J.A. (1994). In vitro and in vivo comparison of hammerhead, hairpin, and hepatitis delta virus self-processing ribozyme cassettes. J. Biol. Chem. *269*, 25856–25864.

45. Pruijssers, A.J., George, A.S., Schäfer, A., Leist, S.R., Gralinksi, L.E., Dinnon, K.H., Yount, B.L., Agostini, M.L., Stevens, L.J., Chappell, J.D., et al. (2020). Remdesivir Inhibits SARS-CoV-2 in Human Lung Cells and Chimeric SARS-CoV Expressing the SARS-CoV-2 RNA Polymerase in Mice. Cell Rep. *32*, 107940. https://doi.org/10.1016/j.celrep.2020.107940.

46. Baliga-Gil, A., Soszynska-Jozwiak, M., Ruszkowska, A., Szczesniak, I., Kierzek, R., Ciechanowska, M., Trybus, M., Jackowiak, P., Peterson, J.M., Moss, W.N., and Kierzek, E. (2024). Targeting sgRNA N secondary structure as a way of inhibiting SARS-CoV-2 replication. Antiviral Res. *228*, 105946. https://doi.org/10.1016/j.antiviral.2024.105946.

47. Soszynska-Jozwiak, M., Ruszkowska, A., Kierzek, R., O'Leary, C.A., Moss, W.N., and Kierzek, E. (2022). Secondary Structure of Subgenomic RNA M of SARS-CoV-2. Viruses *14*, 322. https://doi.org/10.3390/v14020322.

48. Manfredonia, I., Nithin, C., Ponce-Salvatierra, A., Ghosh, P., Wirecki, T.K., Marinus, T., Ogando, N.S., Snijder, E.J., van Hemert, M.J., Bujnicki, J.M., and Incarnato, D. (2020). Genome-wide mapping of SARS-CoV-2 RNA structures identifies therapeutically-relevant elements. Nucleic Acids Res. *48*, 12436–12452. https://doi.org/10.1093/nar/gkaa1053.

49. Zhao, J., Qiu, J., Aryal, S., Hackett, J.L., and Wang, J. (2020). The RNA Architecture of the SARS-CoV-2 3'-Untranslated Region. Viruses *12*, 1473. https://doi.org/10.3390/v12121473.

50. Li, X., Ma, S., and Yi, C. (2016). Pseudouridine: the fifth RNA nucleotide with renewed interests. Curr. Opin. Chem. Biol. *33*, 108–116. https://doi.org/10.1016/j.cbpa.2016.06.014.

51. Pollak, A.J., Zhao, L., and Crooke, S.T. (2023). Systematic Analysis of Chemical Modifications of Phosphorothioate Antisense Oligonucleotides that Modulate Their Innate Immune Response. Nucleic Acid Ther. *33*, 95–107. https://doi.org/10.1089/nat.2022.0067.

52. Valentin, R., Wong, C., Alharbi, A.S., Pradeloux, S., Morros, M.P., Lennox, K.A., Ellyard, J.I., Garcin, A.J., Ullah, T.R., Kusuma, G.D., et al. (2021). Sequence-dependent inhibition of cGAS and TLR9 DNA sensing by 2'-O-methyl gapmer oligonucleotides. Nucleic Acids Res. *49*, 6082–6099. https://doi.org/10.1093/nar/gkab451.

53. Pollak, A.J., Zhao, L., Vickers, T.A., Huggins, I.J., Liang, X.H., and Crooke, S.T. (2022). Insights into innate immune activation via PS-ASO-protein-TLR9 interactions. Nucleic Acids Res. *50*, 8107–8126. https://doi.org/10.1093/nar/gkac618.

54. Hartmann, G. (2023). Cooperative activation of human TLR9 and consequences for the clinical development of antisense and CpG oligodeoxynucleotides. Mol. Ther. Nucleic Acids *34*, 102078. https://doi.org/10.1016/j.omtn.2023.102078.

55. Fitzpatrick, C., Meunier, N., Lepoder, S., Dhorne-Pollet, S., Da Costa, B., Klonjkowki, B., Eléouët, J.F., Burzio, V.A., Delmas, B., and Barrey, E. (2025). Antisense oligonucleotides targeting the SARS-CoV-2 nucleocapsid gene decrease viral titers in hamsters. Mol. Ther. Nucleic Acids *36*, 102612. https://doi.org/10.1016/j.omtn.2025.102612.

56. St-Jean, J.R., Desforges, M., Almazán, F., Jacomy, H., Enjuanes, L., and Talbot, P.J. (2006). Recovery of a neurovirulent human coronavirus OC43 from an infectious cDNA clone. J. Virol. *80*, 3670–3674. https://doi.org/10.1128/JVI.80.7.3670-3674.2006.

57. Almazán, F., González, J.M., Pénzes, Z., Izeta, A., Calvo, E., Plana-Durán, J., and Enjuanes, L. (2000). Engineering the largest RNA virus genome as an infectious bacterial artificial chromosome. Proc. Natl. Acad. Sci. USA *97*, 5516–5521. https://doi.org/10.1073/pnas.97.10.5516.

58. Terada, Y., Kuroda, Y., Morikawa, S., Matsuura, Y., Maeda, K., and Kamitani, W. (2019). Establishment of a Virulent Full-Length cDNA Clone for Type I Feline Coronavirus Strain C3663. J. Virol. *93*, e01208-19. https://doi.org/10.1128/JVI.01208-19.

59. Almazán, F., DeDiego, M.L., Sola, I., Zuñiga, S., Nieto-Torres, J.L., Marquez-Jurado, S., Andrés, G., and Enjuanes, L. (2013). Engineering a replication-competent, propagation-defective Middle East respiratory syndrome coronavirus as a vaccine candidate. mBio *4*, e00650-13. https://doi.org/10.1128/mBio.00650-13.

60. Chen, J., Hu, B.J., Zhao, K., Luo, Y., Lin, H.F., and Shi, Z.L. (2021). Development of A MERS-CoV Replicon Cell Line for Antiviral Screening. Virol. Sin. *36*, 730–735. https://doi.org/10.1007/s12250-020-00341-z.

61. Yang, Y.L., Liang, Q.Z., Xu, S.Y., Mazing, E., Xu, G.H., Peng, L., Qin, P., Wang, B., and Huang, Y.W. (2019). Characterization of a novel bat-HKU2-like swine enteric alphacoronavirus (SeACoV) infection in cultured cells and development of a SeACoV infectious clone. Virology *536*, 110–118. https://doi.org/10.1016/j.virol.2019.08.006.

62. Feng, X., Zhang, X., Jiang, S., Tang, Y., Cheng, C., Krishna, P.A., Wang, X., Dai, J., Zeng, J., Xia, T., and Zhao, D. (2022). A DNA-based non-infectious replicon system to study SARS-CoV-2 RNA synthesis. Comput. Struct. Biotechnol. J. *20*, 5193–5202. https://doi.org/10.1016/j.csbj.2022.08.044.

63. Shizuya, H., Birren, B., Kim, U.J., Mancino, V., Slepak, T., Tachiiri, Y., and Simon, M. (1992). Cloning and stable maintenance of 300-kilobase-pair fragments of human DNA in Escherichia coli using an F-factor-based vector. Proc. Natl. Acad. Sci. USA *89*, 8794–8797. https://doi.org/10.1073/pnas.89.18.8794.

64. Mori, H., Kondo, A., Ohshima, A., Ogura, T., and Hiraga, S. (1986). Structure and function of the F plasmid genes essential for partitioning. J. Mol. Biol. *192*, 1–15. https://doi.org/10.1016/0022-2836(86)90459-6.

65. Heintz, N., and Gong, S. (2020). Working with Bacterial Artificial Chromosomes (BACs) and Other High-Capacity Vectors. Cold Spring Harb. Protoc. *2020*, pdb-top097998. https://doi.org/10.1101/pdb.top097998.

66. Kotaki, T., Xie, X., Shi, P.Y., and Kameoka, M. (2021). A PCR amplicon-based SARS-CoV-2 replicon for antiviral evaluation. Sci. Rep. *11*, 2229. https://doi.org/10.1038/s41598-021-82055-0.

67. Cai, H.L., and Huang, Y.W. (2023). Reverse genetics systems for SARS-CoV-2: Development and applications. Virol. Sin. *38*, 837–850. https://doi.org/10.1016/j.virs.2023.10.001.

68. Wang, X., Zhu, Y., Wu, Q., Jiang, N., Xie, Y., and Deng, Q. (2024). Development of a Cell Culture Model for Inducible SARS-CoV-2 Replication. Viruses *16*, 708. https://doi.org/10.3390/v16050708.

69. Erdmann, M., Wing, P.A.C., Webb, I., Kavanagh Williamson, M., Jearanaiwitayakul, T., Sullivan, E., Bazire, J., Shytaj, I.L., McKeating, J.A., Matthews, D.A., and Davidson, A.D. (2025). A Novel Toolkit of SARS-CoV-2 Sub-Genomic Replicons for Efficient Antiviral Screening. Viruses *17*, 597. https://doi.org/10.3390/v17050597.

70. Kuroda, M., Halfmann, P.J., Uraki, R., Yamayoshi, S., Kim, T., Armbrust, T.A., Spyra, S., Dahn, R., Babujee, L., and Kawaoka, Y. (2025). SARS-CoV-2 virus lacking the envelope and membrane open-reading frames as a vaccine platform. Nat. Commun. *16*, 4453. https://doi.org/10.1038/s41467-025-59533-4.

71. Fischer, H.L., Kline, C., Duprex, W.P., McCarthy, K.R., Watkins, S.C., Conway, J.F., and Ambrose, Z. (2025). Deletion of the Envelope gene attenuates SARS-CoV-2 infection by altered Spike localization and increased cell-to-cell transmission. Preprint at bioRxiv. https://doi.org/10.1101/2025.05.20.655126.

72. Züst, R., Miller, T.B., Goebel, S.J., Thiel, V., and Masters, P.S. (2008). Genetic interactions between an essential 3' cis-acting RNA pseudoknot, replicase gene products, and the extreme 3' end of the mouse coronavirus genome. J. Virol. *82*, 1214–1228. https://doi.org/10.1128/JVI.01690-07.

73. Williams, G.D., Chang, R.Y., and Brian, D.A. (1999). A phylogenetically conserved hairpin-type 3' untranslated region pseudoknot functions in coronavirus RNA replication. J. Virol. *73*, 8349–8355. https://doi.org/10.1128/JVI.73.10.8349-8355.1999.

74. Goebel, S.J., Hsue, B., Dombrowski, T.F., and Masters, P.S. (2004). Characterization of the RNA components of a putative molecular switch in the 3' untranslated region of the murine coronavirus genome. J. Virol. *78*, 669–682. https://doi.org/10.1128/jvi.78.2.669-682.2004.

75. Lin, Y.J., Zhang, X., Wu, R.C., and Lai, M.M. (1996). The 3' untranslated region of coronavirus RNA is required for subgenomic mRNA transcription from a defective interfering RNA. J. Virol. *70*, 7236–7240. https://doi.org/10.1128/JVI.70.10.7236-7240.1996.

76. Jones, S.P., Goossen, C., Lewis, S.D., Delaney, A.M., and Gleghorn, M.L. (2022). Not making the cut: Techniques to prevent RNA cleavage in structural studies of RNase-RNA complexes. J. Struct. Biol. X *6*, 100066. https://doi.org/10.1016/j.yjsbx.2022.100066.

77. Oughtred, R., Rust, J., Chang, C., Breitkreutz, B.J., Stark, C., Willems, A., Boucher, L., Leung, G., Kolas, N., Zhang, F., et al. (2021). The BioGRID database: A comprehensive biomedical resource of curated protein, genetic, and chemical interactions. Protein Sci. *30*, 187–200. https://doi.org/10.1002/pro.3978.

78. Schulten, H.J. (2025). Reviewing the Developing Significance of the Serine Protease PRSS23. Front. Biosci. *30*, 27294. https://doi.org/10.31083/FBL27294.

79. Kretsch, R.C., Xu, L., Zheludev, I.N., Zhou, X., Huang, R., Nye, G., Li, S., Zhang, K., Chiu, W., and Das, R. (2024). Tertiary folds of the SL5 RNA from the 5' proximal region of SARS-CoV-2 and related coronaviruses. Proc. Natl. Acad. Sci. USA *121*, e2320493121. https://doi.org/10.1073/pnas.2320493121.

80. de Moura, T.R., Purta, E., Bernat, A., Martín-Cuevas, E.M., Kurkowska, M., Baulin, E.F., Mukherjee, S., Nowak, J., Biela, A.P., Rawski, M., et al. (2024). Conserved structures and dynamics in 5'-proximal regions of Betacoronavirus RNA genomes. Nucleic Acids Res. *52*, 3419–3432. https://doi.org/10.1093/nar/gkae144.

81. Rafael Ciges-Tomas, J., Franco, M.L., and Vilar, M. (2022). Identification of a guanine-specific pocket in the protein N of SARS-CoV-2. Commun. Biol. *5*, 711. https://doi.org/10.1038/s42003-022-03647-8.

82. Tavares, R.d.C.A., Mahadeshwar, G., Wan, H., Huston, N.C., and Pyle, A.M. (2021). The global and local distribution of RNA structure throughout the SARS-CoV-2 genome. J. Virol. *95*, e02190-20. https://doi.org/10.1128/JVI.02190-20.

83. Khan, D., Terenzi, F., Liu, G., Ghosh, P.K., Ye, F., Nguyen, K., China, A., Ramachandiran, I., Chakraborty, S., Stefan, J., et al. (2023). A viral pan-end RNA element and host complex define a SARS-CoV-2 regulon. Nat. Commun. *14*, 3385. https://doi.org/10.1038/s41467-023-39091-3.

84. Zhu, C., Lee, J.Y., Woo, J.Z., Xu, L., Wrynla, X.H., Yamashiro, L.H., Ji, F., Biering, S.B., Van Dis, E., Gonzalez, F., et al. (2022). An intranasal ASO therapeutic targeting SARS-CoV-2. Nat. Commun. *13*, 4503. https://doi.org/10.1038/s41467-022-32216-0.

85. Vora, S.M., Fontana, P., Mao, T., Leger, V., Zhang, Y., Fu, T.M., Lieberman, J., Gehrke, L., Shi, M., Wang, L., et al. (2022). Targeting stem-loop 1 of the SARS-CoV-2 5' UTR to suppress viral translation and Nsp1 evasion. Proc. Natl. Acad. Sci. USA *119*, e2117198119. https://doi.org/10.1073/pnas.2117198119.

86. Lulla, V., Wandel, M.P., Bandyra, K.J., Ulferts, R., Wu, M., Dendooven, T., Yang, X., Doyle, N., Oerum, S., Beale, R., et al. (2021). Targeting the Conserved Stem Loop 2 Motif in the SARS-CoV-2 Genome. J. Virol. *95*, e0066321. https://doi.org/10.1128/JVI.00663-21.

87. Zhang, K., Zheludev, I.N., Hagey, R.J., Haslecker, R., Hou, Y.J., Kretsch, R., Pintilie, G.D., Rangan, R., Kladwang, W., Li, S., et al. (2021). Cryo-EM and antisense targeting of the 28-kDa frameshift stimulation element from the SARS-CoV-2 RNA genome. Nat. Struct. Mol. Biol. *28*, 747–754. https://doi.org/10.1038/s41594-021-00653-y.

88. Sun, Y., Abriola, L., Niederer, R.O., Pedersen, S.F., Alfajaro, M.M., Silva Monteiro, V., Wilen, C.B., Ho, Y.C., Gilbert, W.V., Surovtseva, Y.V., et al. (2021). Restriction of SARS-CoV-2 replication by targeting programmed -1 ribosomal frameshifting. Proc. Natl. Acad. Sci. USA *118*, e2023051118. https://doi.org/10.1073/pnas.2023051118.

89. Rahman, M.M., Ryan, C.A., Tessier, B.R., and Rozners, E. (2024). Peptide nucleic acids (PNAs) control function of SARS-CoV-2 frameshifting stimulatory element trough PNA-RNA-PNA triplex formation. Heliyon *10*, e33914. https://doi.org/10.1016/j.heliyon.2024.e33914.

90. Wu, R., and Luo, K.Q. (2021). Developing effective siRNAs to reduce the expression of key viral genes of COVID-19. Int. J. Biol. Sci. *17*, 1521–1529. https://doi.org/10.7150/ijbs.59151.

91. Madanagopal, P., Muthukumar, H., and Thiruvengadam, K. (2022). Computational study and design of effective siRNAs to silence structural proteins associated genes of Indian SARS-CoV-2 strains. Comput. Biol. Chem. *98*, 107687. https://doi.org/10.1016/j.compbiolchem.2022.107687.

92. Klock, H.E., and Lesley, S.A. (2009). The Polymerase Incomplete Primer Extension (PIPE) method applied to high-throughput cloning and site-directed mutagenesis. Methods Mol. Biol. *498*, 91–103. https://doi.org/10.1007/978-1-59745-196-3_6.

93. Xia, T., SantaLucia, J., Jr., Burkard, M.E., Kierzek, R., Schroeder, S.J., Jiao, X., Cox, C., and Turner, D.H. (1998). Thermodynamic parameters for an expanded nearest-neighbor model for formation of RNA duplexes with Watson-Crick base pairs. Biochemistry *37*, 14719–14735. https://doi.org/10.1021/bi9809425.

94. Kierzek, E., and Kierzek, R. (2003). The thermodynamic stability of RNA duplexes and hairpins containing N6-alkyladenosines and 2-methylthio-N6-alkyladenosines. Nucleic Acids Res. *31*, 4472–4480. https://doi.org/10.1093/nar/gkg633.

95. Kierzek, E., Zhang, X., Watson, R.M., Kennedy, S.D., Szabat, M., Kierzek, R., and Mathews, D.H. (2022). Secondary structure prediction for RNA sequences including N(6)-methyladenosine. Nat. Commun. *13*, 1271. https://doi.org/10.1038/s41467-022-28817-4.

OMTN, Volume *37*

Supplemental information

Bridging the antiviral gap: A BAC-based non-infectious SARS-CoV-2 replicon reveals potent antisense gapmers

Agnieszka Ruszkowska, Agnieszka Baliga-Gil, Julita Piasecka, Maria Ciechanowska, Ryszard Kierzek, and Elzbieta Kierzek

SUPPLEMENTAL FIGURES

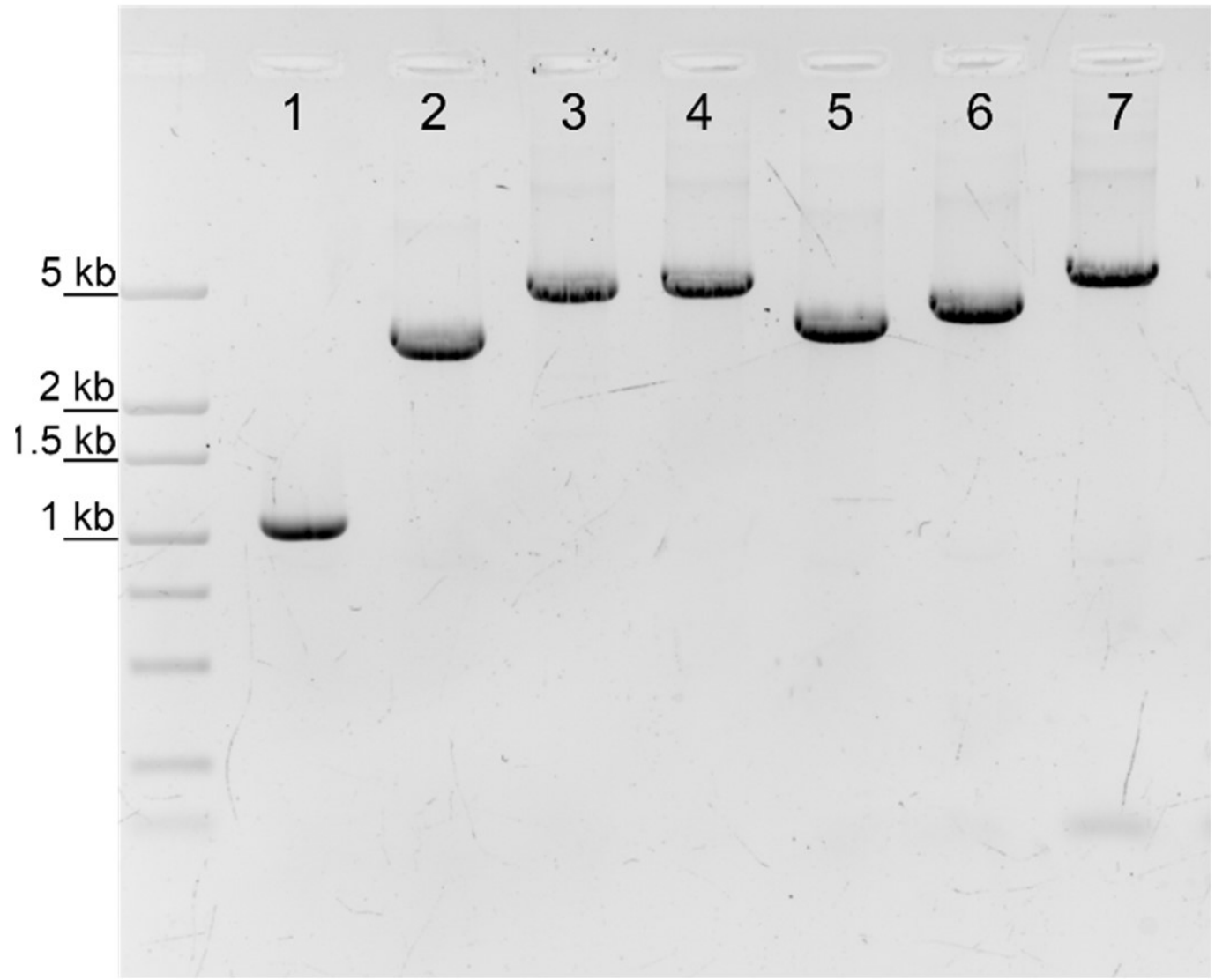

Figure S1. DNA fragments F1-F7 used to assembly pBAC-SARS-CoV-2_*S/eGFP*_*ΔE* non-infectious replicon. Length of the PCR amplified fragments: F1 – 1064 bp; F2 – 3150 bp; F3 – 5122 bp; F4 – 5405 bp; F5 – 3539 bp; F6 – 4131 bp; F7 – 5741 bp.

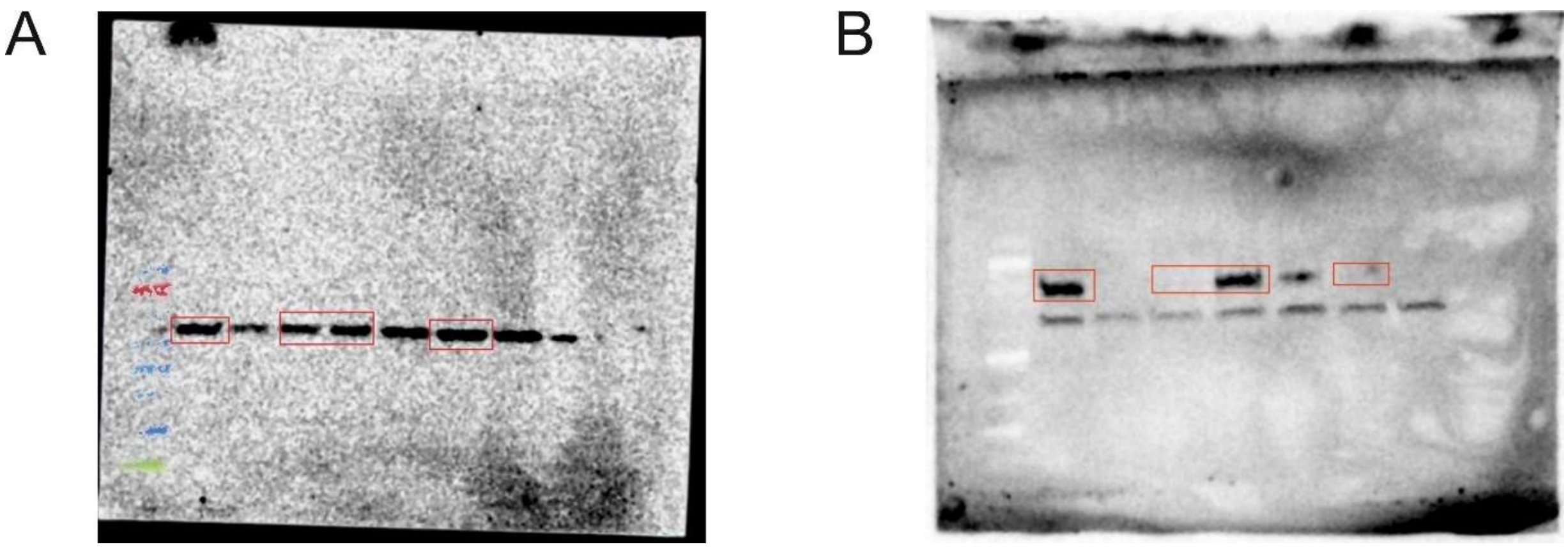

Figure S2. Uncropped Western blot images. Panel A shows the membrane probed for actin, and Panel B shows the membrane probed for the nucleocapsid protein. Red rectangles indicate the bands presented in Figure 2.

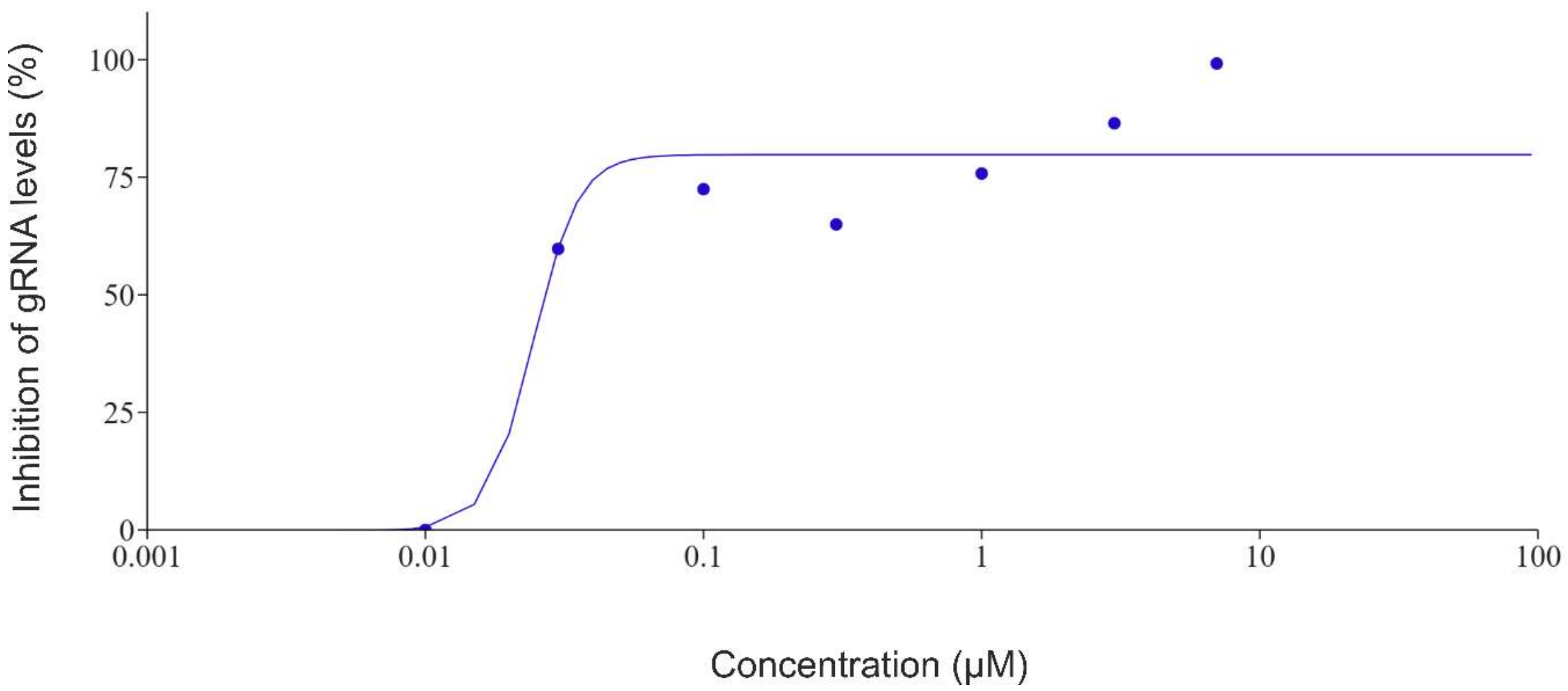

Figure S3. Dose-dependent inhibition of the pBAC-SARS-CoV-2_*S/eGFP_ΔE* replicon activity by remdesivir. Effect of remdesivir on replicon activity was evaluated at concentrations: 0.01, 0.03, 0.1, 0.3, 1, 3, and 2 μM. Replicon activity was quantified by RT-qPCR. The EC_{50} value was calculated using the AAT Bioquest online calculator.

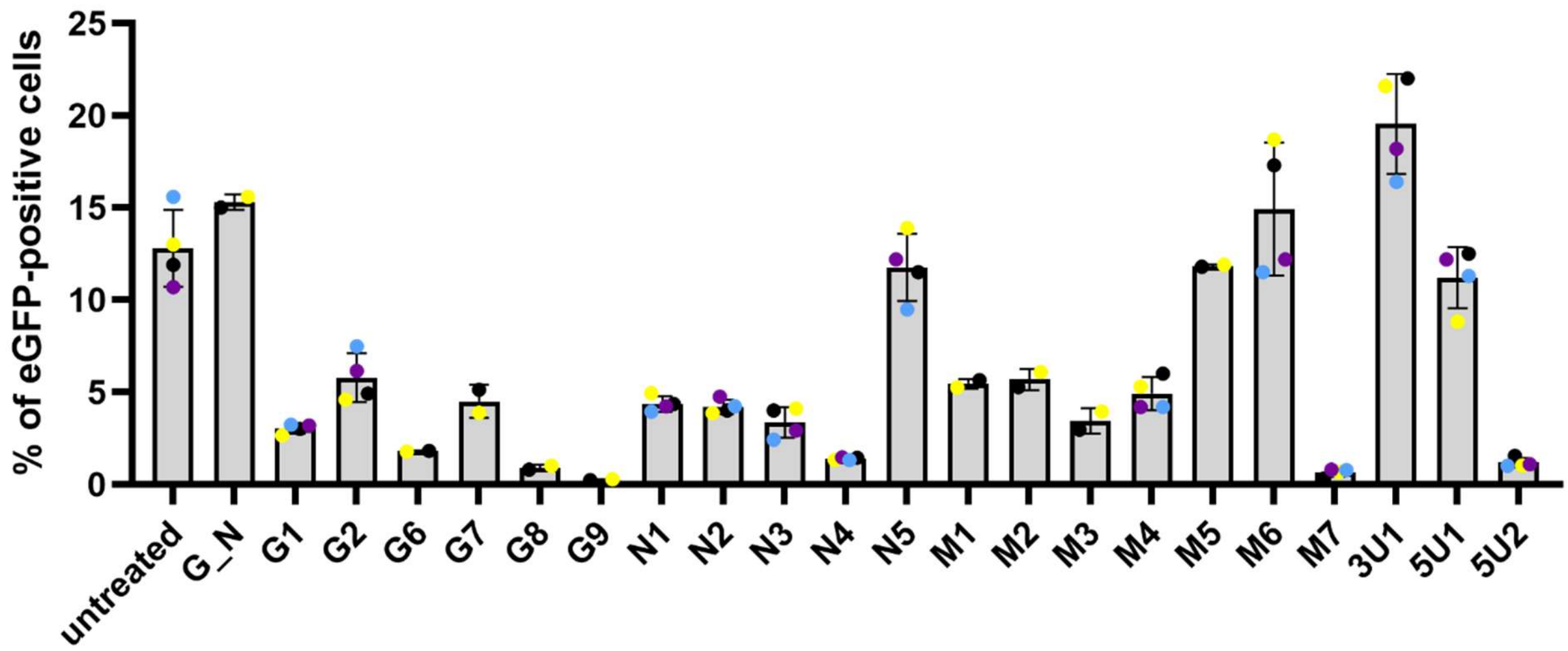

Figure S4. Effect of simultaneous co-transfection with gapmers and the pBAC-SARS-CoV-2_*S/eGFP_ΔE* replicon on replicon activity, assessed by the eGFP signal. Replicon activity is shown as the percentage of eGFP-positive cells among viable cells. Colored dots indicate individual experimental replicates, whereas gray bars represent the mean of all replicates.

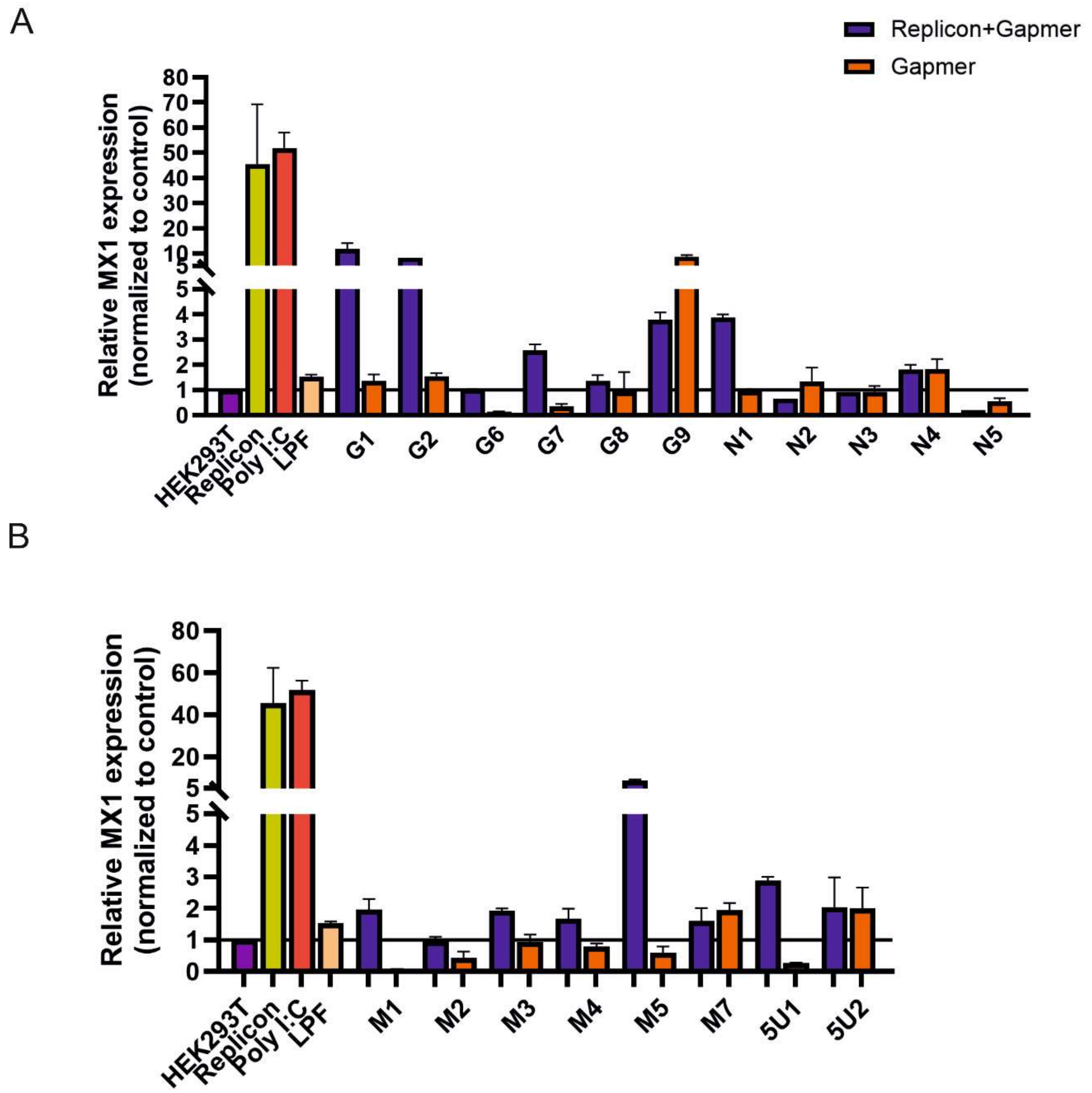

Figure S5. Expression of the interferon-stimulated gene *MX1* in HEK293T cells co-transfected with the replicon and gapmers. Bars represent the mean ± SD from two biological replicates, each with two technical replicates. Blue bars represent HEK293T cells treated with both the replicon and gapmer, whereas orange bars represent cells treated with gapmer only. Gene expression was quantified by qPCR analysis. Panel A shows results for gapmers targeting the *N* gene, whereas panel B shows results for gapmers targeting the *M* gene and UTRs. Abbreviations: Poly I:C, polyinosinic:polycytidylic acid; LPF, Lipofectamine.

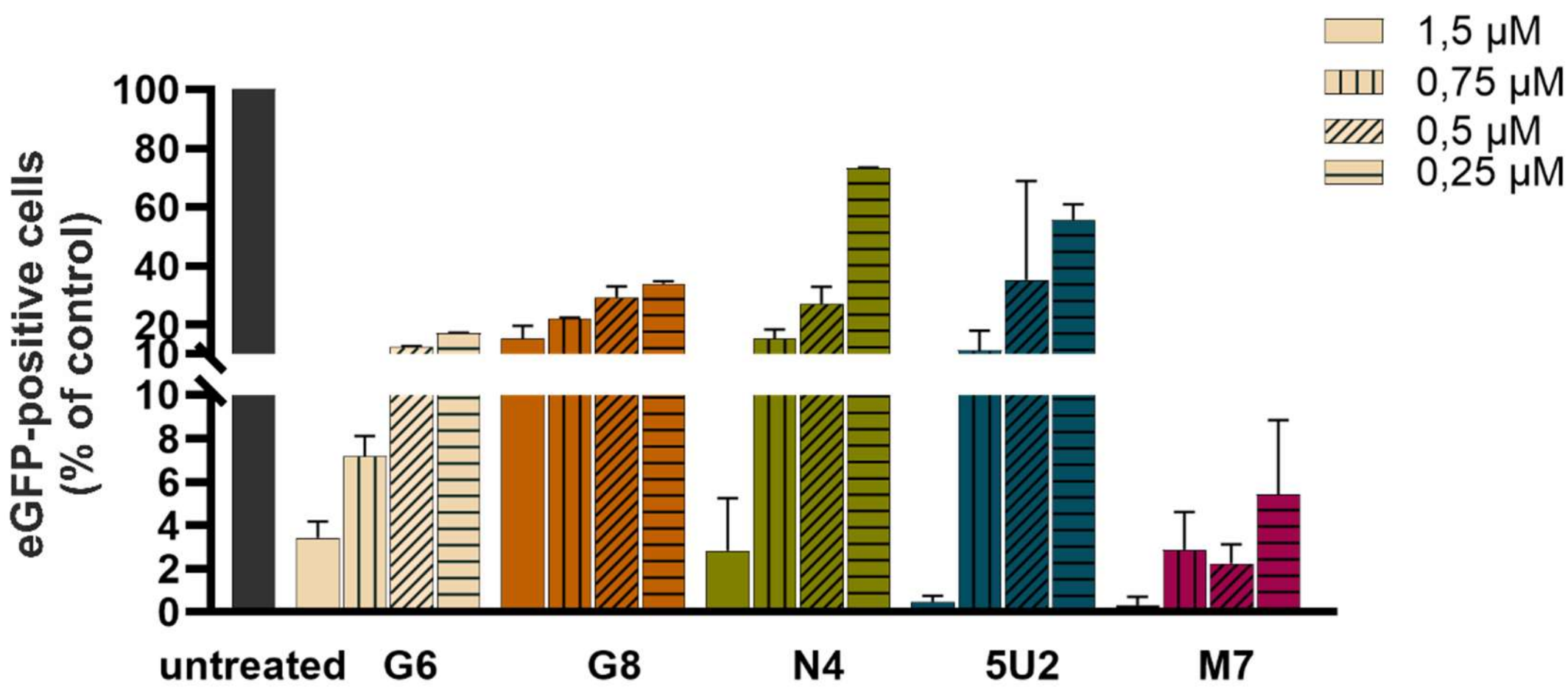

Figure S6. Dose-dependent inhibition of the pBAC-SARS-CoV-2_*S/eGFP_ΔE* replicon activity by selected gapmers. Replicon activity was quantified by flow cytometry as the percentage of eGFP-positive cells. HEK293T cells transfected with the replicon alone served as the reference control and were assigned a value of 100%. Bars represent the mean ± SD from two biological replicates, each with two technical replicates.

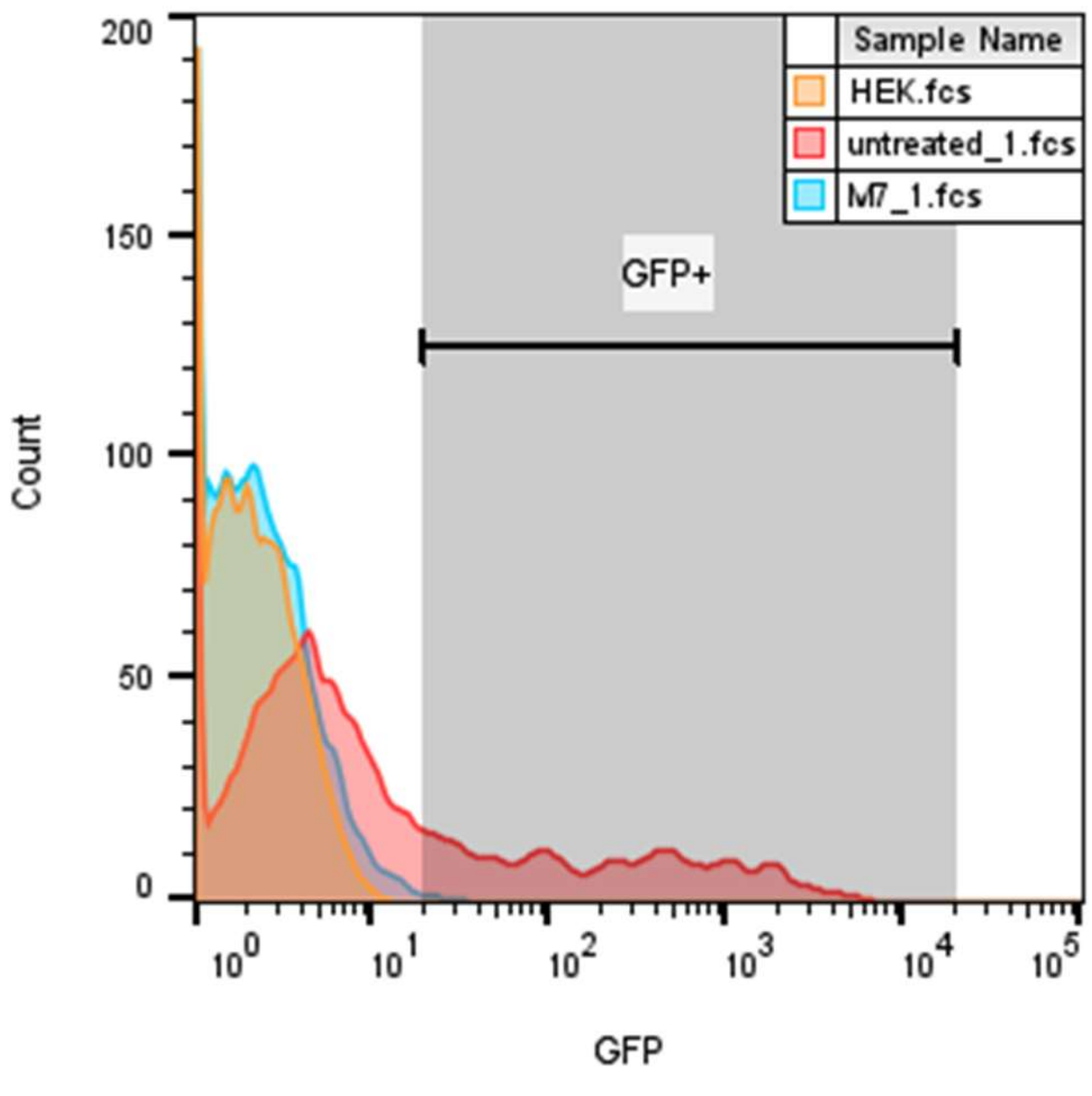

Sample Name	GFP+ Median
HEK.fcs	n/a
M7_1.fcs	31,4
untreated_1.fcs	210

Figure S7. Flow cytometry histogram showing eGFP intensity in HEK293T cells transfected with the pBAC-SARS-CoV-2_*S/eGFP_ΔE* replicon. eGFP intensity is shown for cells transfected with the replicon alone (untreated) and cells co-transfected with the replicon and gapmer M7.

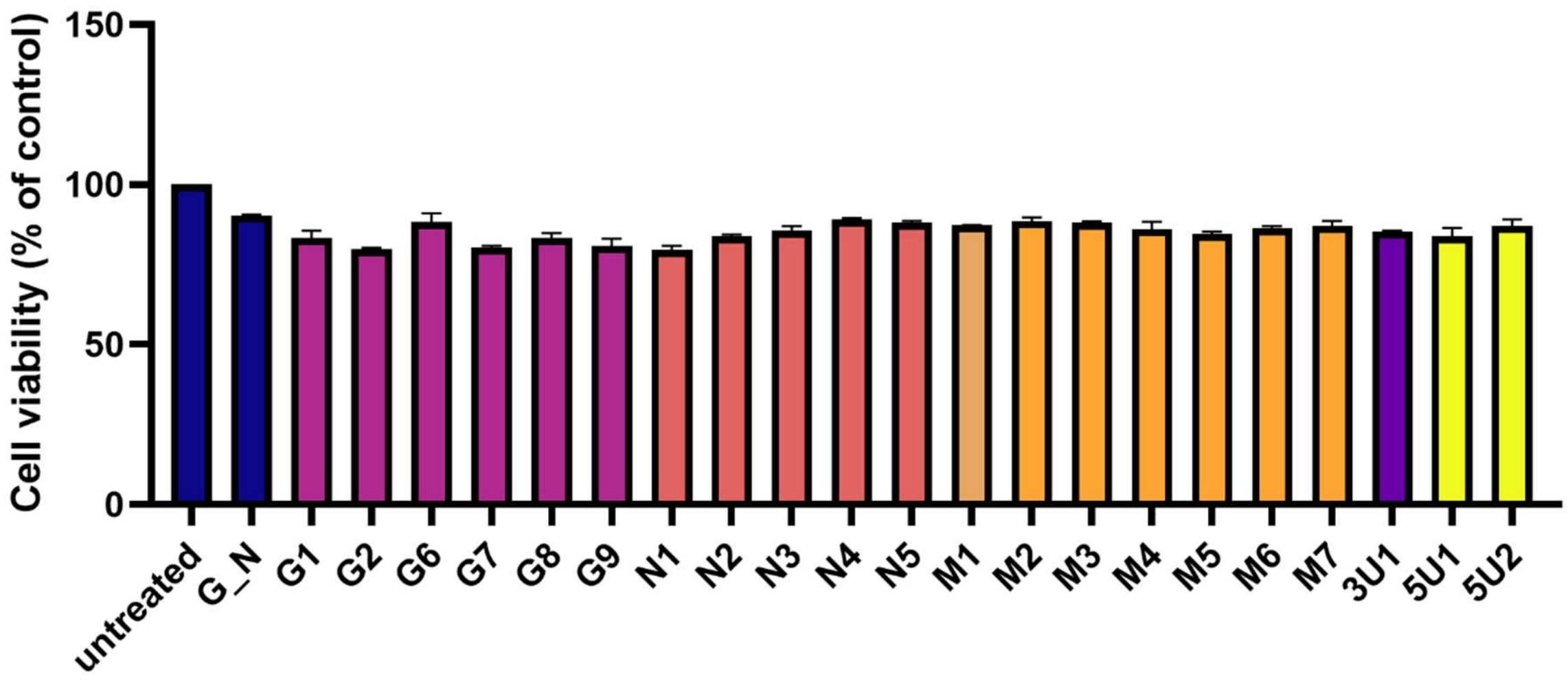

Figure S8. Viability of HEK293T cells co-transfected with the replicon and gapmers at a final gapmer concentration of 0.75 μM. The quantities of dead cells stained with propidium iodide (PI) were measured by flow cytometry, then the cell viability was analyzed based on these measurements. Experiment was performed in two biological replicates.

SUPPLEMENTAL TABLES

Table S1. Sequence of F7.1 and F7.2 used for the assembly of F7.

F7.1	AGCAACCAATGGAGATTGATTAAACGAACATGAAAATTATTCTTTTCTTGGCACTGATAACACTCGCTACTTGTGAGCTTTATCACTACCAAGAGTGTGTTAGAGGT<br>ACAACAGTACTTTTAAAAGAACCTTGCTCTTCTGGAACATACGAGGGCAATTCACCATTTCATCCTCTAGCTGATAACAAATTTGCACTGACTTGCTTTAGCACTCAA<br>TTTGCTTTTGCTTGTCCTGACGGCGTAAAACACGTCTATCAGTTACGTGCCAGATCAGTTTCACCTAAACTGTTCATCAGACAAGAGGAAGTTCAAGAACTTTACTCT<br>CCAATTTTTCTTATTGTTGCGGCAATAGTGTTTATAACACTTTGCTTCACACTCAAAAGAAAGACAGAATGATTGAACTTTCATTAATTGACTTCTATTTGTGCTTTTT<br>AGCCTTTCTGCTATTCCTTGTTTTAATTATGCTTATTATCTTTTGGTTCTCACTTGAACTGCAAGATCATAATGAAACTTGTCACGCCTAAACGAACATGAAATTTCTT<br>GTTTTCTTAGGAATCATCACAACTGTAGCTGCATTTCACCAAGAATGTAGTTTACAGTCATGTACTCAACATCAACCATATGTAGTTGATGACCCGTGTCCTATTCAC<br>TTCTATTCTAAATGGTATATTAGAGTAGGAGCTAGAAAATCAGCACCTTTAATTGAATTGTGCGTGGATGAGGCTGGTTCTAAATCACCCATTCAGTACATCGATATC<br>GGTAATTATACAGTTTCCTGTTTACCTTTTACAATTAATTGCCAGGAACCTAAATTGGGTAGTCTTGTAGTGCGTTGTTCGTTCTATGAAGACTTTTTAGAGTATCATG<br>ACGTTCGTGTTGTTTTAGATTTCATCTAAACGAACAAACTAAAATGTCTGATAATGGACCCCAAAATCAGCGAAATGCACCCCGCATTACGTTTGGTGGACCCTCAG<br>ATTCAACTGGCAGTAACCAGAATGGAGAACGCAGTGGGGCGCGATCAAAACAACGTCGGCCCCAAGGTTTACCCAATAATACTGCGTCTTGGTTCACCGCTCTCACT<br>CAACATGGCAAGGAAGACCTTAAATTCCCTCGAGGACAAGGCGTTCCAATTAACACCAATAGCAGTCCAGATGACCAAATTGGCTACTACCGAAGAGCTACCAGAC<br>GAATTCGTGGTGGTGACGGTAAAATGAAAGATCTCAGTCCAAGATGGTATTTCTACTACCTAGGAACTGGGCCAGAAGCTGGACTTCCCTATGGTGCTAACAAAGAC<br>GGCATCATATGGGTTGCAACTGAGGGAGCCTTGAATACACCAAAAGATCACATTGGCACCCGCAATCCTGCTAACAATGCTGCAATCGTGCTACAACTTCCTCAAGG<br>AACAACATTGCCAAAAGGCTTCTACGCAGAAGGGAGCAGAGGCGGCAGTCAAGCCTCTTCTCGTTCCTCATCACGTAGTCGCAACAGTTCAAGAAATTCAACTCCA<br>GGCAGCAGTAGGGGAACTTCTCCTGCTAGAATGGCTGGCAATGGCGGTGATGCTGCTCTTGCTTTGCTGCTGCTTGACAGATTGAACCAGCTTGAGAGCAAAATGTC<br>TGGTAAAGGCCAACAACAACAAGGCCAAACTGTCACTAAGAAATCTGCTGCTGAGGCTTCTAAGAAGCCTCGGCAAAAACGTACTGCCACTAAAGCATACAATGTA<br>ACACAAGCTTTCGGCAGACGTGGTCCAGAACAAACCCAAGGAAATTTTGGGGACCAGGAACTAATCAGACAAGGAACTGATTACAAACATTGGCCGCAAATTGCAC<br>AATTTGCCCCCAGCGCTTCAGCGTTCTTCGGAATGTCGCGCATTGGCATGGAAGTCACACCTTCGGGAACGTGGTTGACCTACACAGGTGCCATCAAATTGGATGAC<br>AAAGATCCAAATTTCAAAGATCAAGTCATTTTGCTGAATAAGCATATTGACGCATACAAAACATTCCCACCAACAGAGCCTAAAAAGGACAAAAAGAAGAAGGCTG<br>ATGAAACTCAAGCCTTACCGCAGAGACAGAAGAAACAGCAAACTGTGACTCTTCTTCCTGCTGCAGATTTGGATGATTTCTCCAAACAATTGCAACAATCCATGAGC<br>AGTGCTGACTCAACTCAGGCCTAAACTCATGCAGACCACACAAGGCAGATGGGCTATATAAACGTTTTCGCTTTTCCGTTTACGATATATAGTCTACTCTTGTGCAGA<br>ATGAATTCTCGTAACTACATAGCACAAGTAGATGTAGTTAACTTTAATCTCACATAGCAATCTTTAATCAGTGTGTAACATTAGGGAGGACTTGAAAGAGCCACCAC<br>ATTTTCACCGAGGCCACGCGGAGTACGATCGAGTGTACAGTGAACAATGCTAGGGAGAGCTGCCTATATGGAAGAGCCCTAATGTGTAAAATTAATTTTAGTAGTGC<br>TATCCCCATGTGATTTTAATAGCTTCTTAGGAGAATGACAAAAAAAAAGGGTCGGCATGGCATCTCCACCTCCTCGCGGTCCGACCTGGGCTACTTCGGTAGGCTAA<br>GGGAGAAGCTGTGCCTTCTAGTTGCCAGCCATCTGTTGTTTGCCCCTCCCCCGTGCCTTCCTTGACCCTGGAAGGTGCCACTCCCACTGTCCTTTCCTAATAAAATGA<br>GGAAATTGCATCGCATTGTCTGAGTAGGTGTCATTCTATTCTGGGGGGTGGGGTGGGGCAGGACAGCAAGGGGGAGGATTGGGAAGACAATAGCAGGCATGCTGGG<br>GATGCGGTGGGCTCTATGGGGCGCGCCAAGCTTGAGTATTCTATAGTGTCACCTAAATAGCTTGGCGTAATCATGGTCATAGCTGTTTCCTGTGTGAAATTGTTATCC<br>GCTCACAATTC

F7.2	AAGGTCAAATCAATGATATGATTTTATCTCTTCTTAGTAAAGGTAGACTTATAATTAGAGAAAACAACAGAGTTGTTATTTCTAGTGATGTTCTTGTTAACAACTAAA<br>CGAACAATGTTTGTTTTTCTTGTTTTATTGCCACTAgcgatcgcaATGGTGAGCAAGGGCGAGGAGCTGTTCACCGGGGTGGTGCCCATCCTGGTCGAGCTGGACGGCGAC<br>GTAAACGGCCACAAGTTCAGCGTGTCCGGCGAGGGCGAGGGCGATGCCACCTACGGCAAGCTGACCCTGAAGTTCATCTGCACCACCGGCAAGCTGCCCGTGCCCT<br>GGCCCACCCTCGTGACCACCCTGACCTACGGCGTGCAGTGCTTCAGCCGCTACCCCGACCACATGAAGCAGCACGACTTCTTCAAGTCCGCCATGCCCGAAGGCTAC<br>GTCCAGGAGCGCACCATCTTCTTCAAGGACGACGGCAACTACAAGACCCGCGCCGAGGTGAAGTTCGAGGGCGACACCCTGGTGAACCGCATCGAGCTGAAGGGC<br>ATCGACTTCAAGGAGGACGGCAACATCCTGGGGCACAAGCTGGAGTACAACTACAACAGCCACAACGTCTATATCATGGCCGACAAGCAGAAGAACGGCATCAAG<br>GTGAACTTCAAGATCCGCCACAACATCGAGGACGGCAGCGTGCAGCTCGCCGACCACTACCAGCAGAACACCCCCATCGGCGACGGCCCCGTGCTGCTGCCCGACA<br>ACCACTACCTGAGCACCCAGTCCGCCCTGAGCAAAGACCCCAACGAGAAGCGCGATCACATGGTCCTGCTGGAGTTCGTGACCGCCGCCGGGATCACTCTCGGCAT<br>GGACGAGCTGTACAAGTAATGTAGTTGTCTCAAGGGCTGTTGTTCTTGTGGATCCTGCTGCAAATTTGATGAAGACGACTCTGAGCCAGTGCTCAAAGGAGTCAAAT<br>TACATTACACATAAACGAACTTATGGATTTGTTTATGAGAATCTTCACAATTGGAACTGTAACTTTGAAGCAAGGTGAAATCAAGGATGCTACTCCTTCAGATTTTGT<br>TCGCGCTACTGCAACGATACCGATACAAGCCTCACTCCCTTTCGGATGGCTTATTGTTGGCGTTGCACTTCTTGCTGTTTTTCATAGCGCTTCCAAAATCATAACCCTC<br>AAAAAGAGATGGCAACTAGCACTCTCCAAGGGTGTTCACTTTGTTTGCAACTTGCTGTTGTTGTTTGTAACAGTTTACTCACACCTTTTGCTCGTTGCTGCTGGCCTTG<br>AAGCCCCTTTTCTCTATCTTTATGCTTTAGTCTACTTCTTGCAGAGTATAAACTTTGTAAGAATAATAATGAGGCTTTGGCTTTGCTGGAAATGCCGTTCCAAAAACCC<br>ATTACTTTATGATGCCAACTATTTTCTTTGCTGGCATACTAATTGTTACGACTATTGTATACCTTACAATAGTGTAACTTCTTCAATTGTCATTACTTCAGGTGATGGC<br>ACAACAAGTCCTATTTCTGAACATGACTACCAGATTGGTGGTTATACTGAAAAATGGGAATCTGGAGTAAAAGACTGTGTTGTATTACACAGTTACTTCACTTCAGA<br>CTATTACCAGCTGTACTCAACTCAATTGAGTACAGACACTGGTGTTGAACATGTTACCTTCTTCATCTACAATAAAATTGTTGATGAGCCTGAAGAACATGTCCAAAT<br>TCACACAATCGACGGTTCATCCGGAGTTGTTAATCCAGTAATGGAACCAATTTATGATGAACCGACGACGACTACTAGCGTGCCTTTGTAAGCACAAGCTGATGAGT<br>ACCAATTTGTGTACTCATTCGTTTCGGAAGAGACAGGTACGTTAAACTCTCGTGTTAAAAATCTGAATTCTTCTAGAGTTCCTGATCTTCTGGTCTAAACGAACTAAA<br>TATTATATTAGTTTTTCTGTTTGGAACTTTAATTTTAGCCATGGCAGATTCCAACGGTACTATTACCGTTGAAGAGCTTAAAAAGCTCCTTGAACAATGGAACCTAGT<br>AATAGGTTTCCTATTCCTTACATGGATTTGTCTTCTACAATTTGCCTATGCCAACAGGAATAGGTTTTTGTATATAATTAAGTTAATTTTCCTCTGGCTGTTATGGCCA<br>GTAACTTTAGCTTGTTTTGTGCTTGCTGCTGTTTACAGAATAAATTGGATCACCGGTGGAATTGCTATCGCAATGGCTTGTCTTGTAGGCTTGATGTGGCTCAGCTACT<br>TCATTGCTTCTTTCAGACTGTTTGCGCGTACCCGTTCCATGTGGTCATTCAATCCAGAAACTAACATTCTTCTCAACGTGCCACTCCATGGCACTATTCTGACCAGACC<br>GCTTCTAGAAAGTGAACTCGTAATCGGAGCTGTGATCCTTCGTGGACATCTTCGTATTGCTGGACACCATCTAGGACGCTGTGACATCAAGGACCTGCCTAAAGAAA<br>TCACTGTTGCTACATCACGAACGCTTTCTTATTACAAATTGGGAGCTTCGCAGCGTGTAGCAGGTGACTCAGGTTTTGCTGCATACAGTCGCTACAGGATTGGCAACT<br>ATAAATTAAACACAGACCATTCCAGTAGCAGTGACAATATTGCTTTGCTTGTACAGTAAGTGACAACAGATGTTTCATCTCGTTGACTTTCAGGTTACTATAGCAGAG<br>ATATTACTAATTATTATGAGGACTTTTAAAGTTTCCATTTGGAATCTTGATTACATCATAAACCTCATAATTAAAAATTTATCTAAGTCACTAACTGAGAATAAATAT<br>TCTCAATTAGATGAAGAGCAACCAATGGAGATTGATTAAACGAACATGAAAATTATTCTTTTCTTGGCACTGATAACACTCGCTACTTGTGAGCTTTATCACTACCAA<br>GAGTGTGTTAGAGGTACAACA

Table S2. Characteristics of the gapmers.

Name	Sequence	Target	gRNA (nt numbers)	sgRNA (nt numbers)	Decrease of gRNA level (%)	Decrease of sgRNA level (%)	Decrease of the number of eGFP+ cells (%)	Decrease of eGFP intensity
G_N	$\mathrm{U}^{PS,L}\mathrm{U}^{PS,M}\mathrm{C}^{PS,L}A^{PS}A^{PS}C^{PS}G^{PS}A^{PS}G^{PS}A^{PS}C^{PS}\mathrm{U}^{PS,L}\mathrm{U}^{PS,M}\mathrm{C}^{PS,L}$	non-complimentary	-	-	-	-	-	
G1	$\mathrm{G}^{PS,L}\mathrm{A}^{PS,M}\mathrm{C}^{PS,L}G^{PS}T^{PS}T^{PS}G^{PS}T^{PS}T^{PS}T^{PS}T^{PS}G^{PS}\mathrm{A}^{PS,L}\mathrm{U}^{PS,M}\mathrm{C}^{PS,L}$	N	28380-28394	190-204	16	96	76	71
G2	$\mathrm{U}^{PS,L}\mathrm{G}^{PS,M}\mathrm{G}^{PS,L}A^{PS}G^{PS}A^{PS}A^{PS}A^{PS}T^{PS}C^{PS}A^{PS}\mathrm{U}^{PS,L}\mathrm{C}^{PS,M}\mathrm{C}^{PS,L}$	N	29473-29486	1283-1296	38	95	55	30
G6	$\mathrm{U}^{PS,L}\mathrm{U}^{PS,M}\mathrm{G}^{L,PS}C^{PS}C^{PS}G^{PS}A^{PS}G^{PS}G^{PS}C^{PS}T^{PS}\mathrm{U}^{PS,L}\mathrm{C}^{PS,M}\mathrm{U}^{PS,L}$	N	29040-29053	850-863	96	91	86	41
G7	$\mathrm{C}^{PS,L}\mathrm{U}^{PS,M}\mathrm{A}^{PS,L}C^{PS}T^{PS}G^{PS}C^{PS}T^{PS}G^{PS}C^{PS}C^{PS}\mathrm{U}^{PS,L}\mathrm{G}^{PS,M}\mathrm{G}^{PS,L}$	N	28868-28881	678-691	75	80	70	↑
G8	$\mathrm{U}^{PS,L}\mathrm{C}^{PS,M}\mathrm{A}^{PS,L}A^{PS}G^{PS}C^{PS}A^{PS}G^{PS}C^{PS}A^{PS}G^{PS}\mathrm{C}^{PS,L}\mathrm{A}^{PS,M}\mathrm{A}^{PS,L}$	N	28934-28947	744-757	91	93	93	51
G9	$\mathrm{A}^{PS,L}\mathrm{U}^{PS,M}\mathrm{C}^{PS,L}G^{PS}C^{PS}G^{PS}C^{PS}C^{PS}C^{PS}C^{PS}A^{PS}\mathrm{C}^{PS,L}\mathrm{U}^{PS,M}\mathrm{G}^{PS,L}$	N	28369-28382	179-192	52	99	98	60
N1	$\mathrm{C}^{PS,L}\mathrm{A}^{PS,M}\mathrm{U}^{PS,L}T^{PS}A^{PS}T^{PS}C^{PS}A^{PS}G^{PS}A^{PS}C^{PS}A^{PS}T^{PS}T^{PS}T^{PS}T^{PS}A^{PS}G^{PS}T^{PS}\mathrm{U}^{PS,L}\mathrm{U}^{PS,M}\mathrm{G}^{PS,L}$	N	28265-28286	75-96	52	98	63	70
N2	$\mathrm{C}^{PS,L}\mathrm{U}^{PS,M}\mathrm{U}^{PS,L}T^{PS}T^{PS}G^{PS}G^{PS}T^{PS}G^{PS}T^{PS}A^{PS}\mathrm{U}^{PS,L}\mathrm{U}^{PS,M}\mathrm{C}^{PS,L}$	N	28691-28703	501-513	58	98	64	61
N3	$\mathrm{U}^{PS,L}\mathrm{U}^{PS,M}\mathrm{C}^{PS,L}T^{PS}T^{PS}G^{PS}A^{PS}A^{PS}C^{PS}T^{PS}G^{PS}\mathrm{U}^{PS,L}\mathrm{U}^{PS,M}\mathrm{G}^{PS,L}$	N	28846-28859	656-669	41	98	71	61
N4	$\mathrm{G}^{PS,L}\mathrm{A}^{PS,M}\mathrm{U}^{PS,L}C^{PS}T^{PS}T^{PS}T^{PS}G^{PS}T^{PS}C^{PS}A^{PS}\mathrm{U}^{PS,L}\mathrm{C}^{PS,M}\mathrm{C}^{PS,L}$	N	29290-29303	1100-1113	97	98	87	69
N5	$\mathrm{U}^{PS,L}\mathrm{U}^{PS,M}\mathrm{G}^{PS,L}A^{PS}G^{PS}T^{PS}T^{PS}T^{PS}C^{PS}A^{PS}T^{PS}\mathrm{C}^{PS,L}\mathrm{A}^{PS,M}\mathrm{G}^{PS,L}$	N	29400-29413	1210-1223	38	92	-	33
3U1	$\mathrm{U}^{PS,L}\mathrm{A}^{PS,M}\mathrm{A}^{PS,L}C^{PS}T^{PS}A^{PS}C^{PS}A^{PS}T^{PS}C^{PS}T^{PS}A^{PS}C^{PS}T^{PS}T^{PS}G^{PS}\mathrm{U}^{PS,L}\mathrm{G}^{PS,M}\mathrm{C}^{PS,L}$	3' UTR	29639-29657	1482-1500	↑	(N;M)↑	↑	1
5U1	$\mathrm{G}^{PS,L}\mathrm{A}^{PS,M}\mathrm{A}^{PS,L}A^{PS}G^{PS}T^{PS}T^{PS}G^{PS}G^{PS}T^{PS}T^{PS}\mathrm{G}^{PS,L}\mathrm{G}^{PS,M}\mathrm{U}^{PS,L}$	5'UTR	31-44	31-44	56	95(N);94(M)	10	33
5U2	$\mathrm{C}^{PS,L}\mathrm{A}^{PS,M}\mathrm{C}^{PS,L}G^{PS}G^{PS}A^{PS}C^{PS}G^{PS}A^{PS}A^{PS}A^{PS}\mathrm{C}^{PS,L}\mathrm{C}^{PS,M}\mathrm{G}^{PS,L}$	5'UTR	197-210	-	95	92(N);94(M)	90	74
M1	$\mathrm{U}^{PS,L}\mathrm{U}^{PS,M}\mathrm{U}^{PS,L}A^{PS}G^{PS}T^{PS}T^{PS}C^{PS}G^{PS}T^{PS}T^{PS}T^{PS}A^{PS}\mathrm{G}^{PS,L}\mathrm{A}^{PS,M}\mathrm{G}^{PS,L}$	M	-	64-79	75	85	76	83

M2	$\mathrm{U}^{\mathrm{PS,L}}\mathrm{U}^{\mathrm{PS,M}}\mathrm{A}^{\mathrm{PS,L}}C^{PS}T^{PS}A^{PS}G^{PS}G^{PS}T^{PS}T^{PS}C^{PS}C^{PS}A^{PS}\mathrm{U}^{\mathrm{PS,L}}\mathrm{U}^{\mathrm{PS,M}}\mathrm{G}^{\mathrm{L,PS}}$	M	26577-26592	174-189	89	60	77	74
M3	$^{\mathrm{PP}}\mathrm{A}^{\mathrm{PS,L}}C^{PS}T^{PS}A^{PS}G^{PS}G^{PS}T^{PS}T^{PS}C^{PS}C^{PS}A^{\mathrm{PP}}\mathrm{G}^{\mathrm{PS,L}}$	M	26577-26592	174-189	87	59	86	79
M4	$\mathrm{C}^{\mathrm{PS,L}}\mathrm{C}^{\mathrm{PS,M}}\mathrm{U}^{\mathrm{PS,L}}G^{PS}T^{PS}T^{PS}G^{PS}G^{PS}C^{PS}A^{PS}T^{PS}A^{PS}\mathrm{G}^{\mathrm{PS,}}\mathrm{L}\mathrm{G}^{\mathrm{PS,M}}\mathrm{C}^{\mathrm{PS,L}}$	M	26634-26648	231-245	69	81	75	75
M5	$\mathrm{G}^{\mathrm{PS,L}}\mathrm{U}^{\mathrm{PS,M}}\mathrm{C}^{\mathrm{PS,L}}T^{PS}G^{PS}A^{PS}A^{PS}A^{PS}G^{PS}A^{PS}A^{PS}G^{PS}C^{PS}\mathrm{A}^{\mathrm{PS,L}}\mathrm{A}^{\mathrm{PS,M}}\mathrm{U}^{\mathrm{PS,L}}$	M	26811-26826	408-423	20	67	46	79
M6	$\mathrm{U}^{\mathrm{PS,L}}\mathrm{G}^{\mathrm{PS,M}}\mathrm{U}^{\mathrm{PS,L}}G^{PS}T^{PS}T^{PS}T^{PS}A^{PS}A^{PS}T^{PS}T^{PS}T^{PS}\mathrm{A}^{\mathrm{PS,L}}\mathrm{U}^{\mathrm{PS,M}}\mathrm{A}^{\mathrm{PS,L}}$	M	27132-27146	729-743	↑	25	15	61
M7	$\mathrm{G}^{\mathrm{PS,L}}\mathrm{G}^{\mathrm{PS,M}}\mathrm{U}^{\mathrm{PS,L}}A^{PS}A^{PS}T^{PS}A^{PS}G^{PS}T^{PS}A^{PS}C^{PS}C^{PS}G^{PS}\mathrm{U}^{\mathrm{PS,L}}\mathrm{U}^{\mathrm{PS,M}}\mathrm{G}^{\mathrm{PS,L}}$	M	26534-26549	131-146	92	97	90	82
Modifications of nucleotides (N): N^{PS}-phosphorothioate, N^{M}-2′O-methylated RNA, N^{L}-locked nucleic acid, N^{PP}-pseudouridine.								

Table S3. Sequence conservation of the gapmers G6, G8, N4, M7, and 5U2 target sites across major SARS-CoV-2 variants. SARS-CoV-2 sequences were obtained from the Nextstrain database.

**Name**					**G6**	**G8**	**N4**	**M7**	**5U2**
**Sequence**					TTGCCGAGGCTTCT	TCAAGCAGCAGCAA	GATCTTTGTCATCC	GGTAATAGTACCGTTG	CACGGACGAAACCG
**Genome**	**Sample year**	**Pango lineage (Nextclade)**	**Clade**	**WHO variant**	Mismatches Total alignment	Mismatches Total alignment	Mismatches Total alignment	Mismatches Total alignment	Mismatches Total alignment
MN908947	2019	B	19A	basal early SARS-CoV-2 lineage	0/14 100%	0/14 100%	0/14 100%	0/16 100%	0/14 100%
MZ544367	2020	B.1.1	20B	early lineage	0/14 100%	0/14 100%	0/14 100%	0/16 100%	0/14 100%
OV264082	2021	B.1.1.7	20I	Alpha	0/14 100%	0/14 100%	0/14 100%	0/16 100%	0/14 100%
OU205208	2021	B.1.617.2	21A	Delta	0/14 100%	0/14 100%	0/14 100%	0/16 100%	1/13 92%
OQ521295	2021	AY.4	21J	Delta	0/14 100%	0/14 100%	0/14 100%	0/16 100%	1/13 92%
OQ586645	2022	BA.2.2.1	21L	Omicron	0/14 100%	0/14 100%	0/14 100%	0/16 100%	0/14 100%
OY265039	2022	BA.5.2	22B	Omicron	0/14 100%	0/14 100%	0/14 100%	0/16 100%	0/14 100%
PP330140	2023	JN.1.44	24A	Omicron	0/14 100%	0/14 100%	0/14 100%	0/16 100%	0/14 100%
OZ087455	2024	KP.2	24B	Omicron	0/14 100%	0/14 100%	0/14 100%	0/16 100%	0/14 100%
PX399068	2025	NB.1.8.1	25B	Omicron	0/14 100%	0/14 100%	0/14 100%	0/16 100%	0/14 100%
PZ623340	2026	XGF.1.1	25C	Omicron	0/14 100%	0/14 100%	0/14 100%	0/16 100%	0/14 100%

Table S4. Oligonucleotides and templates used for assembly of pBAC-SARS-CoV-2_ *S/eGFP_ΔE* replicon

Primer name	Primer sequence	Amplified sequence	Template
F.1.1.F	ACGCGTGGGACCCGCGATCGCGGCGCGCCAAGCTTGGCGTAATCATG GTCATAGCTG	pUC19-MST	pUC19
F.1.1.R	ACGCGTTAATTAACGCCGGCGCGTACGAGCTCGTCGACGGCGCCTGA TGCGGTATTTTCTC	pUC19-MST	pUC19
F.1.2.VF	GTTTAGTGAACCGTCGTACGCGCCGGCG	pUC19-MST	pUC19-(F1:MCS)
F.1.2.VR	TGTCAACGTCGACGGCGCCTGATG	pUC19-MST	pUC19-(F1:MCS)
F.1.2.IF	CCGTCGACGTTGACATTGATTATTGACTAGTTATTAATAGTAATCAAT TACGG	CMV	pcDNA3.1 CMV mCherry-eGFP BGH
F.1.2.IR	GACGGTTCACTAAACGAGCTCTGCTTATATAGACCTCCC	CMV	pcDNA3.1 CMV mCherry-eGFP BGH
F.1.3.VF	ACGTGCTCGTACGCGCCGGC	pUC19-CMV-MST	pUC19-(F1:CMV-MCS)
F.1.3.VR	CCTTTAATACGGTTCACTAAACGAGCTCTGCT	pUC19-CMV-MST	pUC19-(F1:CMV-MCS)
F.1.3.IF	GAACCGTATTAAAGGTTTATACCTTCCCAGGTAACAAACC	5'UTR-*ORF1*[86]	cDNA SARS-CoV-2 strain Slovakia/SK-BMC5/2020
F.1.3.IR	CGCGTACGAGCACGTCGC	5'UTR-*ORF1*[86]	cDNA SARS-CoV-2 strain Slovakia/SK-BMC5/2020
F.2F	CGTGCTCGTACGTGGCTTT	F2	cDNA SARS-CoV-2 strain Slovakia/SK-BMC5/2020
F.2R	AAGGCGCCGGCGACACCTCCTCCATGTTTAAGGTAAACA	F2	cDNA SARS-CoV-2 strain Slovakia/SK-BMC5/2020
F.3F	GTGTCGCCGGCGCCTTAAATAAGGCTACTAACAATGCCA	F3	cDNA SARS-CoV-2 strain Slovakia/SK-BMC5/2020
F.3R	GTAACTTTAATTAACTGCTTCAACCAATTATTAACAATTTTACCAC	F3	cDNA SARS-CoV-2 strain Slovakia/SK-BMC5/2020
F.4F	GAAGCAGTTAATTAAAGTTACACTTGTGTTCCTT	F4	cDNA SARS-CoV-2 strain Slovakia/SK-BMC5/2020
F.4R	GCGTATACGCGTAATATATCTGGGTTTTCT	F4	cDNA SARS-CoV-2 strain Slovakia/SK-BMC5/2020
F.5F	ATATTACGCGTATACGCCAACTTAGGTG	F5	cDNA SARS-CoV-2 strain Slovakia/SK-BMC5/2020

F.5R	TCTAGGGTCCCCTTAGTTAGCAATGTGCGTGG	F5	cDNA SARS-CoV-2 strain Slovakia/SK-BMC5/2020
F.6F	TAAGGGGACCCTAGAACCAGAATATTTCAATTCAGTGTGTAGACT	F6	cDNA SARS-CoV-2 strain Slovakia/SK-BMC5/2020
F.6R	CCATTGCGATCGCTAGTGGCAATAAAACAAGAAAAACAAACATTGTTC	F6	cDNA SARS-CoV-2 strain Slovakia/SK-BMC5/2020
F.7.2.1F	GGAGAATGACAAAAAAAAAAAAAAAAAAAAAAAAAAAAAAAAAGGGTCGGCATGGC	F7.2poly(A)-pET-21(+)	F7.2-pET-21(+)
F.7.2.1R	TTTTTGTCATTCTCCTAAGAAGCTATTAAAATCACATGGG	F7.2poly(A)-pET-21(+)	F7.2-pET-21(+)
F.7.I1F	TCTAGGATCCAGAAGGCTATGCCTTCGAACAT	*ORF1*-1148-3'	cDNA SARS-CoV-2 strain Slovakia/SK-BMC5/2020
F.7.I1R	TTGATTTGACCTTCTTTTAAAGACATAACAGCAGTAC	*ORF1*-1148-3'	cDNA SARS-CoV-2 strain Slovakia/SK-BMC5/2020
F.7.I2F	ATGTCTTTAAAAGAAGGTCAAATCAATGATATGAT	F7.1, F7	pTwist-Amp-MC-F7.2
F.7.I2R	GTTTAATCAATCTCCATTGGTTGCT	F7.1	pTwist-Amp-MC-F7.2
F.7.VF	AGCAACCAATGGAGATTGATTAAACGAACA	F7.2poly(A)-pET-21(+)	F7.2poly(A)-pET-21(+)
F.7.VR	GCATAGCCTTCTGGATCCTAGAGGGGAATTGTTATC	F7.2poly(A)-pET-21(+)	F7.2poly(A)-pET-21(+)
F.7R	GAAACAGCTATGACCATGATTACGCC	F7	F7-pET-21(+)

Table S5. Primers used for reverse transcription and qPCR assay

Primers name	Primer sequence
**Reverse Transcrition**	
Anti-Leader-RT	TTTATACCTTCCCAGGTAAC
GAPDEH-RT	CTGTTGCTGTAGCCAAATTCG
3UTR-RT	CTCCTAAGAAGCTATTAAAATCAC
**qPCR**	
Leader_For	CCAACCAACTTTCGATCTCTTGTAGATCTG
qRNA_Rev	CAGGCAAACTGAGTTGGACGTG
N_Rev	TTGGGTAAACCTTGGGGCCG
M_Rev	CCTATTACTAGGTTCCATTGTTCAAGGAGC
GAPDEH_For	GAAGGTGAAGGTCGGAGTC
GAPDEH_Rev	GAAGATGGTGATGGGGCTTC
MX1_For	AGGACCATCGGAATCTTGAC
MX1_Rev	TCAGGTGGAACACGAGGTTC
